# Chemical synthesis of glycan motifs from the antitumor agent PI-88 through an orthogonal one-pot glycosylation strategy

**DOI:** 10.3762/bjoc.21.122

**Published:** 2025-08-06

**Authors:** Shaokang Yang, Xingchun Sun, Hanyingzi Fan, Guozhi Xiao

**Affiliations:** 1 School of Chemistry and Chemical Engineering, Shaanxi Normal University, Xi’an, Shaanxi 710119, China; 2 State Key Laboratory of Phytochemistry and Natural Medicines, Kunming Institute of Botany, University of Chinese Academy of Sciences, Chinese Academy of Sciences, Kunming 650201, China

**Keywords:** carbohydrates, chemical synthesis, glycosyl *ortho*-(1-phenylvinyl)benzoates, one-pot glycosylation, PI-88

## Abstract

Chemical synthesis of monophosphorylated glycan motifs from the antitumor agent PI-88 has been achieved through an orthogonal one-pot glycosylation strategy on the basis of glycosyl *ortho*-(1-phenylvinyl)benzoates, which not only accelerated synthesis, but also precluded the potential issues inherent to one-pot glycan assembly associated with thioglycosides. The following aspects were featured in synthetic approaches: 1) synthesis of trisaccharide and tetrasaccharide PI-88 glycans via [1 + 1 + 1] and [1 + 1 + 1 + 1] one-pot orthogonal glycosylation, respectively; 2) synthesis of PI-88 glycan motif pentasaccharide via [1 + 1 + 1] and [1 + 1 + 3] one-pot orthogonal glycosylation; 3) synthesis of hexasaccharide via [1 + 1 + 1] and [1 + 1 + 1 + 3] one-pot assembly.

## Introduction

**Scheme 1:**
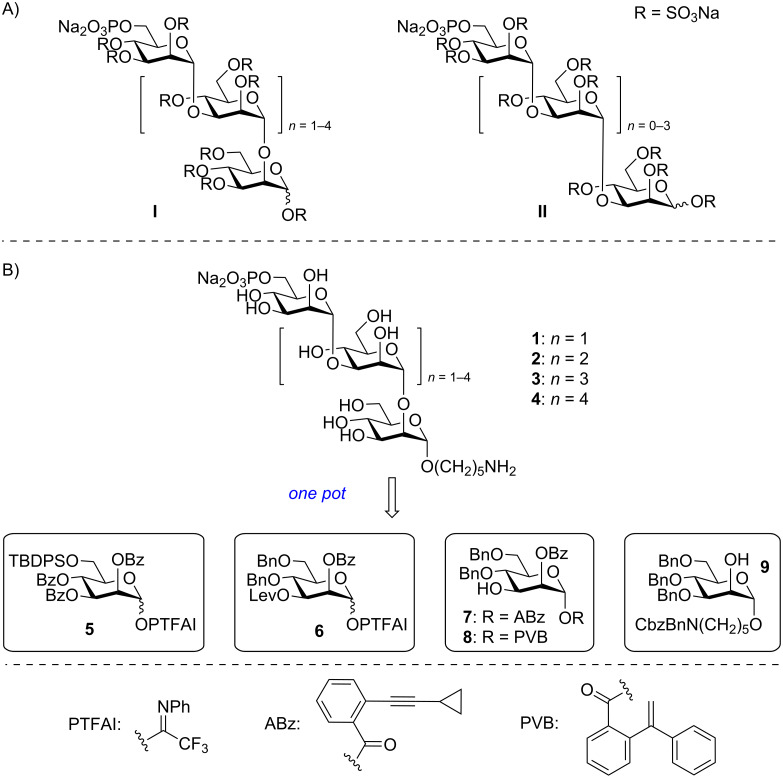
(A) Glycan structures of PI-88 and (B) retrosynthetic analysis of PI-88 glycan motifs **1**–**4**.

Carbohydrates as one of four essential biomolecules have been widely recognized as important targets for the development of carbohydrate-based therapeutics [1–18]. The example in point is the antitumor agent PI-88 (muparfostat), which retards tumor growth via inhibiting angiogenesis in two ways: 1) interaction with pro-angiogenic growth factors such as vascular endothelial growth factor (VEGF) and fibroblast growth factor (FGF) and 2) by prevention of the release of angiogenic growth factors from the extracellular matrix (ECM) via inhibition of heparanase [19–22]. PI-88 is a complex mixture of monophosphorylated, highly sulfated mannose glycans derived from the extracellular phosphomannan of *Pichia holstii* NRRL Y-2448 yeast [23–25], which had progressed to phase III clinical trials for post-resection hepatocellular carcinoma [26]. Interestingly, Ferro and co-workers revised the structure of PI-88 to **I** and **II** in 2017 via successful separation of oligosaccharide phosphate fractions by preparative ion-exchange chromatography (Scheme 1A) [27]. Besides the major components α(1→3)/α(1→2)-linked pentasaccharide (≈60%) and tetrasaccharide (≈30%) in **I**, the minor components of all α(1→3)-linked mannosides were also present in **II**.

During the past two decades, several strategies have been developed to synthesize glycan motifs from PI-88 [28–36]. In comparison with previous, traditional, and time-consuming synthesis of PI-88 glycan components, the one-pot glycan assembly strategy has some advantages, including: 1) acceleration of glycan synthesis, 2) avoidance of purification of intermediates during glycosylation intervals, and 3) reduction of chemical waste [37–42]. Recently, we introduced a new one-pot glycosylation strategy on the basis of recently developed glycosyl *ortho*-(1-phenylvinyl)benzoate (PVB) [43–45] donors from our group, which has been successfully applied to the streamline synthesis of various glycans from oligosaccharides to polysaccharides such as mannose-capped lipoarabinomannan motifs up to 101-mer from the *Mycobacterium tuberculosis* cell wall, nona-decasaccharide motif from *Ganoderma sinense*, and tridesaccharide motif from *Bacteroides vulgatus* lipopolysaccharides [46–56]. Here, we report the chemical synthesis of monophosphorylated glycan motifs **1**–**4** from PI-88 through an orthogonal one-pot glycosylation strategy via strategic combinations of glycosyl *N*-phenyltrifluoroacetimidates (PTFAI) [57–58], glycosyl *ortho*-(alkynylbenzoates) [59–60] (ABz), and glycosyl PVB, which precluded the potential issues inherent to one-pot glycosylation based on thioglycosides such as aglycone transfer [43–45,61].

## Results and Discussion

### Retrosynthetic analysis

Retrosynthetically, we envisaged that glycans **1**–**4** could be derived from monosaccharide building blocks Man PTFAI **5** and **6**, Man ABz **7**, Man PVB **8**, and Man **9** through orthogonal one-pot glycosylation strategy (Scheme 1B). The 2-*O*-Bz group in **5**–**8** served as the neighboring participating group for the stereoselective construction of 1,2-*trans*-mannosidic bonds, while the 3-*O*-Lev group in **6** was the temporary protecting group for (1→3)-branching. The C6–OH group in **5** was protected as TBDPS group, which could be selectively replaced by the destined phosphate residue.

### One-pot synthesis of glycans **1** and **2**

**Scheme 2:**
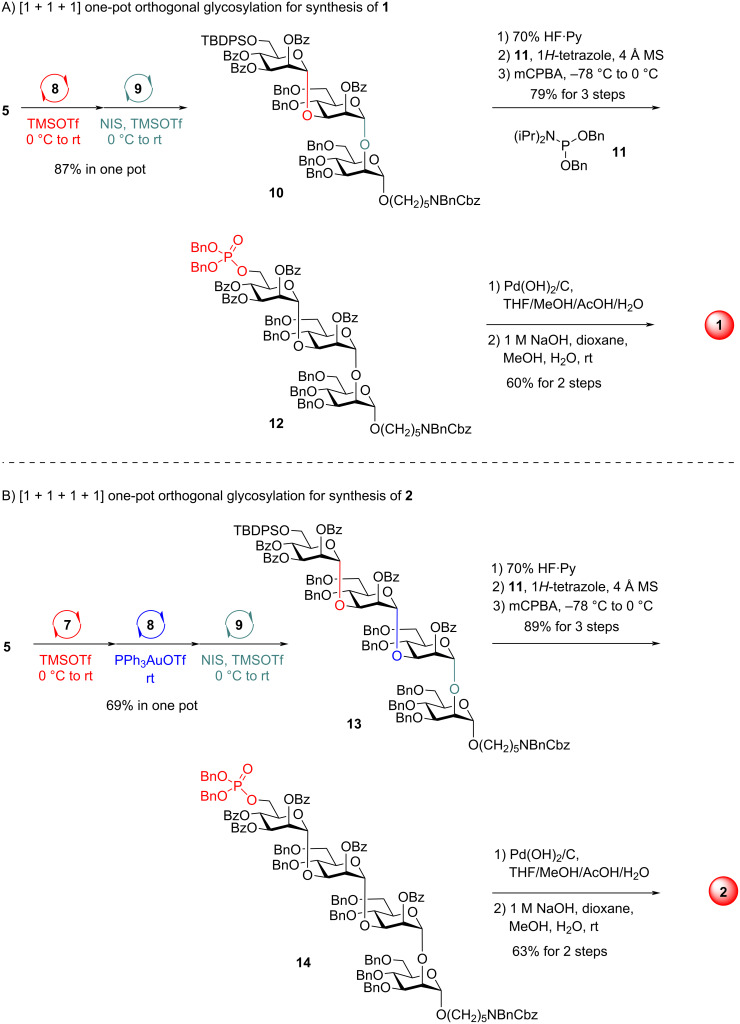
One-pot synthesis of glycans **1** and **2**.

We commenced with the synthesis of monophosphorylated trisaccharide **1** (Scheme 2A). Glycosylation of mannosyl PTFAI **5** (1.2 equiv) with 3-OH in mannosyl PVB **8** (1.0 equiv) in the presence of TMSOTf as catalyst proceeded smoothly at 0 °C to room temperature, affording the α-Man-(1→3)-Man PVB disaccharide. The further coupling of the above PVB disaccharide with the poorly reactive 2-OH in mannoside **9** (0.9 equiv) under activation with NIS and TMSOTf at 0 °C to room temperature, successfully furnished the desired α-Man-(1→3)-α-Man-(1→3)-α-Man trisaccharide **10** in 87% yield in a one pot manner. Removal of TBDPS group in **10** with 70% HF·pyridine and subsequent phosphitylation of the resulting free alcohol with phosphoramidite **11** provided the desired phosphite, which was further oxidized by 3-chloroperoxybenzoic aicd (mCPBA) at −78 °C to 0 °C, producing the desired phosphorylated fully protected trisaccharide **12** in 79% overall yield over three steps. Removal of all protecting groups in trisaccharide **12** is a challenging task due to the presence of polar groups, including phosphoryl acid and amine groups [62]. After several optimizations, the following sequence was adopted to remove all Bn, Bz, and Cbz groups: 1) global hydrogenolysis of Bn and Cbz groups in **12** with Pd(OH)_2_/C in a mixed solvent (THF/MeOH/AcOH/H_2_O) and 2) saponification of all Bz groups with 1 M NaOH (dioxane/MeOH/H_2_O, room temperature). The monophosphorylated trisaccharide **1** was obtained in 60% overall yield over two steps from **12** after purification over a Sephadex^TM^ LH-20 column. It was noted that the switch of deprotection sequences (first Bz groups, second Bn and Cbz groups) failed to efficiently produce trisaccharide **1**.

The synthesis of monophosphorylated tetrasaccharide **2** was next investigated (Scheme 2B). TMSOTf was used to activate Man PTFAI **5** (1.1 equiv) in the presence of mannosyl ABz **7** (1.0 equiv) at 0 °C to room temperature, readily producing the α-Man-(1→3)-Man ABz disaccharide. Yu glycosylation of the above ABz disaccharide with 3-OH in Man PVB **8** (0.9 equiv) under the catalysis of PhP_3_AuOTf at room temperature successfully gave α-Man-(1→3)-α-Man-(1→3)-Man PVB trisaccharide, which was further coupled with the poorly reactive C2–OH in mannoside **9** (0.8 equiv) in the presence of NIS and TMSOTf at 0 °C to rt, uneventfully furnishing the desired tetrasaccharide α-Man-(1→3)-α-Man-(1→3)-α-Man-(1→2)-α-Man **13** in 69% yield in the same flask. The TBDPS-protected **13** was readily converted to phosphorylated protected tetrasaccharide **14** in 89% ovall yield over the following steps: 1) deprotection of the TBDPS group, 2) phosphitylation of the free alcohol with phosphoramidite **11** in the presence of 1*H*-tetrazole and 4 Å MS, and 3) oxidation of the phosphite by mCPBA. Hydrogenolysis of Bn and Cbz groups in **14** with Pd(OH)_2_/C and subsequent saponification of all Bz groups with 1 M NaOH successfully produced monophosphorylated tetrasaccharide **2** in 63% overall yield.

### One-pot synthesis of glycans **3** and **4**

**Scheme 3:**
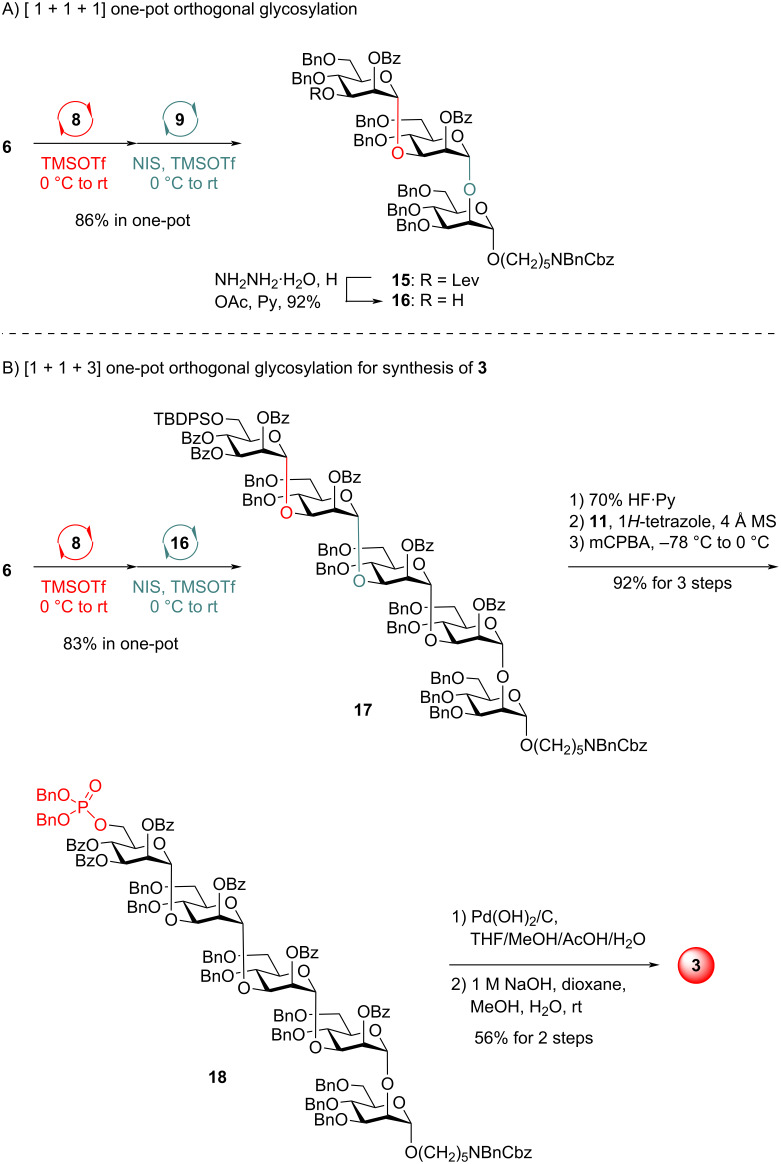
One-pot synthesis of glycan **3**.

Furthermore, we investigated the synthesis of monophosphorylated pentasaccharide **3** (Scheme 3). Orthogonal one-pot glycosylation of Man PTFAI **6** (1.2 equiv), Man PVB **8** (1.0 equiv), and mannoside **9** (0.9 equiv) readily generated α-Man-(1→3)-α-Man-(1→2)-α-Man trisaccharide **15** with 86% yield in one pot. The further sequential [1 + 1 + 3] one-pot orthogonal glycosylation of Man PTFAI **5** (1.1 equiv), Man PVB **8** (1.0 equiv), and trisaccharide **16** (0.9 equiv) derived from **15** via selective removal of the Lev group with NH_2_NH_2_·H_2_O successfully generated the desired pentasaccharide α-Man-(1→3)-α-Man-(1→3)-α-Man -(1→3)-α-Man-(1→2)-α-Man **17** in 83% yield in a one-pot manner, which was readily converted to the phosphorylated protected pentasaccharide **18** in 92% overall yield via the switch of the TBDPS group with the phosphate group. First global deprotection of Bn and Cbz groups in **18** with Pd(OH)_2_/C, followed by saponifications of all Bz groups with 1 M NaOH provided the desired monophosphorylated pentasaccharide **3** in 56% overall yield, which is the major glycan motif from PI-88.

**Scheme 4:**
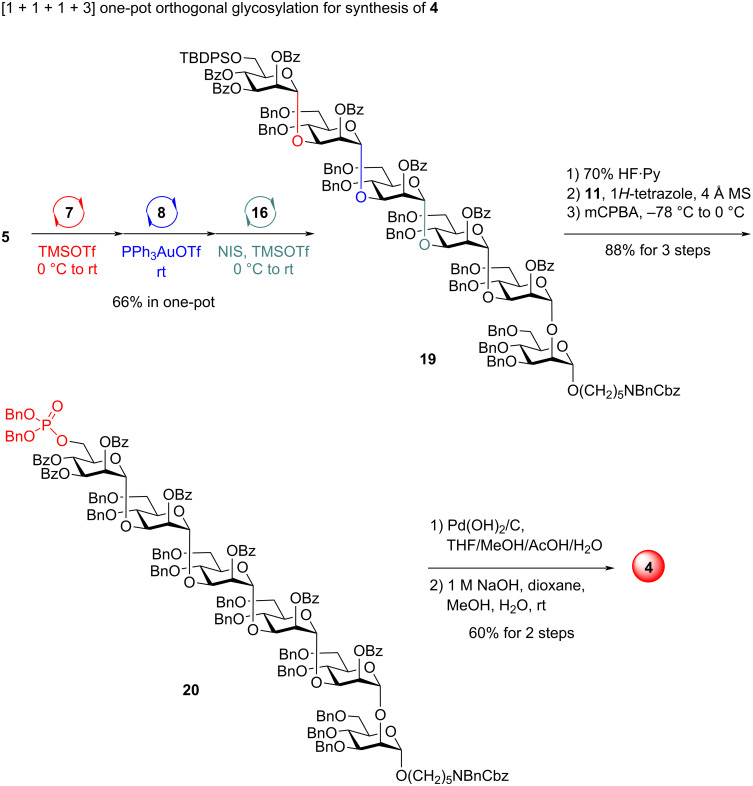
One-pot synthesis of glycan **4**.

Finally, the synthesis of the monophosphorylated hexasaccharide **4** was studied (Scheme 4). Orthogonal one-pot coupling of Man PTFAI **5** (1.1 equiv), Man ABz **7** (1.0 equiv), PVB **8** (0.9 equiv), and α-Man-(1→3)-α-Man-(1→2)-α-Man trisaccharide **16** (0.8 equiv) proceeded uneventfully, successfully producing the desired α-Man-(1→3)-α-Man-(1→3)-α-Man-(1→3)-α-Man-(1→3)-α-Man-(1→2)-α-Man hexasaccharide **19** in 66% yield in the same flask. The TBDPS group in **19** was readily converted to a phosphate group in **20** with 88% overall yield over three steps. The desired monophosphorylated hexasaccharide **4** was obtained in 60% overall yield from **20** via sequential global deprotection of the Bn, Cbz, and Bz groups.

The structures of the synthetic glycan motifs **1**–**4** were supported by their ^1^H and ^13^C NMR spectra and MALDI–TOF as well as ESI mass spectra. In particular, the anomeric proton signals of **1**–**4** were highlighted in the ^1^H NMR spectra of synthetic glycans motifs **1**–**4** (see Supporting Information File 1).

## Conclusion

In summary, the monophosphorylated glycan motifs **1**–**4** from PI-88 have been collectively synthesized via a one-pot orthogonal glycosylation strategy on the basis of glycosyl PVB, which avoids such issues as aglycon transfer inherent to one-pot glycosylations based on thioglycosides. Specifically, the following features were highlighted in our synthetic approach: 1) [1 + 1 + 1] one-pot orthogonal glycosylation for the synthesis of trisaccharide **1**; 2) [1 + 1 + 1 + 1] orthogonal one-pot glycosylation for the synthesis of tetrasaccharide **2**; 3) [1 + 1 + 1] and [1 + 1 + 3] orthogonal one-pot assembly of pentasaccharide **3**; 4) [1 + 1 + 1] and [1 + 1 + 1 + 3] orthogonal one-pot assembly of hexasaccharide **4**.

## Supporting Information

File 1 Experimental procedures and spectral data for all new compounds including ^1^H NMR, ^13^C NMR, and HRMS.

## Data Availability

All data that supports the findings of this study is available in the published article and/or the supporting information of this article
