# Supplementary material for: Chemical synthesis of glycan motifs from the antitumor agent PI-88 through an orthogonal one-pot glycosylation strategy
Source: Beilstein J Org Chem. 2025 Aug 6;21:1587–94. doi: 10.3762/bjoc.21.122 (PMC12337991; doi:10.3762/bjoc.21.122)
Supplement: File 1 — Experimental procedures and spectral data for all new compounds including 1H NMR, 13C NMR, and HRMS. [file Beilstein_J_Org_Chem-21-1587-s001.pdf]

BEILSTEIN JOURNAL OF ORGANIC CHEMISTRY

Supporting Information

for

Chemical synthesis of glycan motifs from the antitumor agent PI-88 through an orthogonal one-pot glycosylation strategy

Shaokang Yang, Xingchun Sun, Hanyingzi Fan and Guozhi Xiao

Beilstein J. Org. Chem. **2025,** *21,* 1587–1594. doi:10.3762/bjoc.21.122

Experimental procedures and spectral data for all new compounds including ^{1}H NMR, ^{13}C NMR, and HRMS

License and Terms: This is a supporting information file under the terms of the Creative Commons Attribution License (https://creativecommons.org/licenses/by/4.0). Please note that the reuse, redistribution and reproduction in particular requires that the author(s) and source are credited and that individual graphics may be subject to special legal provisions.
The license is subject to the *Beilstein Journal of Organic Chemistry* terms and conditions: (https://www.beilstein-journals.org/bjoc/terms)

Table of contents

1. General methods S2

2. Synthesis of monosaccharides building blocks **5–9** S3

3. One-pot orthogonal synthesis of PI-88 glycan motif **1** S8

4. One-pot orthogonal synthesis of PI-88 glycan motif **2** S12

5. One-pot orthogonal synthesis of PI-88 glycan motif **3** S16

6. One-pot orthogonal synthesis of PI-88 glycan motif **4** S22

7. Confirmation of the structures of PI-88 glycan motifs **1–4** S27

8. References S28

9. Spectroscopic data S29

1. General methods

All reactions were carried out under an argon atmosphere with dry solvents under anhydrous conditions, unless otherwise noted. All glycosylation reactions were performed in the presence of 3 Å or 4 Å molecular sieves, which were flame-dried immediately before use in the reaction under high vacuum. Tetrahydrofuran (THF) was distilled immediately before use from sodium benzophenonketyl. Methylene chloride (DCM) was distilled from calcium hydride and stored under an argon atmosphere. Toluene was distilled immediately from calcium chloride before use. Reagents were purchased at the highest commercial quality and used without further purification, unless otherwise stated. Reactions were monitored by thin-layer chromatography (TLC) carried out on TLC Silica gel 60 F254 or TLC Silica gel 60 RP-18 F254S (EMD Millipore Corporation) using UV light (254 nm) as visualizing agent and 10% H_2SO_4/EtOH solution as developing agent. Flash column chromatography was performed on silica gel or Sephadex TM LH-20 (GE Healthcare). The ^{1}H NMR, ^{13}C NMR, H-H COSY, and HSQC spectra were measured by a Bruker AVANCE III 400MHz spectrometer, Bruker Avance III 600MHz spectrometer or Bruker AV 800MHz spectrometer by using $CDCl_3$ or D_2O as internal references: $CDCl_3$ (^{1}H NMR δ = 7.26 ppm, ^{13}C NMR δ = 77.16 ppm) or D_2O (^{1}H NMR δ = 4.79 ppm). The following abbreviations are used to designate multiplicities: s = singlet, d = doublet, t = triplet, q =quartet, m = multiplet. Structural assignments were made with additional information from gCOSY, gHSQC, and gHMBC experiments. Electron-spray ionization (ESI) and high-resolution electron spray ionization (HRESI) were obtained on an Agilent 1290 spectrometer. MALDI-TOF spectra were recorded on a New ultrafleXtreme. The specific rotation was obtained on a Jasco P-1020, using $CHCl_3$ or H_2O as solvent.

2. Synthesis of monosaccharides building blocks 5–9

O-(2,3,4-Tri-O-benzoyl-6-O-tert-butyldiphenylsilyl-D-mannopyranoside)-N-phenyltrifluoroacetimidate (5)

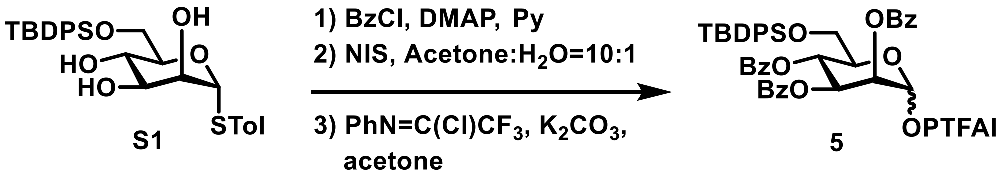

To a solution of **S1**[1] (6.74 g, 13.20 mmol) and 4-dimethylaminopyridine (1.62 g, 13.20 mmol) in pyridine (26.4 mL) was added BzCl (9.2 mL, 79.20 mmol) at 0 °C. The resulting mixture was stirred at 0 °C to room temperature for 6 h. The solution was diluted with DCM, washed with 4 mol/L HCl and saturated $NaHCO_3$ solution, dried over anhydrous Na_2SO_4, filtered and concentrated in vacuo. Then, the residue was purified by flash column chromatography (PE/EA 8:1 to 6:1) to give the intermediate (9.99 g, 90%). To the above intermediate (1.0 g, 0.12 mmol) in mixed solvent of acetone/H_2O 10:1 (10.5 mL) was added NIS (1.34 g, 0.60 mmol). After stirring for 22 h at room temperature, the residue was purified by flash column chromatography (PE/EA 8:1 to 4:1) to give the intermediate (750 mg, 86%). The above hemiacetal (100 mg, 0.14 mmol) was dissolved in acetone (1.4 mL), followed by the addition of 2,2,2-trifluoro-*N*-phenylacetimidoyl chloride (34.2 mg, 0.16 mmol) and potassium carbonate (18.9 mg, 0.14 mmol). The reaction mixture was stirred overnight at room temperature. Upon completion, the reaction mixture was filtered and concentrated in vacuo. The residue was purified by column chromatography on silica gel (PE/EA 20:1 to 10:1, Et_3N 2%) to give the **5** as a light yellow syrup (120 mg, 97%). α isomer: $[\alpha]_D^{20}$= -99.67 (*c* 0.30 $CHCl_3$); ^1H NMR (400 MHz, $CDCl_3$) δ 8.11 (d, *J* = 6.8 Hz, 2H), 7.95 (d, *J* = 7.0 Hz, 2H), 7.87 (d, *J* = 8.3 Hz, 2H), 7.71 (d, *J* = 6.7 Hz, 2H), 7.64 – 7.51 (m, 4H), 7.49 – 7.36 (m, 5H), 7.37 – 7.23 (m, 8H), 7.18 – 7.08 (m, 3H), 6.86 (d, *J* = 7.7 Hz, 2H), 6.56 (s, 1H), 6.31 (t, *J* = 10.2 Hz, 1H), 5.94 (t, *J* = 2.5 Hz, 1H), 5.87 (dd, *J* = 10.2, 3.4 Hz, 1H), 4.25 (d, *J* = 10.1 Hz, 1H), 3.89 (t, *J* = 2.7 Hz, 2H), 1.07 (s, 9H). ^{13}C NMR (101 MHz, $CDCl_3$) δ 165.6, 165.3, 165.1, 143.1, 135.7,

135.5, 133.6, 133.4, 133.3, 132.9, 132.6, 130.1, 129.8, 129.7, 129.6, 129.2, 129.0, 128.8, 128.6, 128.5, 128.4, 127.7, 127.6, 124.6, 119.5, 74.0, 70.25, 69.0, 65.6, 62.0, 26.6, 19.2. HRMS (ESI) m/z: $[M+Na]^+$ Calcd for $C_{51}H_{46}F_3NO_9SiNa$ 924.2786; Found 924.2791.

***O*-(2-*O*-Benzoyl-3-*O*-levulinoyl-4,6-di-*O*-benzyl)-*N*-phenyltrifluoroacetimidate (6)**

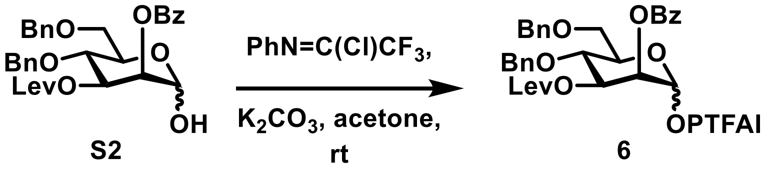

To a solution of **S2**[2] (2.7 g, 4.80 mmol) in acetone (1.4 mL) was added 2,2,2-trifluoro-*N*-phenylacetimidoyl chloride (92.0 mg, 0.44 mmol) and potassium carbonate (87.0 mg, 0.63 mol). After stirring for 3 h at room temperature, the reaction mixture was filtered and concentrated in vacuo. The residue was purified by column chromatography on silica gel (PE/EA 20:1 to 10:1, containing 2% Et_3N) to afford PTFAI donor **6** as a yellow syrup (3.67 g, α/β = 8:1, 94%). α isomer: $[\alpha]_D^{25}$= +5.40 (*c* 0.50 $CHCl_3$). ^1H NMR (400 MHz, $CDCl_3$) δ 8.06 (d, *J* = 7.7 Hz, 2H), 7.61 (t, *J* = 7.2 Hz, 1H), 7.49 – 7.18 (m, 14H), 7.10 (t, *J* = 7.3 Hz, 1H), 6.83 (d, *J* = 7.9 Hz, 2H), 6.40 (s, 1H), 5.74 (s,, 1H), 5.53 (dd, *J* = 9.8, 3.3 Hz, 1H), 4.76 (t, *J* = 11.2 Hz, 2H), 4.67 – 4.53 (m, 2H), 4.31 (t, *J* = 9.8 Hz, 1H), 4.12 – 4.08 (m, 1H), 3.97 (dd, *J* = 11.2, 3.5 Hz, 1H), 3.83 (d, *J* = 11.1 Hz, 1H), 2.78 (m, 1H), 2.70 – 2.60 (m, 1H), 2.59 – 2.38 (m, 2H), 2.13 (s, 3H). ^{13}C NMR (101 MHz, $CDCl_3$) δ 206.2, 171.9, 165.3, 143.2, 142.4, 142.1, 138.3, 137.8, 133.7, 133.5, 130.1, 130.0, 129.4, 129.25, 129.23, 128.9, 128.7, 128.6, 128.53, 128.51, 128.2, 128.04, 128.01, 127.74, 127.68, 127.5, 126.3, 124.6, 120.73, 120.68, 119.48, 94.1, 75.2, 74.3, 73.6, 72.2, 72.0, 68.8, 68.5, 60.5, 45.9, 37.9, 29.9, 28.0, 21.1, 14.3, 9.0. HRMS (ESI) m/z: [M+Na]+ Calcd for $C_{40}H_{38}F_3NO_9SiNa$ 756.2391; Found 756.2393.

O-(2-O-Benzoyl-4,6-di-O-benzyl-D-mannopyranosyl)-o-cyclopropylethynylbenzoate(7)

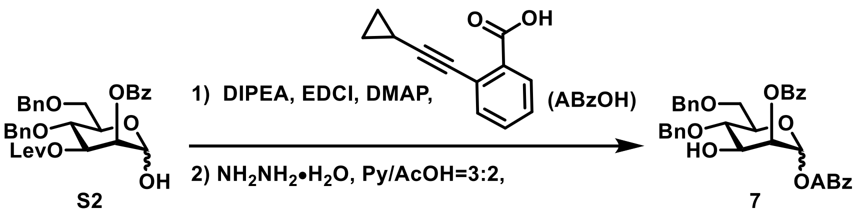

The compound **S2** (3.55 g, 6.3 mmol) was dissolved in DCM (21 mL), followed by the addition of ABzOH (1.76 g, 9.45 mmol), EDCI (2.17 g, 47.31 mmol), DMAP (770 mg, 6.30 mmol) and DIPEA (3.1 mL, 18.90 mmol). Upon completion, the reaction mixture was concentrated, and the residue was purified by column chromatography on silica gel (PE/EA 4:1) to give the intermediate (4.03 g, 87%). A solution of the above intermediate (4.02 g, 5.50 mmol) was then dissolved in pyridine/AcOH (16.5 mL/11.0 mL), and N_2H_4•H_2O (1.0 mL, 16.50 mmol) was added successively at room temperature, and the mixture stirred at room temperature overnight and quenched with acetone (2.0 mL). The residue was dissolved in DCM, washed with 4 M HCl, saturated $NaHCO_3$ solution and brine. The organic solution was dried over anhydrous Na_2SO_4, filtered and concentrated. The residue was purified by flash column chromatography on silica gel (PE/EA 4:1) to afford **7** as a white foam (3.31 g, α/β= 5:4, 83%). α isomer: $[\alpha]_D^{25}$= -5.42 (*c* 0.24 $CHCl_3$); ^{1}H NMR (400 MHz, $CDCl_3$) δ 8.07 (d, *J* = 7.2 Hz, 2H), 7.92 (d, *J* = 8.6 Hz, 1H), 7.59 (t, *J* = 7.4 Hz, 1H), 7.49 – 7.43 (m, 2H), 7.42 – 7.38 (m, 4H), 7.36 – 7.31 (m, 4H), 7.30 – 7.25 (m, 5H), 6.51 (d, *J* = 1.9 Hz, 1H), 5.59 – 5.55 (m, 1H), 4.80 (dd, *J* = 11.5, 8.3 Hz, 2H), 4.69 (d, *J* = 11.0 Hz, 1H), 4.60 – 4.51 (m, 2H), 4.19 (t, *J* = 9.6 Hz, 1H), 4.11 – 4.06 (m, 1H), 3.97 (dd, *J* = 11.4, 3.2 Hz, 1H), 3.82 (dd, *J* = 11.4, 1.8 Hz, 1H), 2.21 (d, *J* = 4.6 Hz, 1H), 1.60 – 1.57 (m, 1H), 0.85 – 0.79 (m, 4H). ^{13}C NMR (400 MHz, $CDCl_3$) δ 166.1, 164.2, 138.5, 138.2, 134.8, 133.6, 132.3, 131.1, 130.6, 130.2, 129.6, 128.6, 128.5, 128.2, 128.1, 127.8, 127.7, 127.3, 125.0, 100.1, 92.0, 75.3, 75.2, 74.9, 74.3, 73.8, 71.9, 70.7, 68.8, 9.2, 9.1, 0.8. HRMS (ESI) calcd for $C_{39}H_{36}O_8Na$ $[M+Na]^+$ 655.2302 found 655.2302.

***O*-(2-*O*-Benzoyl-4,6-di-*O*-benzyl-D-mannopyranosyl)-2-(1-phenylvinyl) benzoate (8)**

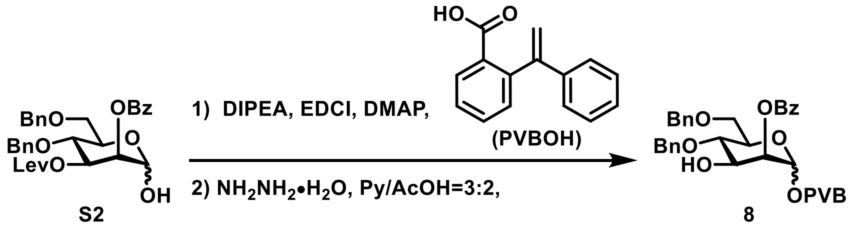

The compound **S2** (5.0 g, 8.89 mmol) was dissolved in DCM (29.6 mL), followed by the addition of PVBOH (2.99 g, 13.33 mmol), EDCI (3.06 g, 15.99 mmol), DMAP (1.08 g, 8.89 mmol), and DIPEA (4.4 mL, 26.66 mmol). Upon completion, the reaction mixture was concentrated and the residue was purified by column chromatography on silica gel (PE/EA 4:1) to give the intermediate (5.89 g, 86%). A solution of the above intermediate (5.89 g, 7.66 mmol) was then dissolved in pyridine/AcOH (23.0 mL/ 15.0 mL) and N_2H_4•H_2O (1.4 mL, 22.98 mmol) was added at room temperature, and the mixture stirred at room temperature overnight and quenched with acetone (2.0 mL). The residue was dissolved in DCM, washed with 4 M HCl, $NaHCO_3$ solution and brine. The organic solution was dried over anhydrous Na_2SO_4, filtered and concentrated. The residue was purified by flash column chromatography on silica gel (PE/EA 3:1) to afford **8** as a white foam (4.9 g, 95%, α/β = 1:1). α isomer: $[\alpha]_D^{25}$= +2.29 (*c* 0.41 $CHCl_3$); ^{1}H NMR (400 MHz, $CDCl_3$) δ 7.96 (d, *J* = 7.7 Hz, 2H), 7.84 (d, *J* = 7.7 Hz, 1H), 7.52 (t, *J* = 7.5 Hz, 2H), 7.40 (t, *J* = 7.6 Hz, 1H), 7.34 – 7.20 (m, 18H), 6.12 (s, 1H, H-1), 5.79 (s, 1H, H-PVB), 5.20 (s, 1H, H-PVB), 5.04 (s, 1H, H-2), 4.68 (dd, *J* = 11.6, 6.0 Hz, 2H), 4.56 (d, *J* = 11.2 Hz, 1H), 4.47 (d, *J* = 11.9 Hz, 1H), 3.93 (t, *J* = 9.6 Hz, 1H), 3.82 – 3.70 (m, 2H, H-3), 3.57 (d, *J* = 10.4 Hz, 2H).^{13}C NMR (101 MHz, $CDCl_3$) δ 166.0, 165.5, 148.8, 142.8, 139.9, 138.6, 138.4, 133.5, 132.3, 131.4, 130.5, 130.1, 129.6, 128.6, 128.6, 128.5, 128.1, 128.0, 128.0, 127.8, 127.7, 127.6, 126.9, 114.4, 92.1, 74.9, 74.9, 74.2, 73.7, 71.5, 70.6, 68.7. HRMS (ESI) calcd for $C_{42}H_{38}O_8Na$ $[M+Na]^+$ 693.2459 found 693.2457.

***N*-Benzyl-*N*-benzyloxycarbonyl-5-aminopentyl 3,4,6-tri-*O*-benzyl-α-D-mannopyranoside (9)**

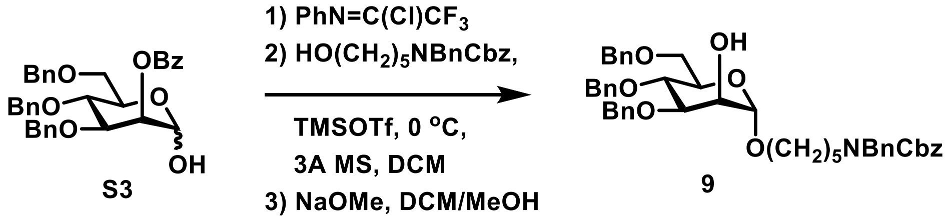

To a solution of **S3**[3] (9.45 g, 17.04 mmol) in acetone (3.3 mL) was added 2,2,2-trifluoro-*N*-phenylacetimidoyl chloride (4.24 g, 20.45 mmol) and potassium carbonate (2.36 g, 17.04 mmol). The mixture was stirred for 3 h at room temperature. Upon completion, the reaction mixture was filtered and concentrated in vacuo. The residue was purified by column chromatography on silica gel (PE/EA 20:1 to 10:1, 2% Et_3N) to afford the intermediate as a light yellow syrup (12.0 g, 97%). A mixture of the above intermediate (3.0 g, 4.14 mmol), $HO(CH_2)_5NBnCbz$ (1.62 g, 4.96 mmo) and 3 Å MS (4.7 g) in dry DCM (41.3 mL) was stirred at room temperature for 15 min and then cooled to 0 °C. Then, TMSOTf (150 μL, 0.83 mmol) was added to the mixture dropwise. After being stirred at 0 °C for another 2 h, the reaction was quenched with Et_3N (3.0 mL) and filtered. The filtrate was concentrated under vacuum to give a residue, which was purified by flash column chromatography (PE/EA 15:1 to 10:1) to give the intermediate (3.43 g, 96%). A solution of the above intermediate (3.43 g, 3.97 mmol) was then dissolved in DCM/MeOH (10 mL/1 mL), followed by the addition of NaOMe to adjust the pH to 10. After stirring for 2 h at room temperature, the mixture was filtrated and concentrated in vacuo. The residue was purified by flash column chromatography (PE/EA 6:1) to give **9** as a white foam (2.16 g, 86%). $[\alpha]_D^{25}$= +35.54 (*c* 0.39 $CHCl_3$). ^{1}H NMR (400 MHz, $CDCl_3$) δ 7.40 – 7.19 (m, 22H), 7.19 – 7.06 (m, 3H), 5.18 (s, 1H), 5.15 (s, 1H), 4.85 (s, 1H), 4.81 (d, *J* = 10.8 Hz, 1H), 4.73 – 4.59 (m, 3H), 4.57 – 4.43 (m, 4H), 4.00 (s, 1H), 3.89 – 3.80 (m, 2H), 3.78 – 3.65 (m, 3H), 3.64 – 3.55 (m, 1H), 3.40 – 3.29 (m, 1H), 3.29 – 3.14 (m, 2H), 2.55 (s, 1H), 1.58 – 1.42 (m, 4H), 1.33 – 1.16 (m, 2H). ^{13}C NMR (101 MHz, $CDCl_3$) δ 156.8, 156.2, 138.4, 138.3, 138.1, 138.0, 136.9, 128.6, 128.6, 128.5, 128.4,

128.4, 128.05, 128.00, 127.94, 127.90, 127.87, 127.8, 127.6, 127.4, 127.2, 99.2, 80.4, 75.2, 74.4, 73.5, 72.0, 71.1, 69.0, 68.5, 67.5, 67.2, 50.6, 50.3, 47.2, 46.2, 29.2, 28.0, 27.6, 23.5. HRMS (ESI) calcd for $C_{47}H_{53}NO_8Na$ $[M+Na]^+$, 782.3663, found 782.3670.

3. One-pot orthogonal synthesis of PI-88 glycan motif 1

***N*-Benzyl-*N*-benzyloxycarbonyl-5-aminopentyl 6-*O*-*tert*-butyldiphenylsilyl-2,3,4-tri-*O*-benzoyl-α-D-mannopyranosyl-(1→3)-2-*O*-benzoyl-4,6-di-*O*-benzyl-α-D-mannopyranosyl-(1→2)-3,4,6-tri-*O*-benzyl-*O*-α-D-mannopyranoside (10)**

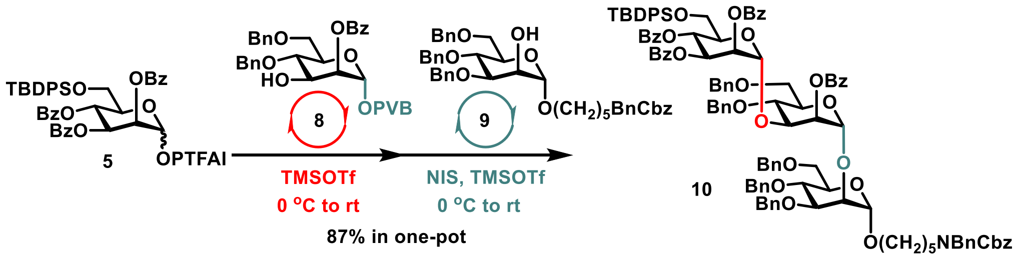

A suspension of donor **5** (645 mg, 0.72 mmol), PVB acceptor **8** (400 mg, 0.60 mmol), activated 3 Å MS (1.5 g) in dry DCM (6.0 mL) was stirred at room temperature for 15 min under N_2 and then cooled to 0 °C. A solution of TMSOTf in DCM (0.5 mL, 52 μL TMSOTf in 1.0 mL of DCM) was slowly added to the mixture. The mixture was stirred for another 2 h. Then, acceptor **9** (408 mg, 0.54 mmol), NIS (202 mg, 0.89 mmol) and a solution of TMSOTf in DCM (0.5 mL, 42 μL TMSOTf in 1.0 mL DCM) were added successively. The resulting mixture was stirred at 0 °C to room temperature for another 4 h, then quenched with Et_3N (0.5 mL) and filtered. The filtrate was concentrated under vacuum to give a residue, which was purified by flash column chromatography (PE/EA 8:1 to 6:1) to afford **10** as a white solid (896.2 mg, 87%). $[\alpha]_D^{25}$= -60.63 (*c* 0.16 $CHCl_3$) ^1H NMR (400 MHz, $CDCl_3$) δ 8.10 (d, *J* = 8.3 Hz, 2H), 7.98 (d, *J* = 7.0 Hz, 2H), 7.86 (d, *J* = 7.3 Hz, 2H), 7.71 (d, *J* = 7.7 Hz, 2H), 7.65 (d, *J* = 6.3 Hz, 2H), 7.60 – 7.48 (m, 4H), 7.47 – 7.18 (m, 40H), 7.18 – 7.07 (m, 8H), 7.03 (t, *J* = 7.4 Hz, 1H), 6.94 (t, *J* = 7.5 Hz, 2H), 6.36 (t, *J* = 10.0 Hz, 1H), 5.84 – 5.72 (m, 2H), 5.58 (d, *J* = 2.7 Hz, 1H), 5.36 (s, 1H), 5.21 – 5.12 (m, 3H), 5.08 (d, *J* = 10.7 Hz, 1H), 4.89 (s, 1H), 4.75 – 4.66 (m, 3H), 4.65 – 4.59 (m, 2H), 4.59 – 4.49 (m,

3H), 4.46 (d, J = 8.2 Hz, 2H), 4.41 (dd, J = 9.0, 3.4 Hz, 1H), 4.32 (d, J = 10.7 Hz, 1H), 4.24 – 4.10 (m, 3H), 3.93 – 3.83 (m, 3H), 3.79 – 3.66 (m, 6H), 3.56 – 3.52 (m, 1H), 3.40 (dd, J = 12.0, 2.1 Hz, 1H), 3.26 – 3.09 (m, 3H), 1.51 – 1.37 (m, 4H), 1.22 – 1.12 (m, 2H), 1.02 (s, 9H). ^{13}C NMR (151 MHz, CDCl3) δ 165.9, 165.7, 165.6, 165.0, 138.7, 138.61, 138.4, 138.3, 135.8, 135.7, 133.5, 133.3, 133.2, 133.13, 133.06, 132.95, 130.2, 130.1, 130.03, 129.95, 129.93, 129.88, 129.64, 129.59, 129.5, 128.9, 128.8, 128.7, 128.64, 128.59, 128.55, 128.49, 128.46, 128.4, 128.0, 127.93, 127.88, 127.86, 127.84, 127.77, 127.7, 127.6, 127.6, 127.4, 99.5, 98.7, 79.4, 79.0, 76.0, 75.2, 74.9, 74.8, 73.7, 73.4, 72.3, 72.2, 72.04, 71.99, 71.9, 71.3, 70.8, 69.8, 69.3, 67.7, 67.4, 65.6, 61.4, 50.7, 50.4, 47.3, 46.3, 30.3, 29.9, 29.4, 28.2, 27.7, 26.9, 26.8, 23.6, 19.4. HRMS (ESI) calcd for $C_{117}H_{119}O_{22}NSiNa$ $[M+Na]^+$ 1940.7885 found 1940.7889.

Fully protected trisaccharide 12

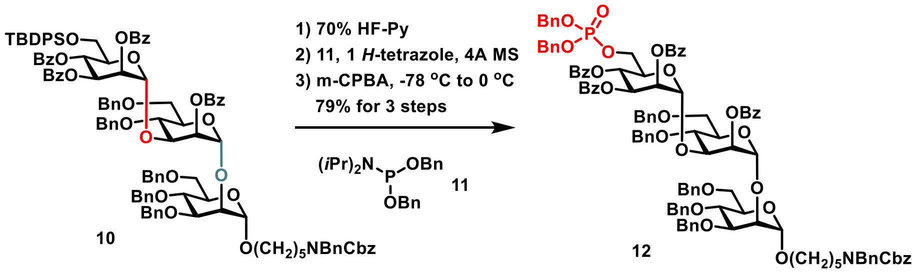

To a solution of **10** (860 mg, 0.45 mmol) in THF (4.5 mL) was added 70% HF·Py (1.3 mL, 9.0 mmol) at 0 °C. The mixture was stirred at room temperature for 24 h and quenched with Et_3N (2.0 mL), then diluted with EtOAc (10.0 mL) and washed with saturated aqueous $NaHCO_3$ (10.0 mL × 3) and brine (30.0 mL) successively. The organic phase was dried with Na_2SO_4 and concentrated in vacuo. The resulting residue was purified by silica gel column chromatography (PE/EA 4:1) to afford the intermediate as a white foam (660 mg, 88%). To a solution of the above intermediate (660 mg, 0.39 mmol) and 1H-tetrazole (192 mg, 2.75 mmol) in DCM (7.9 mL) and 4 Å MS was added dibenzyl N,N-diisopropylphosphoramidite **11** (0.5 mL, 1.96 mmol) dropwise over 5 minutes. After 16 h, the reaction mixture was cooled to −78 °C, and m-CPBA (271 mg of 85% m-CPBA, 1.57 mmol) was added as a solid. The reaction

mixture was allowed to warm to 0 °C. After 4 h, saturated $Na_2S_2O_3$ was added, and the organic layer was removed, washed with saturated $NaHCO_3$, dried with Na_2SO_4, and concentrated under reduced pressure to give a residue, which was purified by flash column chromatography (PE/EA 2:1 to 1.5:1) to afford **12** (684.5 mg, 90%) as a white foam. $[\alpha]_D^{25}$= -29.33 (*c* 0.15 $CHCl_3$) ^{1}H NMR (400 MHz, $CDCl_3$) δ 8.17 (d, 2H), 8.01 (d, *J* = 7.6 Hz, 2H), 7.81 (d, *J* = 6.8 Hz, 2H), 7.69 (d, *J* = 7.3 Hz, 2H), 7.63 (d, *J* = 7.3 Hz, 1H), 7.52 – 7.42 (m, 4H), 7.42 – 7.03 (m, 52H), 6.10 (t, *J* = 9.7 Hz, 1H), 5.82 – 5.72 (m, 2H), 5.69 (t, *J* = 2.5 Hz, 1H), 5.25 (s, 1H), 5.21 (s, 1H), 5.17 (s, 1H), 5.15 (s, 1H), 5.08 – 5.00 (m, 1H), 4.99 – 4.84 (m, 5H), 4.81 (d, *J* = 10.8 Hz, 1H), 4.74 – 4.65 (m, 4H), 4.60 – 4.44 (m, 6H), 4.40 (dd, *J* = 9.2, 3.3 Hz, 1H), 4.28 – 4.19 (m, 3H), 4.13 – 4.07 (m, 1H), 4.01 – 3.87 (m, 4H), 3.86 – 3.75 (m, 2H), 3.75 – 3.66 (m, 2H), 3.61 – 3.51 (m, 1H), 3.30 – 3.11 (m, 3H), 1.57 – 1.37 (m, 4H), 1.29 – 1.09 (m, 3H). ^{13}C NMR (101 MHz, $CDCl_3$) δ 165.8, 165.6, 165.1, 164.7, 138.52, 138.45, 138.3, 138.02, 137.96, 136.04, 135.97, 135.93, 135.86, 133.41, 133.38, 133.2, 133.1, 130.0, 129.9, 129.8, 129.7, 129.3, 129.2, 128.8, 128.74, 128.6, 128.5, 128.44, 128.40, 128.35, 128.31, 128.29, 128.26, 128.2, 128.1, 128.01, 127.95, 127.9, 127.80, 127.78, 127.7, 127.6, 127.5, 127.4, 99.7, 99.4, 98.6, 79.5, 79.4, 76.5, 75.8, 75.2, 74.8, 74.6, 73.6, 73.2, 72.3, 72.2, 71.90, 71.87, 70.6, 70.14, 70.06, 69.5, 69.3, 69.25, 69.2, 69.1, 67.5, 67.2, 65.4, 65.0, 50.5, 50.2, 47.1, 46.2, 29.2, 28.0, 27.5, 23.4. HRMS (ESI) calcd for $C_{115}H_{114}O_{25}NPNa$ $[M+Na]^+$ 1962.7310 found 1962.7315.

Trisaccharide 1

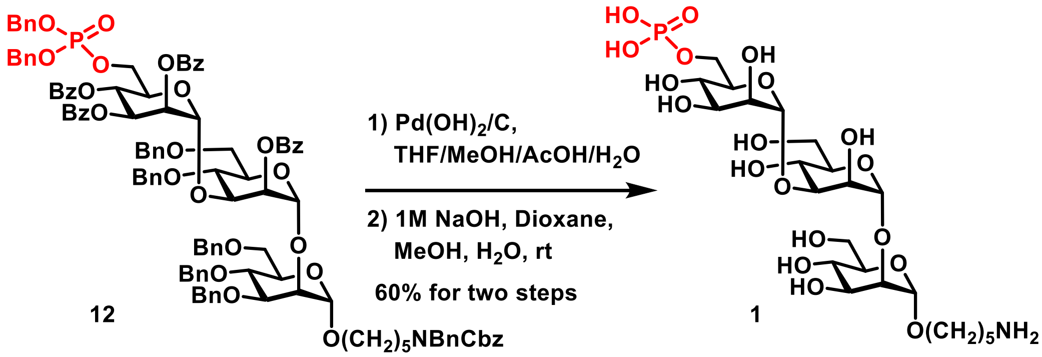

A mixture of **12** (100 mg, 0.052 mmol) and $Pd(OH)_2$/C (358.9 mg, 20%) in THF/MeOH/H_2O/HOAc (1.5 mL/2.0 mL/0.5 mL/0.2 mL) was stirred under an atmosphere of H_2 at room temperature for 2 days. The reaction mixture was filtered and concentrated in vacuo to afford the intermediate. To a solution of above intermediate in dioxane/MeOH (1.4 mL/0.5 mL) was added 1 M NaOH (2.0 mL). The reaction was stirred for 2 d at room temperature. Then the reaction was neutralized to pH 7 by acetic acid, then filtered and concentrated. The residue was purified by column chromatography on Sephadex TM LH-20 (MeOH/H_2O 1:1) to afford deprotected product **1** (22.5 mg, 60% for 2 steps). $[\alpha]_D^{25}$= +79.91 (*c* 1.09 H_2O) ^1H NMR (400 MHz, D_2O) δ 5.03 (d, *J* = 1.8 Hz, 1H), 5.00 (d, *J* = 1.8 Hz, 1H), 4.91 (d, *J* = 1.9 Hz, 1H), 4.19 (dd, *J* = 3.3, 1.9 Hz, 1H), 4.15 – 4.06 (m, 1H), 4.02 – 3.90 (m, 2H), 3.89 – 3.84 (m, 2H), 3.84 – 3.81 (m, 2H), 3.81 – 3.75 (m, 3H), 3.75 – 3.71 (m, 1H), 3.71 – 3.67 (m, 1H), 3.67 – 3.63 (m, 3H), 3.63 – 3.59 (m, 2H), 3.56 – 3.50 (m, 1H), 3.50 – 3.43 (m, 1H), 2.91 (t, *J* = 7.6 Hz, 2H), 1.67 – 1.49 (m, 5H), 1.44 – 1.28 (m, 2H). ^{13}C NMR (151 MHz, D_2O) δ 102.6, 102.3, 98.0, 78.84, 78.78, 73.4, 72.9, 72.3, 70.4, 70.2, 70.0, 69.4, 67.5, 66.8, 66.5, 66.1, 64.4, 61.1, 61.0, 39.4, 27.9, 26.5, 22.4. HRMS (ESI) calcd for $C_{23}H_{44}NO_{19}PH$ $[M+H]^+$ 670.2318 found 670.2322.

4. One-pot orthogonal synthesis of PI-88 glycan motif 2

***N*-Benzyl-*N*-benzyloxycarbonyl-5-aminopentyl 6-*O*-*tert*-butyldiphenylsilyl-2,3,4,-tri-*O*-benzoyl-α-D-Mannopyranosyl-(1→3)-2-*O*-benzoyl-4,6-di-*O*-benzyl-α-D-mannopyranosyl-(1→3)-2-*O*-benzoyl-4,6-di-*O*-benzyl-α-D-mannopyranosyl-(1→2)-3,4,6-tri-*O*-benzyl-α-D-Mannopyranoside (13)**

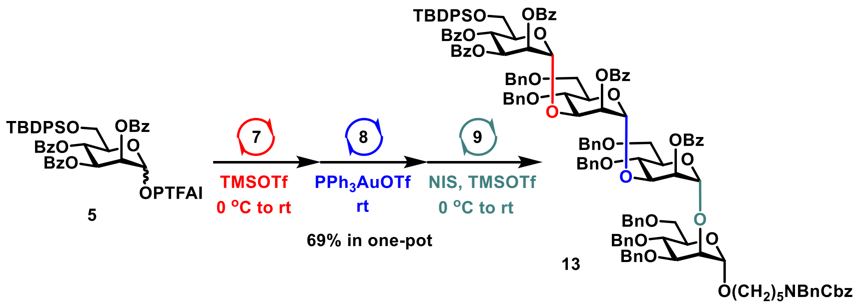

A suspension of donor **5** (474.7 mg, 0.53 mmol), ABz acceptor **7** (305.7 mg, 0.48 mmol), and activated 3Å MS (1.4 g) in dry DCM (4.0 mL) was stirred at room temperature for 15 min and then cooled to 0 °C. A solution of TMSOTf in DCM (0.5 mL, 37 μL TMSOTf in 1.0 mL DCM) was slowly added to the mixture. After being stirred at 0 °C to room temperature for another 2 h, PVB acceptor **8** (294 mg, 0.44 mmol) and a freshly prepared solution of PPh_3AuOTf (0.5 mL, 0.42 mmol) were added successively. After being stirred at room temperature overnight, the mixture was cooled to 0 °C and acceptor **9** (300 mg, 0.39 mmol), NIS (130 mg, 0.58 mmol) and a solution of TMSOTf in DCM (0.5 mL, 31 μL TMSOTf in 1.0 mL DCM) were added successively. The reaction mixture was allowed to warm to room temperature and stirred for 4 h. Then it was quenched with Et_3N (1.0 mL) and filtered. The filtrate was concentrated under vacuum to give a residue, which was purified by flash column chromatography (PE/EA 4:1 to 2:1) to afford **13** (772 mg, 69% in one-pot) as a white foam. $[\alpha]_D^{25}$= -63.33 (*c* 0.15 $CHCl_3$) ^{1}H NMR (400 MHz, $CDCl_3$) δ 8.13 (d, *J* = 8.8 Hz, 2H), 8.05 (d, *J* = 7.8 Hz, 2H), 7.97 (d, *J* = 7.3 Hz, 2H), 7.82 (d, *J* = 7.6 Hz, 2H), 7.77 (d, *J* = 7.8 Hz, 2H), 7.68 – 7.61 (m, 2H), 7.57 – 7.45 (m, 4H), 7.42 – 7.34 (m, 13H), 7.34 – 7.30 (m, 5H), 7.29 – 7.22 (m, 21H), 7.22 – 7.16 (m, 12H), 7.16 – 7.11 (m, 5H), 7.09 – 6.91 (m, 8H), 6.29 (t, *J* = 10.3 Hz, 1H), 5.75 – 5.70 (m, 1H), 5.70 –

5.62 (m, 2H), 5.55 (s, 1H), 5.34 (s, 1H), 5.26 (s, 1H), 5.24 (s, 1H), 5.16 (s, 1H), 5.14 (s, 1H), 4.93 (d, J = 11.0 Hz, 1H), 4.89 – 4.82 (m, 2H), 4.72 – 4.56 (m, 6H), 4.56 – 4.42 (m, 6H), 4.41 – 4.36 (m, 1H), 4.35 – 4.28 (m, 3H), 4.22 (d, J = 10.8 Hz, 1H), 4.06 – 4.00 (m, 1H), 3.96 – 3.85 (m, 3H), 3.85 – 3.74 (m, 2H), 3.73 – 3.60 (m, 6H), 3.57 – 3.42 (m, 2H), 3.26 – 3.10 (m, 4H), 1.52 – 1.34 (m, 4H), 1.26 – 1.10 (m, 2H), 0.99 (s, 9H). ^{13}C NMR (101 MHz, $CDCl_3$) δ 165.8, 165.5, 165.3, 164.8, 138.53, 138.48, 138.4, 138.3, 138.0, 137.8, 135.8, 135.6, 135.4, 133.2, 133.1, 133.0, 132.8, 130.0, 129.94, 129.88, 129.80, 129.75, 129.7, 129.5, 129.4, 129.3, 128.63, 128.55, 128.48, 128.46, 128.4, 128.38, 128.3, 128.29, 128.25, 128.18, 128.17, 128.1, 128.0, 127.93, 127.89, 127.8, 127.6, 127.6, 127.6, 127.5, 127.49, 127.43, 127.39, 127.34, 99.8, 99.5, 99.4, 98.6, 79.4, 78.5, 77.8, 76.5, 75.3, 75.2, 74.86, 74.3, 73.5, 73.4, 73.2, 72.7, 72.5, 72.2, 72.0, 71.9, 71.8, 71.6, 70.9, 70.6, 69.4, 69.2, 68.4, 67.5, 67.2, 65.6, 61.0, 50.2, 47.1, 29.2, 26.7, 26.5, 23.4, 19.1. MS (Maldi-TOF) calcd for $C_{144}H_{145}NO_{28}SiNa$ $[M+Na]^+$ 2386.9615, found 2385.9613.

Fully protected tetrasaccharide 14

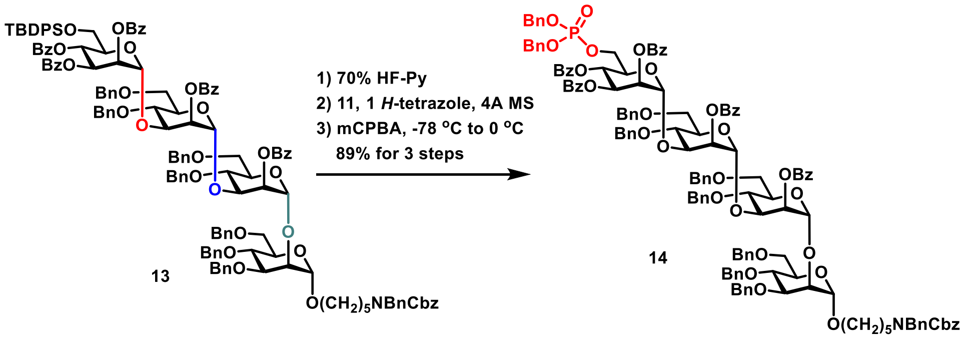

To a solution of **13** (627 mg, 0.28 mmol) in THF (3.0 mL) was added 70% HF·Py (0.7 mL, 5.54 mmol) at 0 °C. The mixture was stirred at room temperature for 24 h and quenched with Et_3N (2.0 mL), then it was diluted with EtOAc (10.0 mL) and washed with saturated aqueous $NaHCO_3$ (10.0 mL × 3) and brine (30 mL) successively. The organic phase was dried with Na_2SO_4 and concentrated in vacuo. The resulting residue was purified by silica gel column chromatography (PE/EA 4:1) to afford the intermediate as a white foam (540 mg, 96%). To a mixture of the above

intermediate (540 mg, 0.27 mmol), 1*H*-tetrazole (131 mg, 1.87 mmol) and 4 Å MS in dry DCM (5.3 mL) was added dibenzyl *N*,*N*-diisopropylphosphoramidite (**11**, 360 μL, 1.33 mmol) dropwise over 5 minutes. After 16 h, the reaction mixture was cooled to −78 °C, and *m*-CPBA (184.2 mg of 85% *m*-CPBA, 1.07 mmol) was added as a solid. The reaction mixture was allowed to warm to 0 °C and after 4 h, saturated $Na_2S_2O_3$ was added. The organic layer was washed with saturated $NaHCO_3$, dried with Na_2SO_4, and concentrated under reduced pressure to give a residue, which was purified by flash column chromatography (PE/EA 2:1 to 1.5:1) to afford **14** (596 mg, 93%) as a white foam. $[\alpha]_D^{25}$= -41.60 (*c* 0.75 $CHCl_3$) ^{1}H NMR (400 MHz, $CDCl_3$) δ 8.17 – 8.04 (m, 3H), 7.95 (d, *J* = 7.8 Hz, 2H), 7.80 – 7.70 (m, 3H), 7.60 (t, *J* = 7.4 Hz, 1H), 7.52 – 7.43 (m, 3H), 7.43 – 7.31 (m, 16H), 7.31 – 7.21 (m, 26H), 7.21 – 7.17 (m, 9H), 7.17 – 7.14 (m, 6H), 7.14 – 7.08 (m, 4H), 7.08 – 7.02 (m, 2H), 6.09 – 5.95 (m, 1H), 5.72 – 5.60 (m, 4H), 5.36 (s, 1H), 5.22 (d, *J* = 2.0 Hz, 1H), 5.19 (d, *J* = 2.0 Hz, 1H), 5.16 (s, 1H), 5.14 (s, 1H), 5.12 – 5.00 (m, 3H), 4.99 – 4.79 (m, 8H), 4.72 – 4.58 (m, 5H), 4.55 (dd, *J* = 11.1, 4.6 Hz, 2H), 4.52 – 4.41 (m, 5H), 4.38 – 4.26 (m, 3H), 4.24 – 4.14 (m, 2H), 4.08 – 3.98 (m, 2H), 3.97 – 3.90 (m, 2H), 3.90 – 3.85 (m, 1H), 3.85 – 3.75 (m, 3H), 3.74 – 3.66 (m, 4H), 3.66 – 3.57 (m, 2H), 3.56 – 3.47 (m, 1H), 3.24 – 3.10 (m, 3H), 1.53 – 1.34 (m, 4H), 1.23 – 1.10 (m, 2H). ^{13}C NMR (101 MHz, $CDCl_3$) δ 165.8, 165.74, 165.37, 165.0, 164.8, 138.51, 138.47, 138.4, 138.3, 138.2, 137.9, 137.8, 136.0, 135.9, 135.8, 133.4, 133.3, 133.2, 133.0, 132.97, 130.08, 130.03, 129.9, 129.82, 129.77, 129.7, 129.63, 129.57, 129.27, 129.26, 129.2, 128.73, 128.66, 128.61, 128.55, 128.51, 128.46, 128.44, 128.41, 128.37, 128.35, 128.31, 128.25, 128.23, 128.19, 128.2, 128.11, 128.06, 128.00, 127.95, 127.9, 127.84, 127.82, 127.76, 127.7, 127.64, 127.56, 127.5, 127.44, 127.37, 127.35, 127.0, 99.5, 99.4, 98.6, 79.4, 78.5, 77.9, 76.54, 75.48, 75.3, 75.2, 74.9, 74.8, 74.3, 73.4, 73.2, 72.5, 72.1, 72.0, 71.9, 71.8, 70.3, 70.0, 69.4, 69.3, 69.24, 69.22, 69.2, 68.3, 67.5, 67.4, 67.3, 67.2, 65.5, 65.3, 65.0, 45.6, 29.2, 23.4, 8.5. MS (Maldi-TOF) calcd for $C_{142}H_{140}NO_{31}PNa$ $[M+Na]^+$ 2408.9030, found 2408.9034.

Tetrasaccharide 2

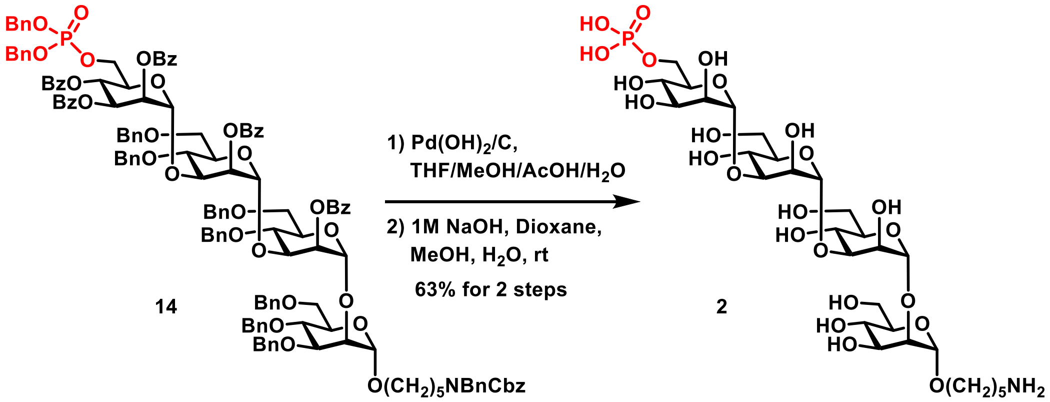

A mixture of **14** (100 mg, 0.04 mmol) and $Pd(OH)_2/C$ (338.2 mg, 20%) in THF/MeOH/H_2O/HOAc (1.5 mL/2.0 mL/0.5 mL/0.2 mL) was stirred under an atmosphere of H_2 at room temperature for 2 days. The reaction mixture was filtered and concentrated in vacuo to afford the intermediate. To a solution of above intermediate in dioxane/MeOH (1.4 mL/0.5 mL) was added 1 M NaOH (2.0 mL). The reaction mixture was stirred for 2 d at room temperature. Then, the reaction mixture was neutralized to pH 7 by acetic acid, then filtered and concentrated. The residue was purified by column chromatography on Sephadex TM LH-20 (MeOH/H_2O 1:1) to afford deprotected product **2** (23 mg, 63% for 2 steps). $[\alpha]_D^{25}$= +94.82 (*c* 1.12 H_2O). ^{1}H NMR (400 MHz, D_2O) δ 5.04 (s, 1H), 5.02 (s, 1H), 5.01 (s, 1H), 4.92 (s, 1H), 4.25 (q, *J* = 1.8 Hz, 1H), 4.16 – 4.05 (m, 2H), 4.00 (dd, *J* = 3.4, 1.7 Hz, 1H), 3.94 – 3.77 (m, 10H), 3.77 – 3.72 (m, 1H), 3.72 – 3.56 (m, 9H), 3.56 – 3.43 (m, 2H), 3.01 – 2.86 (m, 2H), 1.68 – 1.50 (m, 4H), 1.47 – 1.29 (m, 2H). ^{13}C NMR (151 MHz, D_2O) δ 102.8, 102.3, 102.2, 98.0, 79.2, 78.6, 78.1, 73.5, 73.3, 72.8, 72.43, 72.39, 70.3, 70.2, 70.0, 69.6, 69.38, 67.42, 67.37, 66.9, 66.6, 65.94, 65.87, 64.23, 64.20, 61.03, 60.99, 60.95, 39.3, 27.9, 26.4, 22.4. HRMS (ESI) calcd for $C_{29}H_{54}NO_{24}P$ $[M-H]^-$, 830.2701, found 830.2707.

5. One-pot orthogonal synthesis of PI-88 glycan motif 3

***N*-Benzyl-*N*-benzyloxycarbonyl-5-aminopentyl 2-*O*-benzoyl-3-*O*-levulinoyl-4,6-di-*O*-benzyl-α-D-mannopyranosyl-(1→3)-2-*O*-benzoyl-4,6-di-*O*-benzyl-α-D-mannopyranosyl-(1→2)-3,4,6-tri-*O*-benzy-α-D-mannopyranoside (15)**

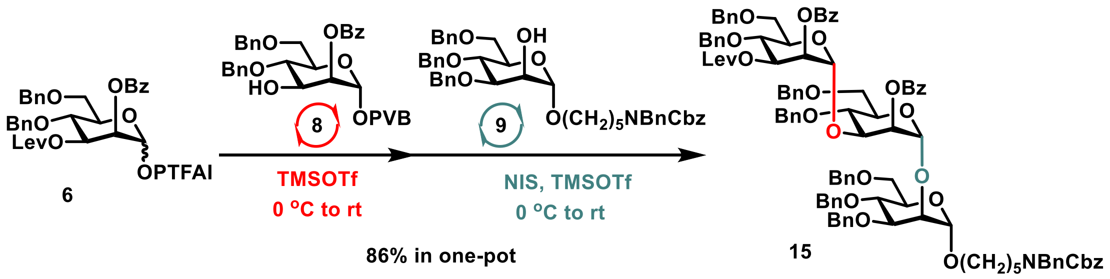

A suspension of donor **6** (482 mg, 0.66 mmol), PVB acceptor **8** (367 mg, 0.55 mmol) and activated 3Å MS (487mg) in dry DCM (6.6mL) was stirred at room temperature for 15 min and then cooled to 0 °C. A solution of TMSOTf (0.5 mL, 48 µL TMSOTf in 1.0 mL of DCM) was added to the mixture dropwise. After being stirred at 0 °C to room temperature for another 2 h, the mixture was cooled to 0 °C and acceptor **9** (378 mg, 0.50 mmol), NIS (246 mg, 1.09 mmol), and a solution of TMSOTf (0.5 mL, 40 µL TMSOTf in 1.0 mL DCM) were added successively. The reaction mixture was allowed to warm to room temperature and stirred for 4 h. Then it was quenched with Et_3N (1 mL) and filtered. The filtrate was concentrated under vacuum to give a residue, which was purified by flash column chromatography (PE/EA 6:1 to 4:1) to afford **15** (749.3 mg, 86%) as a white foam. $[\alpha]_D^{25}$= -5.61 (*c* 0.41 $CHCl_3$) ^{1}H NMR (400 MHz, $CDCl_3$) δ 8.07 (d, *J* = 7.4 Hz, 2H), 7.99 (d, *J* = 7.4 Hz, 2H), 7.60 – 7.51 (m, 2H), 7.42 – 7.18 (m, 40H), 7.17 – 7.10 (m, 6H), 7.07 – 7.01 (m, 3H), 5.64 (t, *J* = 2.6 Hz, 1H), 5.61 (t, *J* = 2.5 Hz, 1H), 5.43 (dd, *J* = 9.9, 3.1 Hz, 1H), 5.23 – 5.10 (m, 4H), 4.96 (d, *J* = 10.7 Hz, 1H), 4.89 – 4.81 (m, 2H), 4.72 – 4.57 (m, 5H), 4.60 – 4.51 (m, 2H), 4.54 – 4.40 (m, 6H), 4.38 – 4.28 (m, 2H), 4.27 – 4.07 (m, 3H), 4.09 – 4.01 (m, 1H), 3.93 (d, *J* = 9.4 Hz, 1H), 3.91 – 3.82 (m, 3H), 3.82 – 3.63 (m, 5H), 3.62 – 3.47 (m, 3H), 3.26 – 3.10 (m, 3H), 2.72 – 2.50 (m, 2H), 2.50 – 2.30 (m, 2H), 2.06 (s, 3H), 1.52 – 1.35 (m, 4H), 1.24 – 1.09 (m, 2H). ^{13}C NMR (151 MHz, $CDCl_3$) δ 206.5, 171.9, 165.9, 165.4, 156.8, 156.3, 138.6, 138.6, 138.4, 138.4, 138.2, 138.0, 133.4, 133.4,

130.0, 129.9, 129.8, 128.7, 128.7, 128.6, 128.55, 128.50, 128.43, 128.39, 128.32, 128.28, 128.1, 128.0, 127.9, 127.8, 127.74, 127.67, 127.60, 127.56, 127.54, 127.52, 127.50, 127.4, 127.3, 99.9, 99.4, 98.6, 79.4, 76.8, 75.8, 75.4, 74.9, 74.5, 74.4, 73.6, 73.5, 73.3, 72.6, 72.5, 72.4, 72.3, 72.2, 72.0, 71.9, 70.4, 69.5, 69.2, 68.3, 67.6, 67.3, 50.6, 50.3, 47.2, 46.2, 38.0, 32.1, 31.6, 30.4, 30.2, 29.9, 29.8, 29.6, 29.5, 29.3, 28.14, 28.07, 27.6, 27.1, 23.5, 22.8, 19.9, 14.3, 1.2, 0.1. HRMS (ESI) calcd for $C_{106}H_{111}NO_{22}NH_4$ $[M+NH_4]^+$, 1767.7936, found 1767.7938.

N-(Benzyl)benzyloxycarbonyl-5-amino-pentyl-2-O-benzoyl-4,6-di-O-benzyl-α-D-mannopyranosyl-(1→3)-2-O-benzoyl-4,6-di-O-benzyl-α-D-mannopyranosyl-(1→2)-3,4,6-tri-O-benzy-α-D-Mannopyranoside (16)

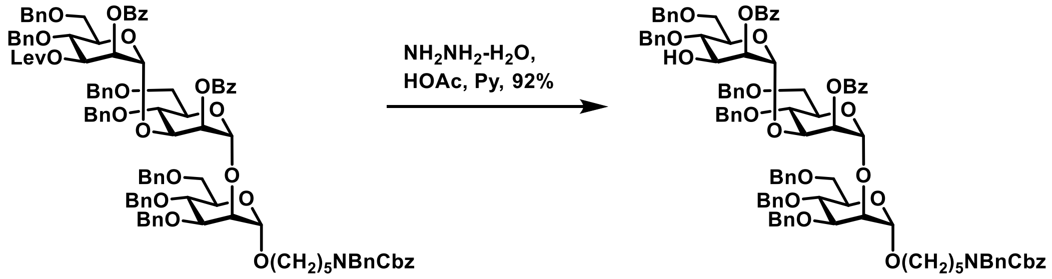

Compound **15** (315.2 mg, 0.18 mmol) was dissolved in pyridine/AcOH (1.2 mL/0.6 mL), and NH_2NH_2•H_2O (32 μL, 0.53 mmol) was added slowly. After stirring overnight at room temperature, the reaction was quenched with acetone. The mixture was concentrated under vacuum, then the residue was diluted with EtOAc, and washed with 4 M HCl, saturated $NaHCO_3$ and brine. The organic layer was dried over anhydrous Na_2SO_4, filtered and concentrated in vacuo. The residue was purified by flash column chromatography (PE/EA 4:1) to afford **16** (268 mg, 92%). $[\alpha]_D25$= -11.90 (c 0.21 $HCCl_3$) 1H NMR (400 MHz, CDCl3) δ 8.11 – 8.03 (m, 2H), 8.00 – 7.94 (m, 2H), 7.59 (t, J = 7.5 Hz, 1H), 7.56 – 7.51 (m, 1H), 7.38 (q, J = 8.2 Hz, 5H), 7.35 – 7.30 (m, 3H), 7.30 – 7.17 (m, 25H), 7.17 – 7.03 (m, 6H), 5.63 – 5.57 (m, 1H), 5.41 (dd, J = 3.1, 1.8 Hz, 1H), 5.26 (d, J = 1.8 Hz, 1H), 5.20 – 5.12 (m, 2H), 4.91 – 4.77 (m, 3H), 4.64 (dt, J = 11.4, 3.9 Hz, 4H), 4.57 (td, J = 10.3, 7.1 Hz, 4H), 4.51 – 4.42 (m, 5H), 4.39 (dd, J = 9.1, 3.2 Hz, 1H), 4.30 (d, J = 11.9 Hz, 1H), 4.18 – 4.09 (m, 2H),

4.09 – 4.01 (m, 2H), 3.90 (s, 2H), 3.79 (dd, J = 19.4, 10.5 Hz, 2H), 3.74 – 3.66 (m, 4H), 3.66 – 3.57 (m, 2H), 3.26 – 3.07 (m, 3H), 2.00 (d, J = 4.4 Hz, 1H), 1.43 (d, J = 21.8 Hz, 4H), 1.18 (d, J = 23.8 Hz, 2H). 13C NMR (151 MHz, CDCl3) δ 165.80, 165.55, 156.68, 138.47, 138.41, 138.36, 138.28, 137.86, 133.23, 133.16, 129.89, 129.84, 129.80, 129.64, 128.52, 128.51, 128.40, 128.37, 128.34, 128.33, 128.28, 128.23, 128.21, 128.19, 128.18, 128.08, 128.01, 127.99, 127.82, 127.79, 127.73, 127.66, 127.61, 127.54, 127.51, 127.49, 127.43, 127.36, 127.31, 127.16, 99.43, 98.50, 79.33, 75.29, 74.98, 74.86, 74.82, 74.04, 73.34, 73.17, 72.60, 72.34, 72.23, 72.01, 71.91, 71.72, 70.00, 69.35, 69.06, 68.45, 67.47, 67.12, 50.44, 50.13, 47.05, 46.08, 29.69, 29.16, 27.91, 27.47, 23.36, MS (ESI) calcd for $C_{101}H_{105}NO_{20}Na$ [M+Na]+, 1674.7122, found 1674.7126.

***N*-Benzyl-*N*-benzyloxycarbonyl-5-aminopentyl 6-*O*-*tert*-butyldiphenylsily-2,3,4,-tri-*O*-benzoyl-α-D-mannopyranosyl-(1→3)-2-*O*-benzoyl-4, 6-di-*O*-benzyl-α-D-mannopyranosyl-(1→3)-2-*O*-benzoyl-4,6-di-*O*-benzyl-α-D-mannopyranosyl-(1→3)-2-*O*-benzoyl-4,6-di-*O*-benzyl-α-D-Mannopyranosyl-(1→2)-3,4,6-tri-*O*-benzyl-α-D-mannopyranoside (17)**

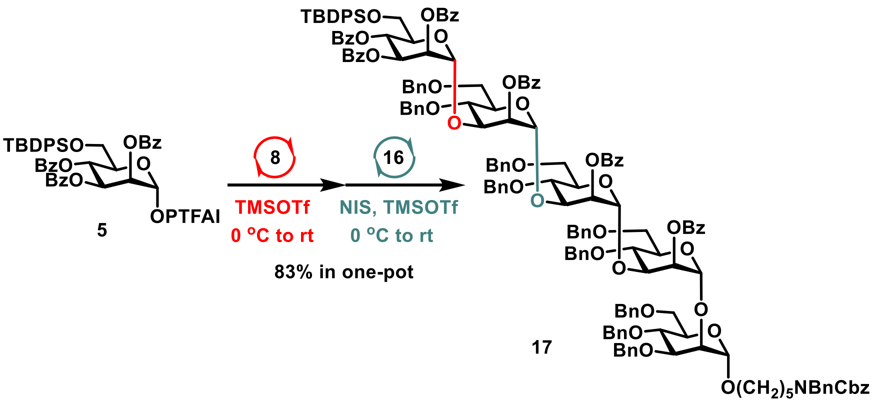

A suspension of donor **5** (327 mg, 0.36 mmol), PVB acceptor **8** (223.2 mg, 0.33 mmol) and activated 3Å MS (1.1 g) in dry DCM (4.0mL) was stirred at room temperature for 15 min and then cooled to 0 °C. A solution of TMSOTf (0.5 mL, 27 μL TMSOTf in 1.0 mL of DCM) was added to the mixture dropwise. After being stirred at 0 °C to

room temperature for another 2 h, the mixture was cooled to 0 °C and acceptor **16** (500 mg, 0.30 mmol), NIS (112.3 mg, 0.50 mmol), a solution of TMSOTf (0.5 mL, 24 µL TMSOTf in 1.0 mL DCM) were added successively. The reaction mixture was allowed to warm to room temperature and stirred for 4 h. Then the reaction was quenched with Et_3N (1 mL) and filtered. The filtrate was concentrated under vacuum to give a residue, which was purified by flash column chromatography (PE/EA 4:1 to 2:1) to afford **17** (706 mg, 83%) as a white solid. $[\alpha]_D^{25}$= -45.38 (*c* 0.13 $HCCl_3$) ^1H NMR (400 MHz, $CDCl_3$) δ 8.13 – 8.02 (m, 6H), 7.96 – 7.90 (m, 2H), 7.86 – 7.80 (m, 2H), 7.80 – 7.75 (m, 2H), 7.68 – 7.61 (m, 2H), 7.54 (m, 3H), 7.46 (dt, *J* = 9.7, 6.2 Hz, 2H), 7.42 – 7.33 (m, 12H), 7.33 – 7.29 (m, 6H), 7.26 (m, 20H), 7.18 (m,11H), 7.12 (m,3H), 7.00 – 6.91 (m, 5H), 6.91 – 6.85 (m, 2H), 6.28 (t, *J* = 10.0 Hz, 1H), 5.73 – 5.57 (m, 4H), 5.47 (t, *J* = 2.4 Hz, 1H), 5.40 – 5.35 (m, 1H), 5.23 – 5.12 (m, 6H), 5.00 (d, *J* = 10.6 Hz, 1H), 4.93 – 4.86 (m, 2H), 4.82 (d, *J* = 10.7 Hz, 1H), 4.72 – 4.64 (m, 2H), 4.64 – 4.58 (m, 3H), 4.58 – 4.53 (m, 3H), 4.52 – 4.44 (m, 5H), 4.42 (dd, *J* = 9.2, 3.1 Hz, 1H), 4.35 – 4.28 (m, 2H), 4.28 – 4.22 (m, 3H), 4.22 – 4.13 (m, 3H), 4.09 – 4.00 (m, 2H), 3.92 – 3.81 (m, 5H), 3.76 (t, *J* = 8.9 Hz, 1H), 3.70 (d, *J* = 12.7 Hz, 4H), 3.60 (dt, *J* = 8.2, 2.3 Hz, 1H), 3.55 (t, *J* = 11.0 Hz, 3H), 3.44 (dd, *J* = 11.2, 4.8 Hz, 2H), 3.19 (d, *J* = 12.3 Hz, 5H), 1.52 – 1.36 (m, 5H), 1.23 – 1.12 (m, 1H). ^{13}C NMR (151 MHz, $CDCl_3$) δ 165.8, 165.6, 165.43, 165.35, 164.9, 138.7, 138.6, 138.5, 138.4, 138.2, 138.1, 135.85, 135.80, 135.6, 133.4, 133.2, 133.11, 133.06, 132.7, 130.11, 130.08, 130.0, 129.94, 129.90, 129.87, 129.85, 129.77, 129.6, 129.5, 129.4, 128.69, 128.66, 128.62, 128.58, 128.54, 128.49, 128.45, 128.40, 128.37, 128.34, 128.29, 128.27, 128.2, 128.13, 128.09, 128.03, 127.95, 127.9, 127.8, 127.70, 127.66, 127.6, 127.54, 127.47, 127.4, 127.2, 127.0, 100.0, 99.6, 99.5, 98.8, 98.6, 79.4, 78.7, 77.9, 75.8, 75.6, 75.3, 75.0, 74.9, 74.5, 74.3, 74.2, 73.6, 73.5, 73.4, 73.4, 73.3, 72.8, 72.6, 72.52, 72.48, 72.3, 72.2, 72.1, 71.94, 71.87, 71.7, 71.0, 70.6, 69.5, 69.2, 68.5, 68.4, 67.6, 67.3, 65.6, 60.9, 50.6, 50.3, 47.2, 46.2, 37.6, 37.3, 32.9, 32.1, 31.6, 30.4, 30.2, 30.1, 29.8, 29.5, 29.3, 28.1, 27.6, 27.3, 26.7, 26.6, 26.5, 23.5, 22.8, 19.9, 19.2. MS (Maldi-TOF) m/z: $[M + Na]^+$ Calcd for $C_{171}H_{171}NO_{34}SiNa$ 2833.1344; Found 2833.1342.

Fully protected pentasaccharide 18

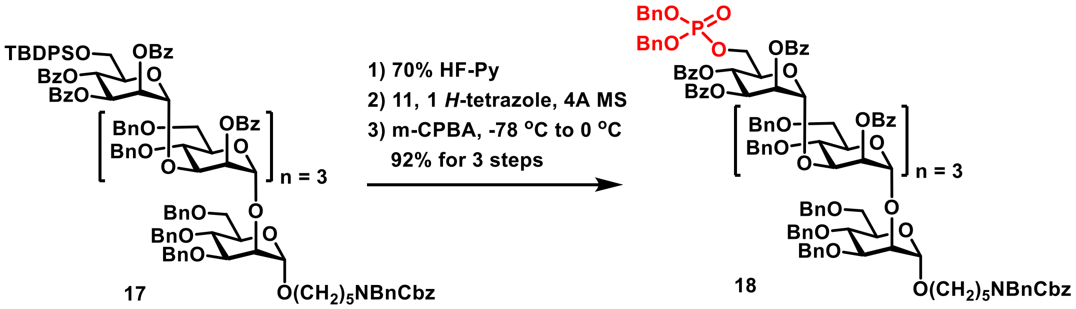

To a solution of **17** (610 mg, 0.22 mmol) in THF (2.1 mL) was added 70% HF·Py (306 µL, 0.59 mmol) at 0 °C. The mixture was stirred at room temperature for 24 h and quenched with Et_3N (2.0 mL). Then it was diluted with EtOAc (10.0 mL) and washed with saturated aqueous $NaHCO_3$ (10.0 mL × 3) and brine (30.0 mL) successively. The organic phase was dried with Na_2SO_4 and concentrated in vacuo. The resulting residue was purified by silica gel column chromatography (PE/EA 4:1) to afford the intermediate as a white foam (540 mg, 97%). To a mixuture of the above intermediate (540 mg, 0.21 mmol), 1*H*-tetrazole (103 mg, 1.47 mmol) and 4 Å MS in dry DCM (4.0 mL) was added dibenzyl *N*,*N*-diisopropylphosphoramidite (**11**, 362 µL, 1.05 mmol) dropwise over 5 minutes. After 16 h, the reaction mixture was cooled to −78 °C, and *m*-CPBA (145 mg of 85% m-CPBA, 0.84 mmol) was added as a solid. The reaction mixture was allowed to warm to 0 °C. After 4 h, saturated $Na_2S_2O_3$ was added, and the organic layer was washed with saturated $NaHCO_3$, dried with Na_2SO_4, and concentrated under reduced pressure. The residue was purified by flash column chromatography (PE/EA 2:1 to 1.5:1) to afford **18** (583 mg, 95%) as a white foam $[\alpha]_D^{25}$= +1.18 (*c* 0.68 $CHCl_3$) ^{1}H NMR (400 MHz, $CDCl_3$) δ 8.09 – 8.00 (m, 6H), 7.95 (d, *J* = 7.6 Hz, 2H), 7.77 (d, *J* = 7.6 Hz, 2H), 7.73 (d, *J* = 6.9 Hz, 2H), 7.60 (d, *J* = 7.5 Hz, 1H), 7.52 – 7.42 (m, 5H), 7.42 – 7.35 (m, 8H), 7.35 – 7.19 (m, 46H), 7.19 – 7.14 (m, 11H), 7.14 – 7.07 (m, 11H), 7.05 – 6.99 (m, 1H), 6.04 (t, *J* = 9.9 Hz, 1H), 5.67 (dd, *J* = 10.0, 3.1 Hz, 1H), 5.64 – 5.57 (m, 4H), 5.36 (s, 1H), 5.26 – 5.19 (m, 2H), 5.19 – 5.10 (m, 3H), 5.03 – 4.89 (m, 4H), 4.89 – 4.78 (m, 5H), 4.71 – 4.61 (m, 3H), 4.59 (s, 1H), 4.57 – 4.39 (m, 11H), 4.38 – 4.12 (m, 9H), 4.07 – 4.02 (m, 1H), 4.01 – 3.95 (m, 1H), 3.92 – 3.78 (m, 6H), 3.76 – 3.64 (m, 5H), 3.61 – 3.48 (m, 4H), 3.41 (d, *J* = 10.8

Hz, 1H), 3.27 – 3.08 (m, 3H), 1.54 – 1.35 (m, 4H), 1.24 – 1.10 (m, 2H). ^{13}C NMR (151 MHz, $CDCl_3$) δ 168.0, 165.8, 165.7, 165.5, 165.1, 164.8, 138.6, 138.5, 138.3, 138.2, 138.0, 136.04, 136.00, 135.95, 135.9, 133.6, 133.43, 133.40, 133.36, 133.3, 133.1, 131.2, 130.3, 130.1, 130.02, 129.97, 129.9, 129.8, 129.7, 129.4, 129.3, 128.74, 128.70, 128.68, 128.66, 128.61, 128.58, 128.51, 128.48, 128.45, 128.42, 128.39, 128.36, 128.28, 128.13, 128.06, 127.96, 127.9, 127.74, 127.70, 127.67, 127.61, 127.57, 127.52, 127.47, 127.4, 127.3, 99.7, 99.5, 99.4, 98.6, 79.4, 78.7, 78.2, 78.0, 76.7, 75.8, 75.4, 75.3, 75.03, 74.99, 74.9, 74.3, 74.2, 73.5, 73.4, 73.3, 72.7, 72.6, 72.5, 72.45, 72.1, 72.0, 71.91, 71.87, 70.4, 70.1, 70.0, 69.5, 69.4, 69.3, 69.3, 69.2, 68.3, 67.6, 67.3, 65.5, 65.0, 50.6, 50.3, 47.2, 46.2, 29.3, 28.0, 27.6, 23.5. HRMS (ESI) calcd for $C_{169}H_{166}NO_{37}PNH_4$ $[M+NH_4]^+$, 2850.1215, found 2850.1204.

Pentasaccharide 3

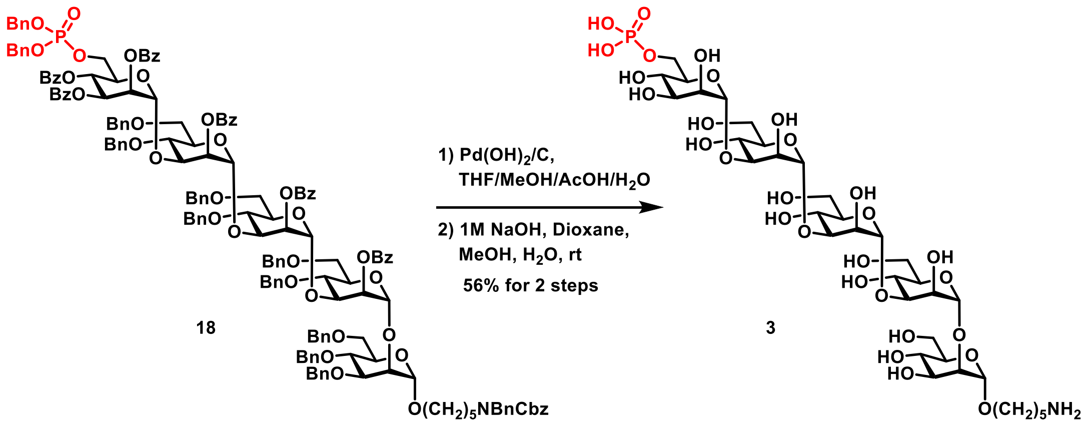

A mixutre of **18** (100 mg, 0.035 mmol) and $Pd(OH)_2/C$ (358.9 mg, 20%) in THF/MeOH/H_2O/HOAc (1.5 mL/2.0 mL/0.5 mL/0.2 mL) was stirred under an atmosphere of H_2 at room temperature for 2 days. The reaction mixture was filtered and concentrated in vacuo to afford the intermediate. To a solution of above intermediate in dioxane/MeOH (1.4 mL/0.5 mL) was added 1 M NaOH (2.0 mL). The reaction mixture was stirred for 2 d at room temperature. Then, the reaction mixture was neutralized to pH 7 by acetic acid, filtered and concentrated. The residue was

purified by column chromatography on Sephadex TM LH-20 (MeOH-H_2O, 1:1) to afford deprotection product **3** (19.8 mg, 56% for 2 steps). $[\alpha]_D^{25}$= +81.67 (*c* 0.060 H_2O) ^{1}H NMR (600 MHz, D_2O) δ 5.02 (s, 2H), 5.00 (s, 1H), 4.99 (s, 1H), 4.91 (s, 1H), 4.25 (t, *J* = 3.5 Hz, 1H), 4.15 – 4.12 (m, 1H), 4.12 – 4.10 (m, 1H), 4.09 (t, *J* = 7.9 Hz, 1H), 4.00 – 3.96 (m, 1H), 3.91 – 3.82 (m, 9H), 3.82 – 3.72 (m, 10H), 3.72 – 3.56 (m, 18H), 3.55 – 3.49 (m, 3H), 3.47 – 3.41 (m, 2H), 2.98 – 2.93 (m, 2H), 2.90 (t, *J* = 7.6 Hz, 1H), 1.66 – 1.50 (m, 4H), 1.39 – 1.31 (m, 2H). ^{13}C NMR (151 MHz, D_2O) δ 102.8, 102.5, 102.2, 102.0, 98.0, 79.3, 78.7, 78.3, 77.8, 73.6, 73.4, 73.4, 72.8, 72.5, 70.2, 70.0, 69.7, 69.5, 69.3, 67.4, 66.9, 66.7, 66.2, 65.8, 64.1, 61.1, 61.0, 60.9, 60.8, 47.4, 47.3, 39.3, 28.3, 27.9, 26.4, 25.3, 22.5, 22.4, 22.3. MS (ESI) calcd for $C_{35}H_{64}NO_{29}P$ $[M-H]^-$, 992.3229, found 992.3224.

6. One-pot orthogonal synthesis of PI-88 glycan motif 4

***N*-Benzyl-*N*-benzyloxycarbonyl-5-aminopentyl 6-*O*-*tert*-butyldiphenylsily-2,3,4,-tri-*O*-benzoyl-α-D-mannopyranosyl-(1→3)-2-*O*-benzoyl-4,6-di-*O*-benzyl-α-D-mannopyranosyl-(1→3)-2-*O*-benzoyl-4,6-di-*O*-benzyl-α-D-mannopyranosyl-(1→3)-2-*O*-benzoyl-4,6-di-*O*-benzyl-α-D-mannopyranosyl-(1→3)-2-*O*-benzoyl-4,6-di-*O*-benzyl-α-D-mannopyranosyl-(1→2)-3,4,6-tri-*O*-benzyl-α-D-mannopyranoside (19)**

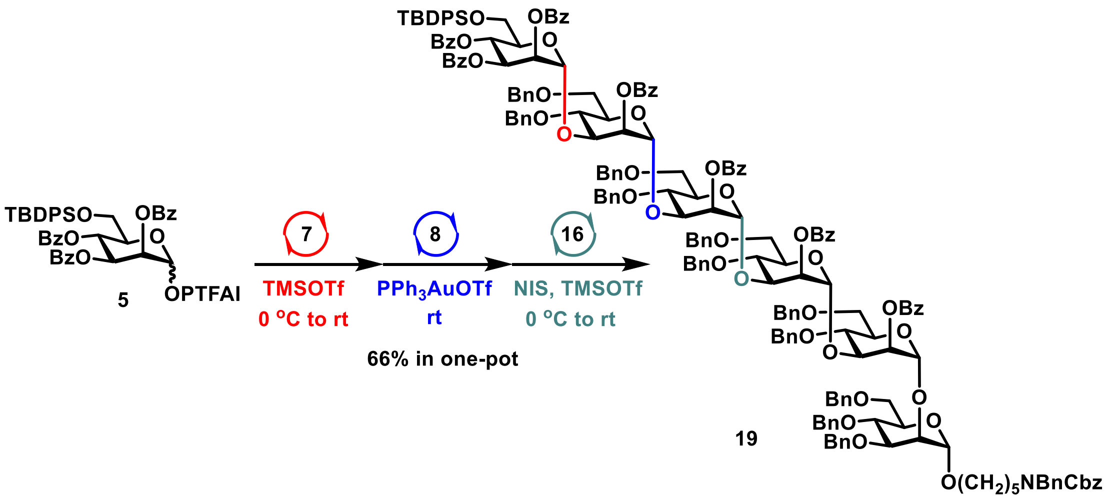

A suspension of donor **5** (82.7 mg, 0.092 mmol), ABz acceptor **7** (53.3 mg, 0.084 mmol) and activated 3Å MS (300 mg) in dry DCM (2.0 mL) was stirred at room temperature for 15 min and then cooled to 0 °C. A solution of TMSOTf (0.1 mL, 33 μL TMSOTf in 1.0 mL DCM) was added to the mixture dropwise. After being stirred at 0 °C to room temperature for another 2 h, acceptor **8** (51.3 mg, 0.076 mmol) and a freshly prepared solution of PPh_3AuOTf in DCM (0.5 mL, 0.074 mmol) were added successively. After being stirred at room temperature overnight, the mixture was cooled to 0 °C and acceptor **16** (100 mg, 0.069 mmol), NIS (26 mg, 0.11 mmol), TMSOTf (0.1 mL, 28 μL TMSOTf in 1.0 mL DCM) were added successively. The reaction mixture was allowed to warm to room temperature and stirred for 4 h. Then the reaction was quenched with Et_3N (1 mL) and filtered. The filtrate was concentrated under vacuum to give a residue, which was purified by flash column chromatography (PE/EA 4:1 to 2:1) to afford **19** (149.4 mg, 66% in one-pot) as a white foam. $[\alpha]_D^{20}$= -64.44 (*c* 0.27 $CHCl_3$). ^{1}H NMR (600 MHz, $CDCl_3$) δ 8.09 – 8.02 (m, 6H), 7.99 (d, *J* = 8.4 Hz, 1H), 7.91 (d, *J* = 8.0 Hz, 2H), 7.82 (d, *J* = 7.9 Hz, 2H), 7.77 (d, *J* = 8.9 Hz, 2H), 7.62 (d, *J* = 8.2 Hz, 2H), 7.57 – 7.51 (m, 4H), 7.51 – 7.45 (m, 2H), 7.43 – 7.33 (m, 22H), 7.32 – 7.22 (m, 28H), 7.22 – 7.17 (m, 20H), 7.17 – 7.07 (m, 10H), 7.00 – 6.92 (m, 5H), 6.92 – 6.89 (m, 1H), 6.87 (d, *J* = 7.3 Hz, 2H), 6.27 (t, *J* = 10.0 Hz, 1H), 5.71 – 5.67 (m, 1H), 5.65 (dd, *J* = 10.2, 3.2 Hz, 1H), 5.62 – 5.58 (m, 1H), 5.59 (d, *J* = 3.2 Hz, 1H), 5.56 (t, *J* = 2.5 Hz, 1H), 5.44 (s, 1H), 5.34 (s, 1H), 5.24 (s, 1H), 5.23 – 5.12 (m, 5H), 4.96 (d, *J* = 10.7 Hz, 1H), 4.94 – 4.86 (m, 3H), 4.83 (d, *J* = 10.8 Hz, 1H), 4.70 – 4.61 (m, 4H), 4.60 – 4.51 (m, 8H), 4.51 – 4.43 (m, 8H), 4.42 – 4.37 (m, 1H), 4.36 – 4.13 (m, 14H), 4.06 – 4.00 (m, 2H), 3.92 – 3.86 (m, 3H), 3.84 – 3.77 (m, 3H), 3.75 (t, *J* = 9.0 Hz, 1H), 3.73 – 3.65 (m, 5H), 3.61 – 3.53 (m, 4H), 3.52 – 3.48 (m, 2H), 3.45 – 3.38 (m, 2H), 3.33 (d, *J* = 11.1 Hz, 1H), 3.25 – 3.12 (m, 4H), 1.53 – 1.35 (m, 4H), 1.24 – 1.11 (m, 2H), 0.98 (s, 10H). ^{13}C NMR (151 MHz, $CDCl_3$) δ 165.8, 165.7, 165.6, 165.4, 165.3, 164.9, 156.8, 156.3, 138.7, 138.61, 138.55, 138.52, 138.46, 138.39, 138.37, 138.2, 138.0, 137.0, 135.8, 135.5, 133.3, 133.23, 133.15, 133.1, 133.05, 133.0, 132.7, 130.1, 130.0, 129.96, 129.9, 129.8, 129.74, 129.67, 129.6, 129.5, 129.4, 128.7, 128.64, 128.55, 128.53, 128.48, 128.46, 128.4,

128.34, 128.27, 128.11, 128.08, 128.0, 127.92, 127.87, 127.7, 127.64, 127.59, 127.55, 127.52, 127.46, 127.43, 127.37, 127.3, 127.2, 99.8, 99.52, 99.46, 99.4, 98.6, 79.4, 78.5, 78.4, 78.0, 77.9, 76.7, 75.7, 75.4, 75.31, 75.26, 75.0, 74.8, 74.5, 74.2, 74.1, 73.6, 73.5, 73.42, 73.37, 73.34, 73.30, 72.7, 72.62, 72.56, 72.53, 72.46, 72.4, 72.1, 71.9, 71.9, 71.6, 71.0, 70.6, 69.5, 69.2, 68.43, 68.38, 68.3, 67.6, 67.2, 65.6, 60.9, 53.6, 50.6, 50.3, 47.2, 46.2, 29.3, 28.0, 27.6, 26.6, 23.5, 19.2. MS (Maldi-TOF) m/z: $[M+Na]^+$ Calcd for $C_{198}H_{197}NO_{40}SiNa$ 3279.3073; Found 3279.3071.

Fully protected hexasaccharide 20

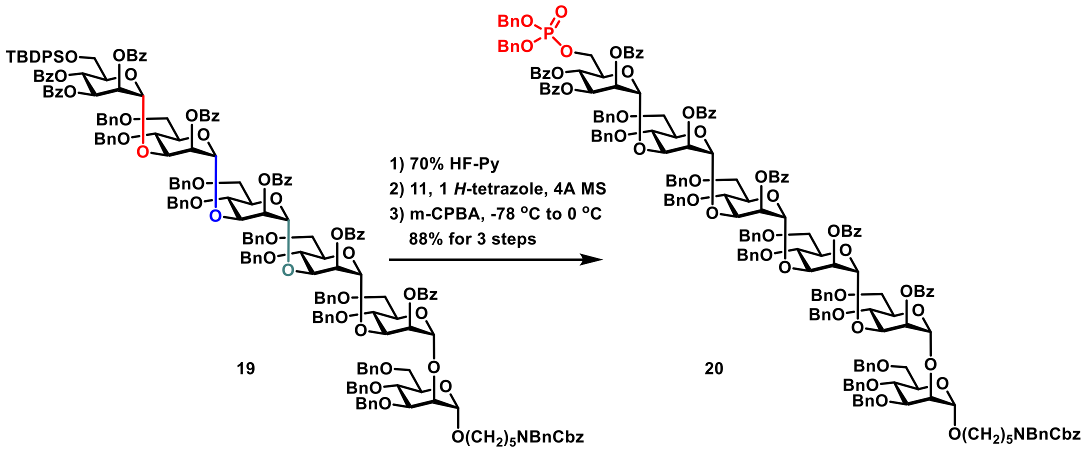

19

20

To a solution of **19** (515 mg, 0.16 mmol) in THF (2.1 mL) was added 70% HF·Py (67 μL, 0.47 mmol). The mixture was stirred at room temperature for 24 h and quenched with Et_3N (0.5 mL). Then, it was diluted with EtOAc (10.0 mL) and washed with saturated aqueous $NaHCO_3$ (10 mL × 3) and brine (30 mL) successively. The organic phase was dried with Na_2SO_4 and concentrated in vacuo, and the resulting residue was purified by silica gel column chromatography (PE/EA 4:1) to afford the intermediate as a white foam (436.6 mg, 91%). To a mixture of the above intermediate (335 mg, 0.11 mmol), 1*H*-tetrazole (55 mg, 0.78 mmol) and 4 Å MS in dry DCM (2.2 mL) was added dibenzyl *N*,*N*-diisopropylphosphoramidite (**11**, 200 μL, 0.55 mmol) dropwise over 5 minutes. After 16 h, the reaction mixture was cooled to −78 °C, and *m*-CPBA (77 mg of 85% *m*-CPBA, 0.44 mmol) was added as a solid. The reaction mixture was allowed to warm to 0 °C. After 4 h, saturated $Na_2S_2O_3$ was

added, and the organic layer was washed with saturated $NaHCO_3$, dried with Na_2SO_4, and concentrated under reduced pressure to give a residue, which was purified by flash column chromatography (PE/EA 2:1 to 1.5:1) to afford **20** (356 mg, 97% for 2 steps) as a white foam. $[\alpha]_D^{20}$= -64.44 (*c* 0.27 $CHCl_3$). ^1H NMR (400 MHz, $CDCl_3$) δ 8.11 – 7.87 (m, 12H), 7.76 (d, *J* = 7.2 Hz, 2H), 7.72 (d, *J* = 7.7 Hz, 2H), 7.58 (t, *J* = 7.4 Hz, 1H), 7.51 – 7.39 (m, 7H), 7.39 – 7.30 (m, 24H), 7.30 – 7.11 (m, 50H), 7.11 – 7.04 (m, 10H), 7.03 – 6.95 (m, 2H), 6.03 (t, *J* = 9.9 Hz, 1H), 5.65 (dd, *J* = 9.9, 3.1 Hz, 1H), 5.63 – 5.52 (m, 5H), 5.33 (s, 1H), 5.24 – 5.00 (m, 12H), 4.99 – 4.76 (m, 10H), 4.66 – 4.37 (m, 17H), 4.36 – 4.18 (m, 8H), 4.18 – 4.08 (m, 6H), 4.05 – 4.00 (m, 1H), 4.00 – 3.94 (m, 1H), 3.92 – 3.83 (m, 3H), 3.83 – 3.76 (m, 4H), 3.75 – 3.63 (m, 5H), 3.60 – 3.45 (m, 5H), 3.35 (t, *J* = 9.1 Hz, 2H), 3.25 – 3.10 (m, 3H), 1.55 – 1.33 (m, 4H), 1.22 – 1.10 (m, 2H). ^{13}C NMR (101 MHz, $CDCl_3$) δ 168.1, 165.8, 165.69, 165.66, 165.6, 165.5, 165.1, 164.8, 141.0, 138.60, 138.55, 138.52, 138.47, 138.42, 138.36, 138.3, 138.2, 138.0, 136.0, 135.9, 135.8, 134.5, 133.4, 133.2, 133.1, 131.8, 130.2, 130.04, 129.95, 129.9, 129.8, 129.82, 129.80, 129.73, 129.69, 129.67, 129.6, 129.33, 129.31, 128.69, 128.66, 128.62, 128.58, 128.54, 128.50, 128.48, 128.45, 128.44, 128.42, 128.38, 128.36, 128.3, 128.2, 128.1, 128.03, 127.95, 127.93, 127.90, 127.8, 127.69, 127.66, 127.62, 127.57, 127.54, 127.52, 127.48, 127.46, 127.42, 127.35, 127.1, 99.7, 99.5, 99.4, 98.6, 79.4, 78.6, 78.2, 78.1, 77.9, 77.4, 76.7, 75.7, 75.4, 75.3, 75.2, 75.0, 74.9, 74.4, 74.22, 74.15, 73.44, 73.38, 73.35, 73.3, 72.6, 72.53, 72.49, 72.4, 72.1, 72.0, 71.9, 70.3, 70.0, 69.9, 69.5, 69.4, 69.3, 69.2, 68.3, 67.6, 67.3, 65.5, 65.4, 65.0, 50.3, 29.3, 28.0, 27.6, 23.5. MS (Maldi-TOF) m/z: $[M+Na]^+$ Calcd for $C_{196}H_{192}NO_{43}PNa$ 3301.2498; Found 3301.2499.

Hexasaccharide 4

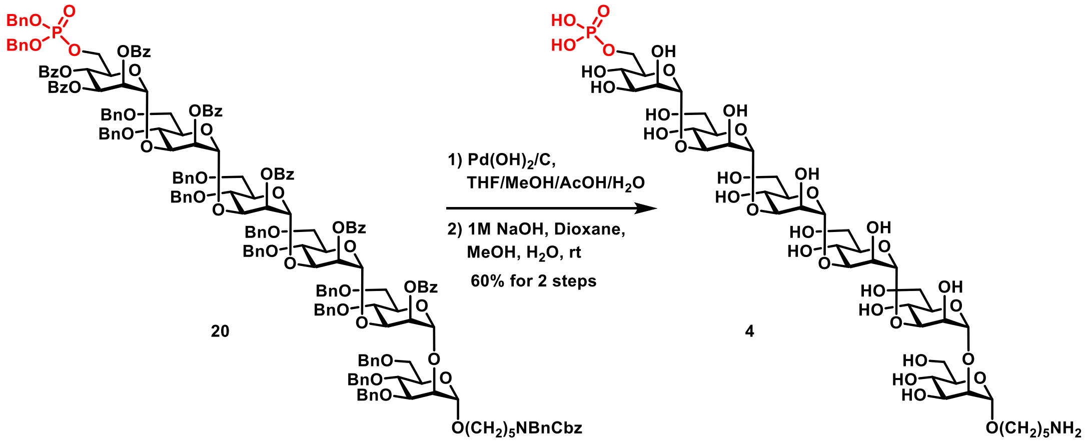

A solution of **20** (100 mg, 0.031 mmol) and $Pd(OH)_2/C$ (321 mg, 20%) in THF/MeOH/H_2O/HOAc (1.5 mL/2.0 mL/0.5 mL/0.2 mL) was stirred under an atmosphere of H_2 at room temperature for 2 days. The reaction mixture was filtered and concentrated in vacuo to afford the intermediate. To a solution of the above intermediate in dioxane/MeOH (1.4 mL/0.5 mL) was added 1 M NaOH (2.0 mL). The reaction mixture was stirred for 2 d at room temperature. Then, the reaction mixture was neutralized to pH 7 by acetic acid, filtered and concentrated. The residue was purified by column chromatography on Sephadex TM LH-20 (MeOH-H_2O, 1:1) to afford deprotection product **4** (21 mg, 60% for 2 steps). $[\alpha]_D^{25}$= +116.87 (*c* 0.30 H_2O). ^{1}H NMR (400 MHz, D_2O) δ 5.15 – 5.12 (m, 3H), 5.11 (s, 1H), 5.09 (s, 1H), 5.02 (s, 1H), 4.38 (t, *J* = 2.6 Hz, 1H), 4.26 – 4.21 (m, 3H), 4.17 (dd, *J* = 10.0, 6.5 Hz, 1H), 4.09 (dd, *J* = 3.6, 1.7 Hz, 1H), 4.05 – 4.00 (m, 2H), 3.99 – 3.95 (m, 3H), 3.95 – 3.87 (m, 8H), 3.87 – 3.83 (m, 2H), 3.83 – 3.78 (m, 5H), 3.78 – 3.72 (m, 6H), 3.71 – 3.69 (m, 1H), 3.67 – 3.60 (m, 2H), 3.58 – 3.52 (m, 1H), 3.10 – 2.98 (m, 2H), 1.79 – 1.60 (m, 4H), 1.52 – 1.41 (m, 2H). ^{13}C NMR (151 MHz, D_2O) δ 102.8, 102.5, 102.2, 102.1, 102.0, 98.0, 79.3, 78.7, 78.3, 78.0, 77.9, 73.7, 73.4, 73.4, 72.8, 72.6, 72.5, 70.2, 70.0, 69.7, 69.6, 69.5, 69.3, 67.45, 67.40, 66.9, 66.7, 66.2, 66.1, 65.8, 65.8, 63.9, 61.1, 61.0, 60.8, 47.4, 47.3, 39.3, 28.3, 27.9, 26.5, 25.3, 22.5, 22.4, 22.3. MS (ESI$^-$) calcd for $C_{41}H_{74}NO_{34}P$ [M-H]$^-$, 1154.3757, found 1154.3751.

7. Confirmation of the structures of PI-88 glycan motifs 1–4

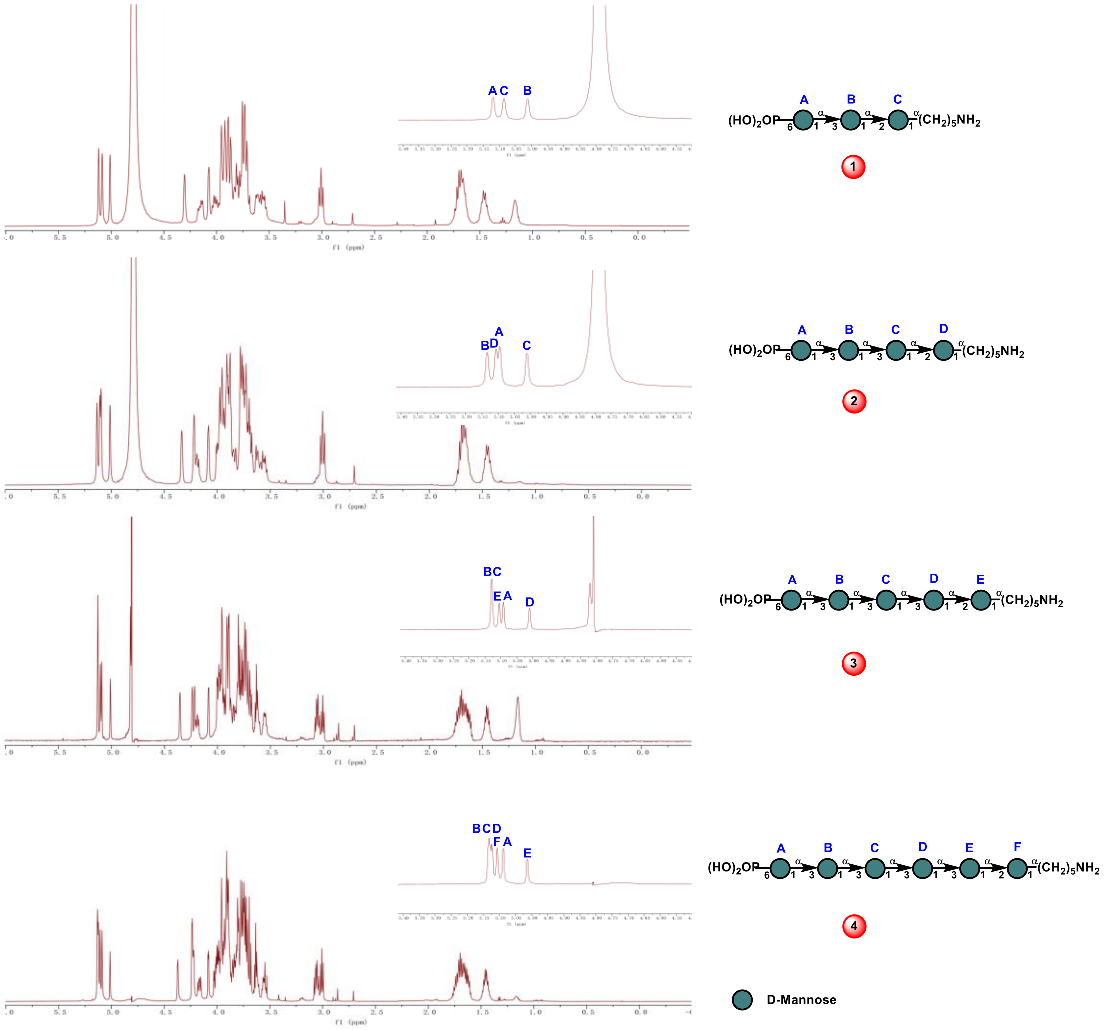

Figure 1. The evidence of structures of PI-88 glycans motifs **1–4**.

8. References

[1] Patil P S, Cheng T J R, Zulueta M M L, et al. Total synthesis of tetraacylated phosphatidylinositol hexamannoside and evaluation of its immunomodulatory activity. *Nat Commun* **2015**, *6*, 7239.

[2] Seeberger P H, Martin C E, Broecker F, et al. Oligosaccharides and oligosaccharide-protein conjugates derived from clostridium difficile polysaccharide ps-ii, methods of synthesis and uses thereof, in particular as a vaccine, WO2012119769.

[3] Jayaprakash K N, Lu J, Fraser-Reid B. Synthesis of a Lipomannan Component of the Cell-Wall Complex of Mycobacterium tuberculosis Is Based on Paulsen's Concept of Donor/Acceptor “Match”. *Angew. Chem. Int. Ed.* **2005**, *44*, 5894.

9. Spectroscopic data

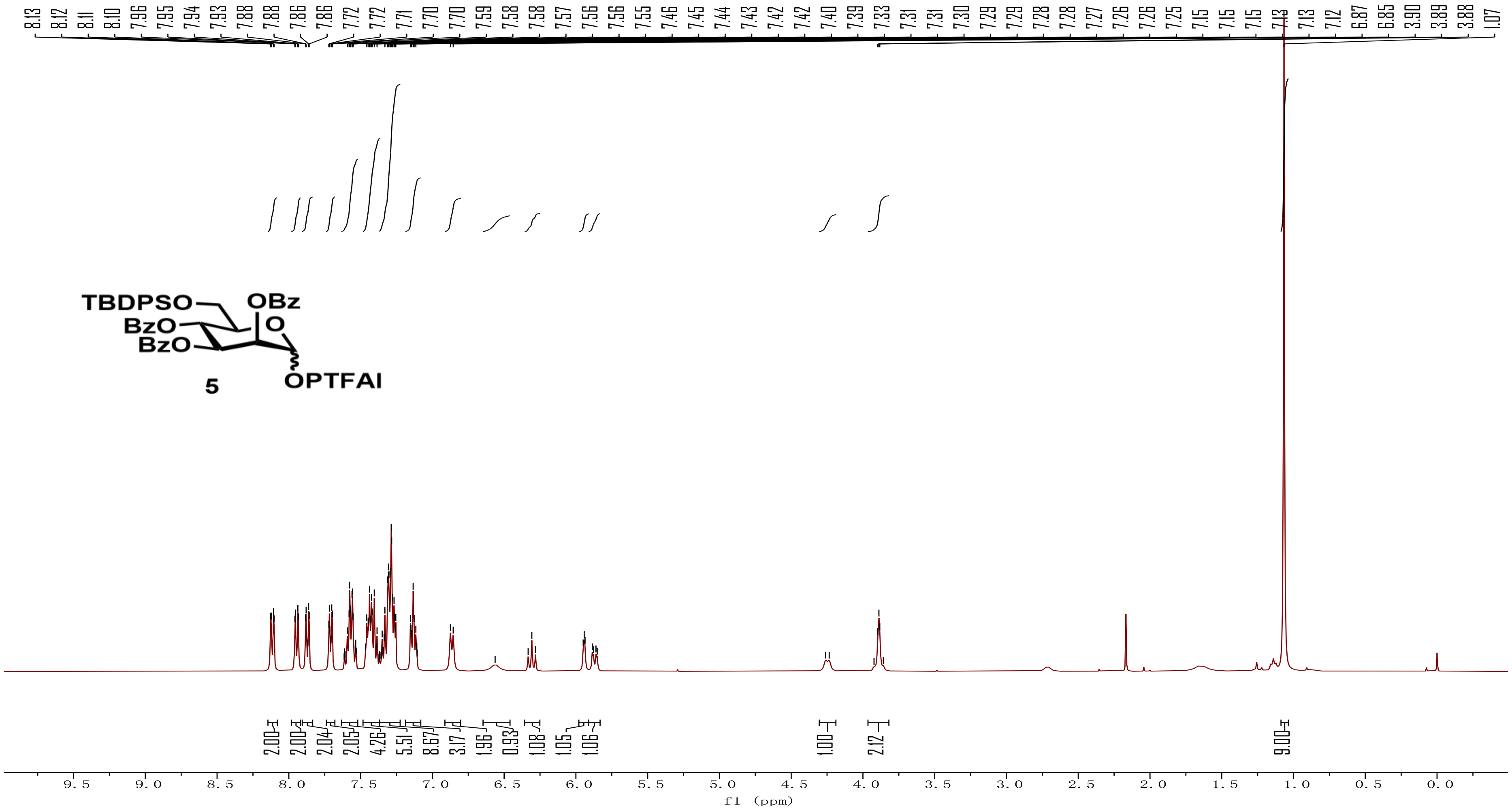

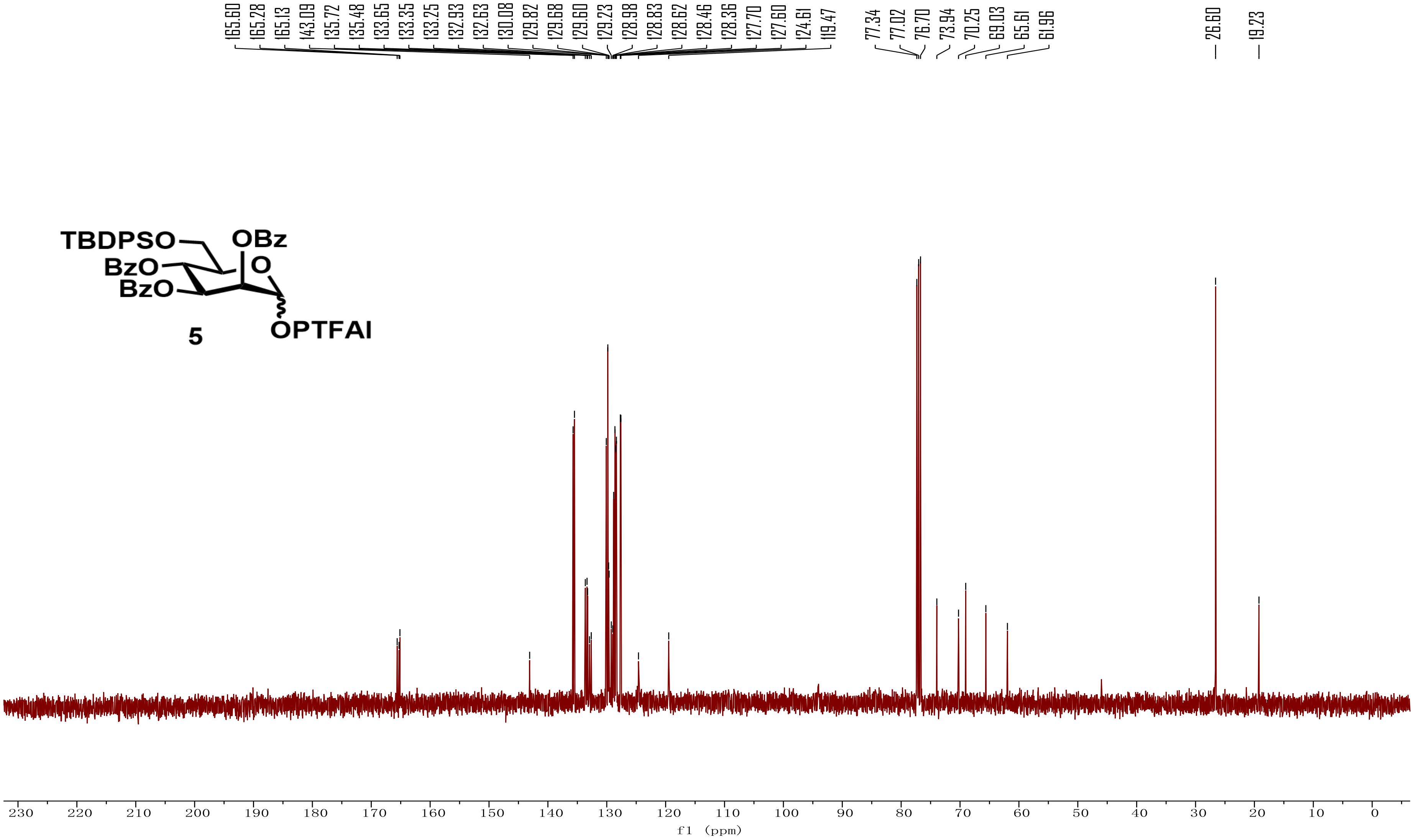
165.60
165.28
165.13
143.09
135.72
135.48
133.65
133.35
133.25
132.93
132.63
130.08
129.82
129.68
129.60
129.23
128.98
128.83
128.62
128.46
128.36
127.70
127.60
124.61
119.47
77.34
77.02
76.70
73.94
70.25
69.03
65.61
61.96
26.60
19.23
TBDPSO
OBz
BzO
BzO
O
OPTFAI
5
230
220
210
200
190
180
170
160
150
140
130
120
110
100
90
80
70
60
50
40
30
20
10
0
f1 (ppm)

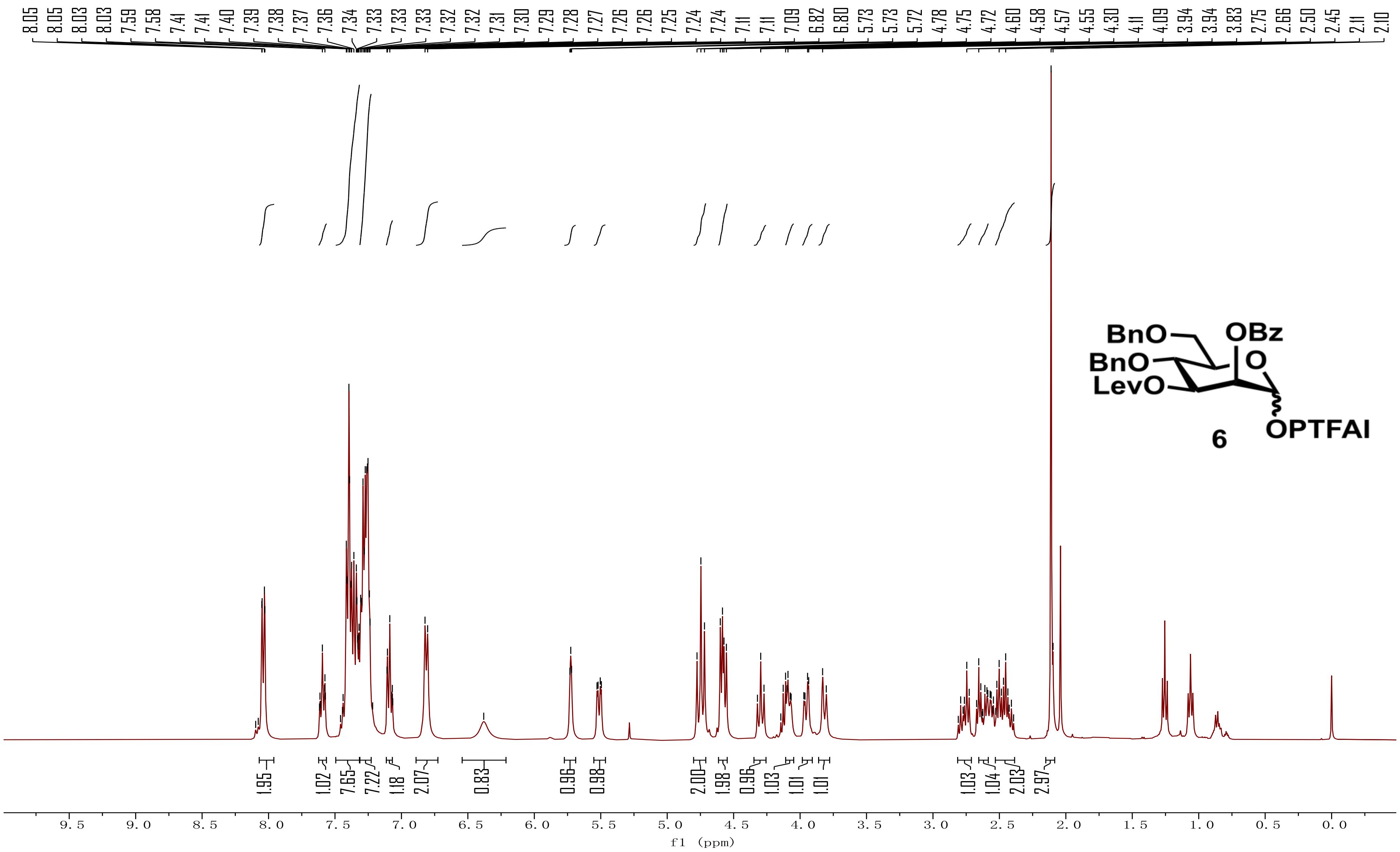
BnO
OBz
BnO
O
LevO
OPTFAI
6
f1 (ppm)

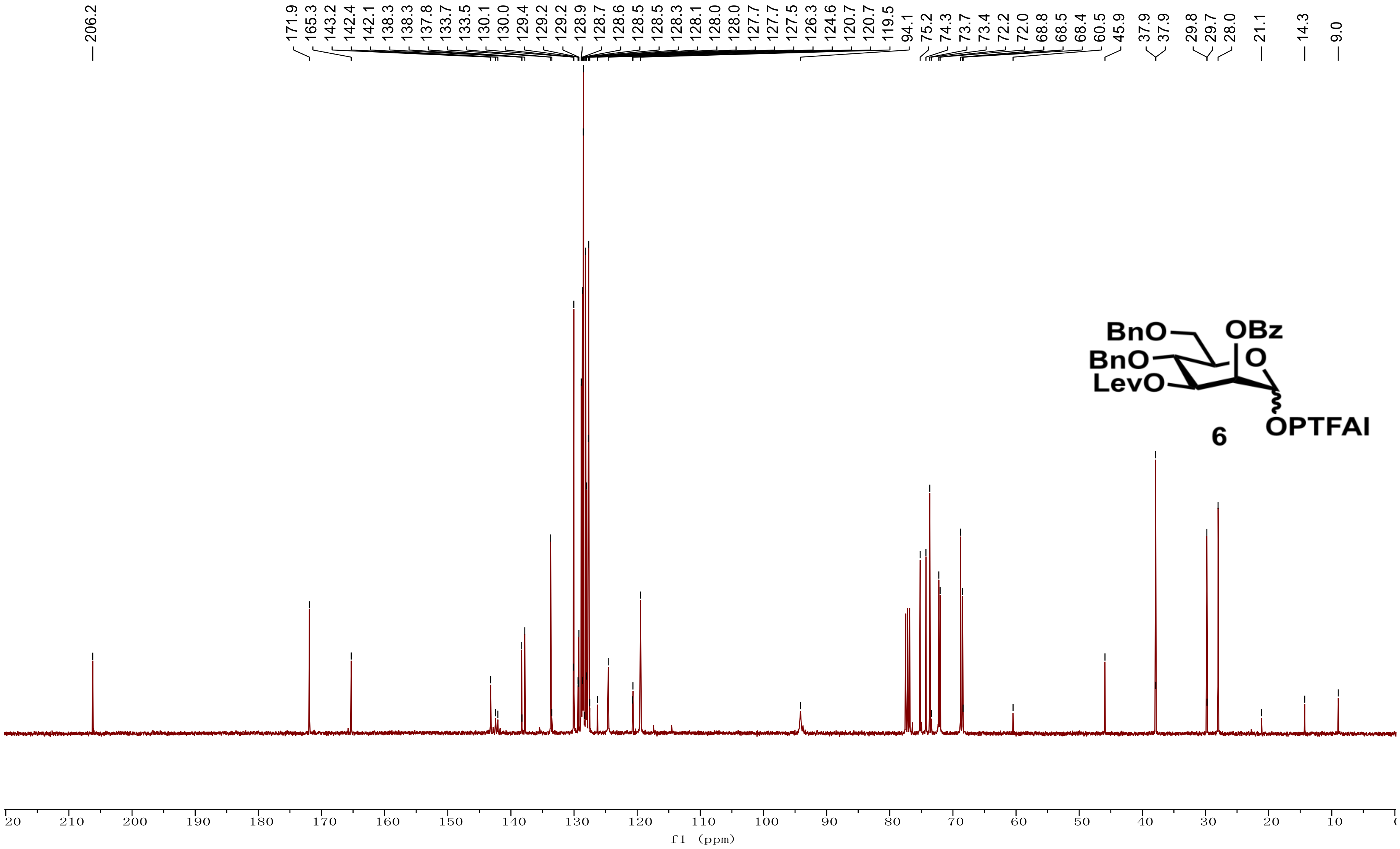
206.2
171.9
165.3
143.2
142.4
142.1
138.3
138.3
137.8
133.7
133.5
130.1
130.0
129.4
129.2
129.2
128.9
128.7
128.6
128.5
128.5
128.3
128.1
128.0
128.0
127.7
127.7
127.5
126.3
124.6
120.7
120.7
119.5
94.1
75.2
74.3
73.7
73.4
72.2
72.0
68.8
68.5
68.4
60.5
45.9
37.9
37.9
29.8
29.7
28.0
21.1
14.3
9.0
BnO
OBz
BnO
O
LevO
OPTFAI
6
f1 (ppm)

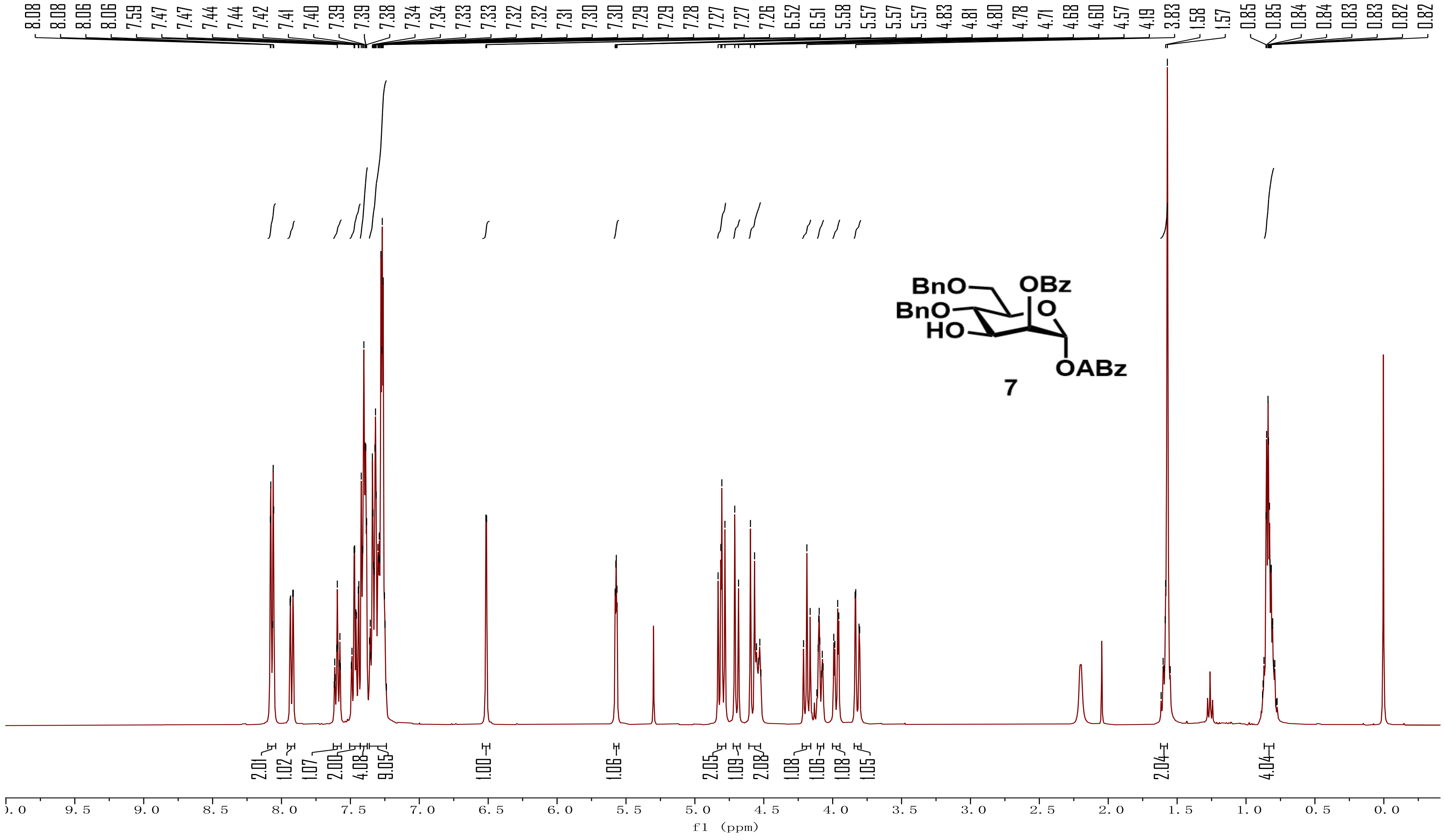

8.08 8.08 8.06 8.06 7.59 7.47 7.47 7.44 7.44 7.42 7.41 7.40 7.39 7.39 7.38 7.34 7.34 7.33 7.33 7.32 7.32 7.31 7.30 7.30 7.29 7.29 7.28 7.27 7.27 7.26 6.52 6.51 5.58 5.57 5.57 5.57 4.83 4.81 4.80 4.78 4.71 4.68 4.60 4.57 4.19 3.83 1.58 1.57 0.85 0.85 0.84 0.84 0.83 0.83 0.82 0.82
BnO
BnO
HO
OBz
O
OABz
7
2.01 1.02 1.07 2.00 4.08 9.05 1.00 1.06 2.05 1.09 2.08 1.08 1.06 1.08 1.05 2.04 4.04
f1 (ppm)

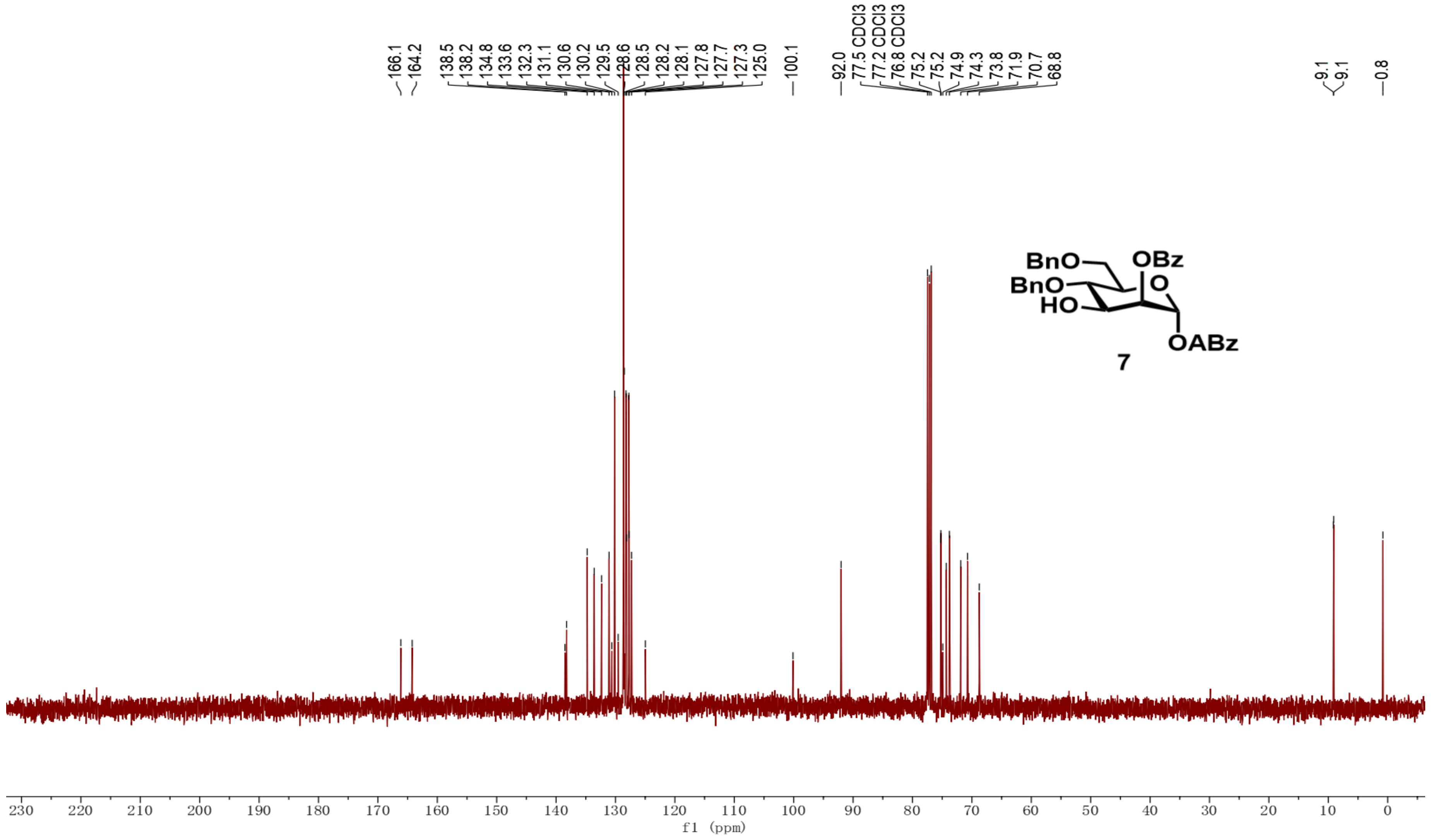
166.1
164.2
138.5
138.2
134.8
133.6
132.3
131.1
130.6
130.2
129.5
128.6
128.5
128.2
128.1
127.8
127.7
127.3
125.0
100.1
92.0
77.5 CDCl3
77.2 CDCl3
76.8 CDCl3
75.2
75.2
74.9
74.3
73.8
71.9
70.7
68.8
9.1
9.1
0.8
BnO
BnO
HO
OBz
O
OABz
7
230 220 210 200 190 180 170 160 150 140 130 120 110 100 90 80 70 60 50 40 30 20 10 0
f1 (ppm)

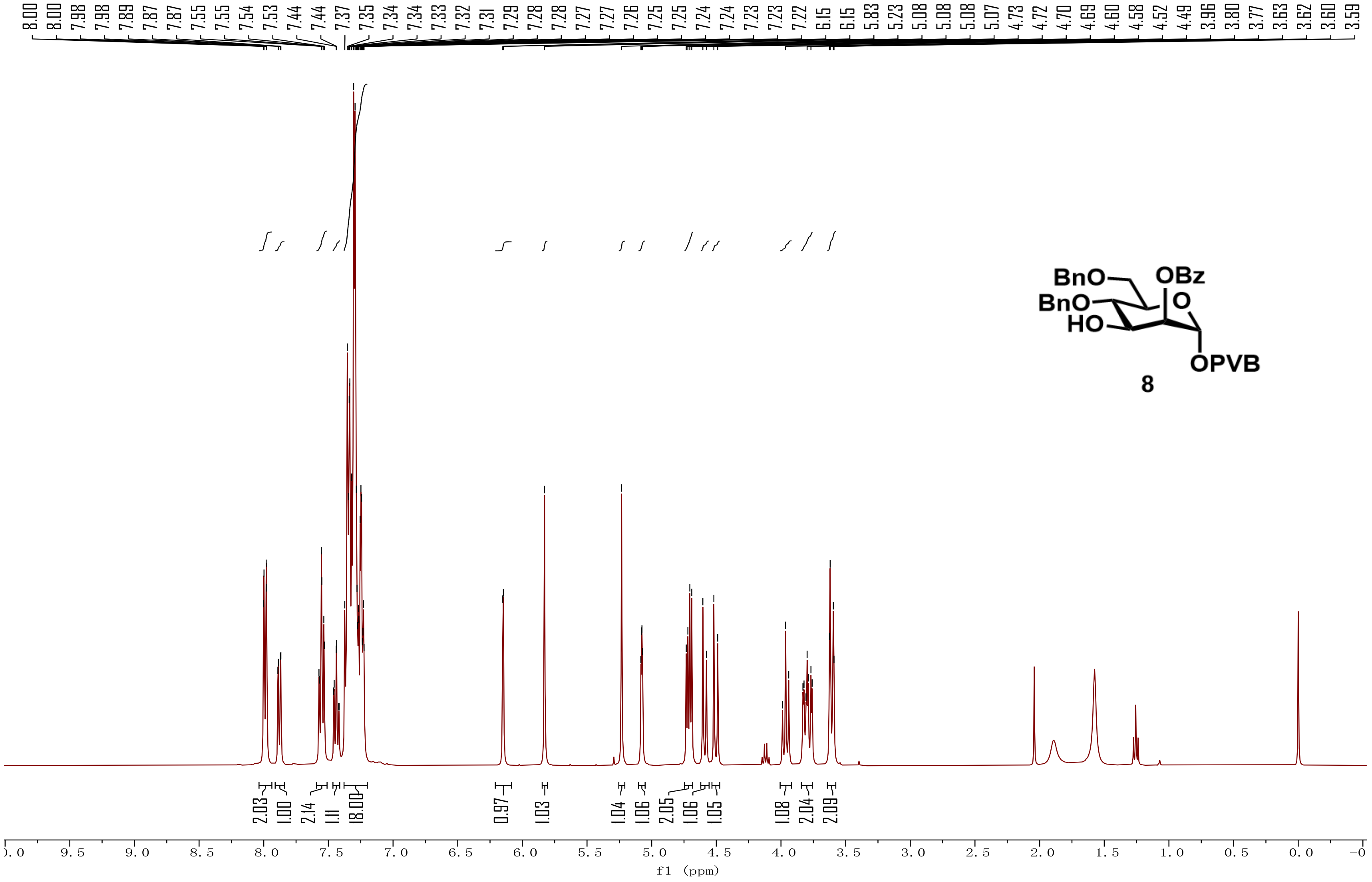
BnO
BnO
HO
OBz
O
OPVB
8
f1 (ppm)

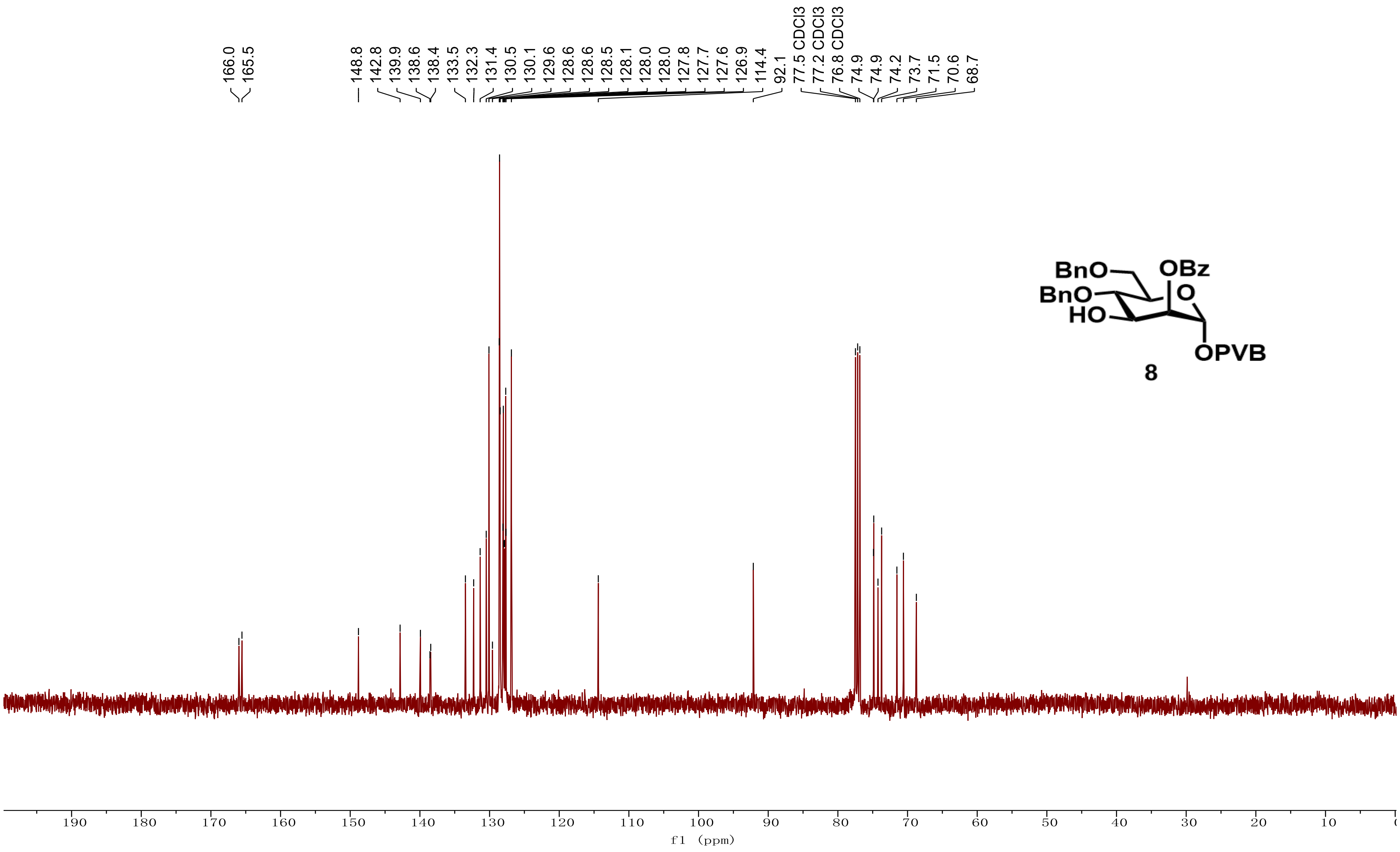
166.0
165.5
148.8
142.8
139.9
138.6
138.4
133.5
132.3
131.4
130.5
130.1
129.6
128.6
128.6
128.5
128.1
128.0
128.0
127.8
127.7
127.6
126.9
114.4
92.1
77.5 CDCl3
77.2 CDCl3
76.8 CDCl3
74.9
74.9
74.2
73.7
71.5
70.6
68.7
190 180 170 160 150 140 130 120 110 100 90 80 70 60 50 40 30 20 10
f1 (ppm)
BnO
BnO
HO
OBz
O
OPVB

8

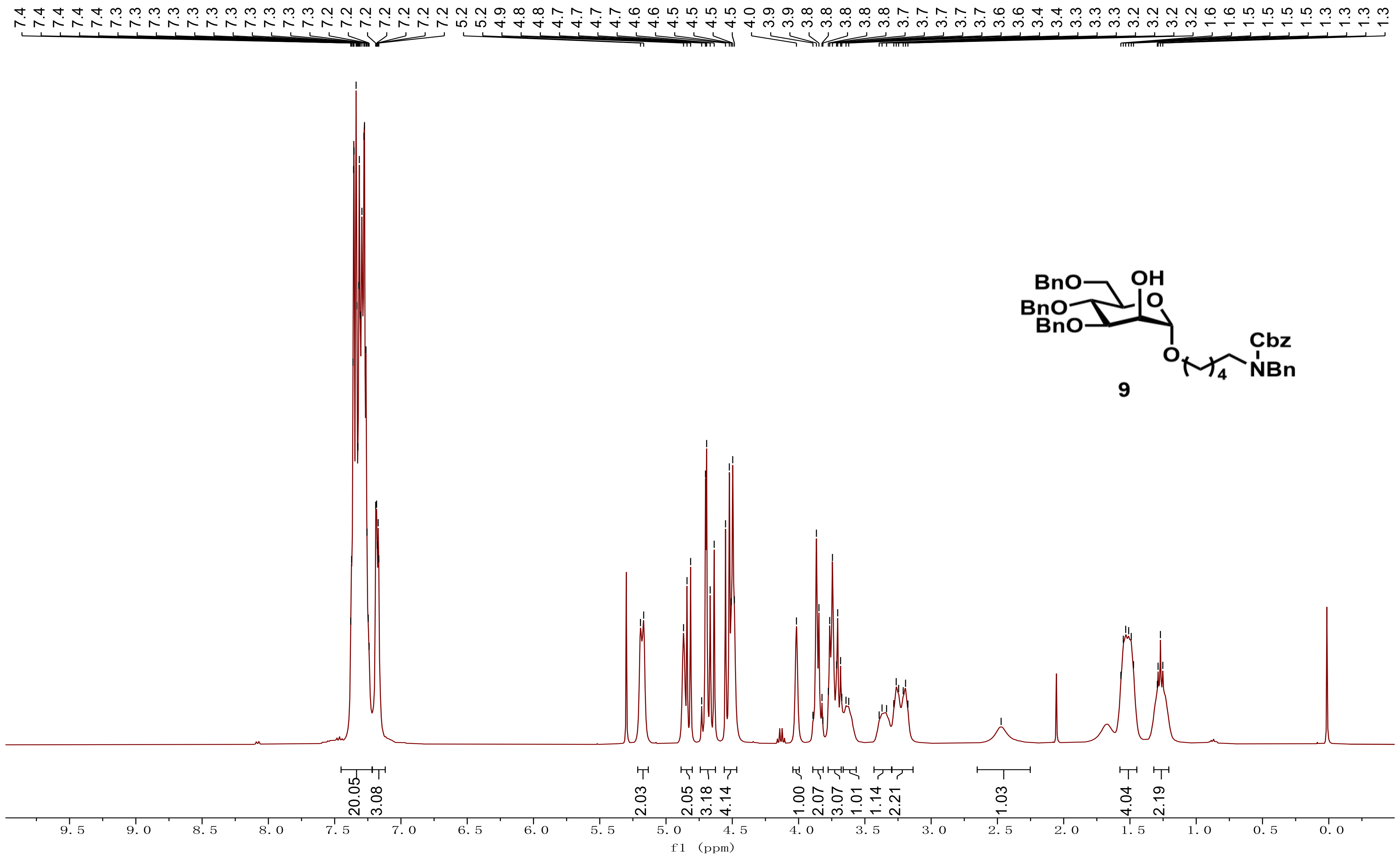

BnO
BnO
BnO
OH
O
O
Cbz
NBn
4
9
20.05
3.08
2.03
2.05
3.18
4.14
1.00
2.07
3.07
1.01
1.14
2.21
1.03
4.04
2.19
9.5 9.0 8.5 8.0 7.5 7.0 6.5 6.0 5.5 5.0 4.5 4.0 3.5 3.0 2.5 2.0 1.5 1.0 0.5 0.0
f1 (ppm)

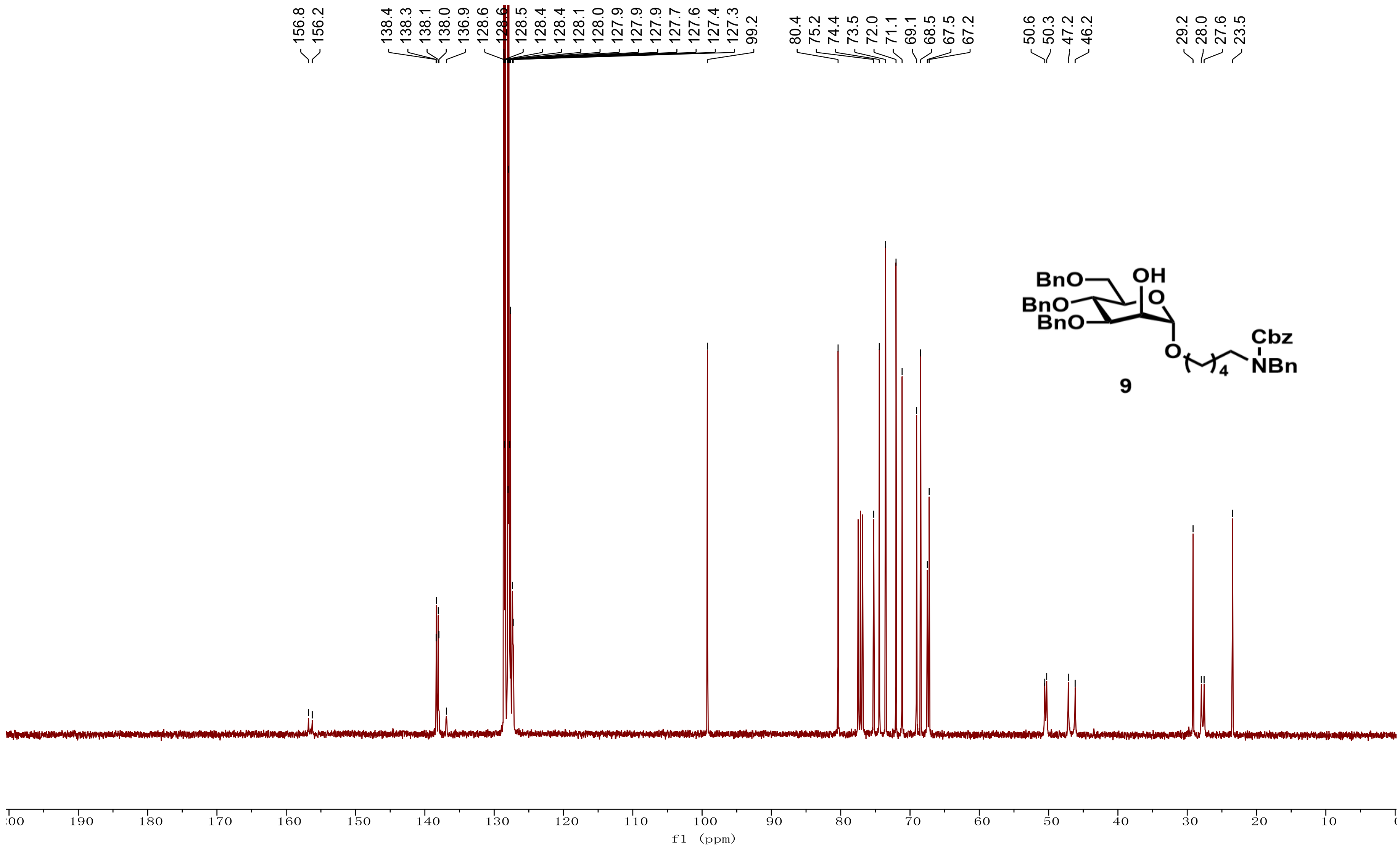
156.8
156.2
138.4
138.3
138.1
138.0
136.9
128.6
128.6
128.5
128.4
128.4
128.1
128.0
127.9
127.9
127.9
127.7
127.6
127.4
127.3
99.2
80.4
75.2
74.4
73.5
72.0
71.1
69.1
68.5
67.5
67.2
50.6
50.3
47.2
46.2
29.2
28.0
27.6
23.5
BnO
BnO
BnO
OH
O
O
4
Cbz
NBn
9
200
190
180
170
160
150
140
130
120
110
100
90
80
70
60
50
40
30
20
10
0
f1 (ppm)

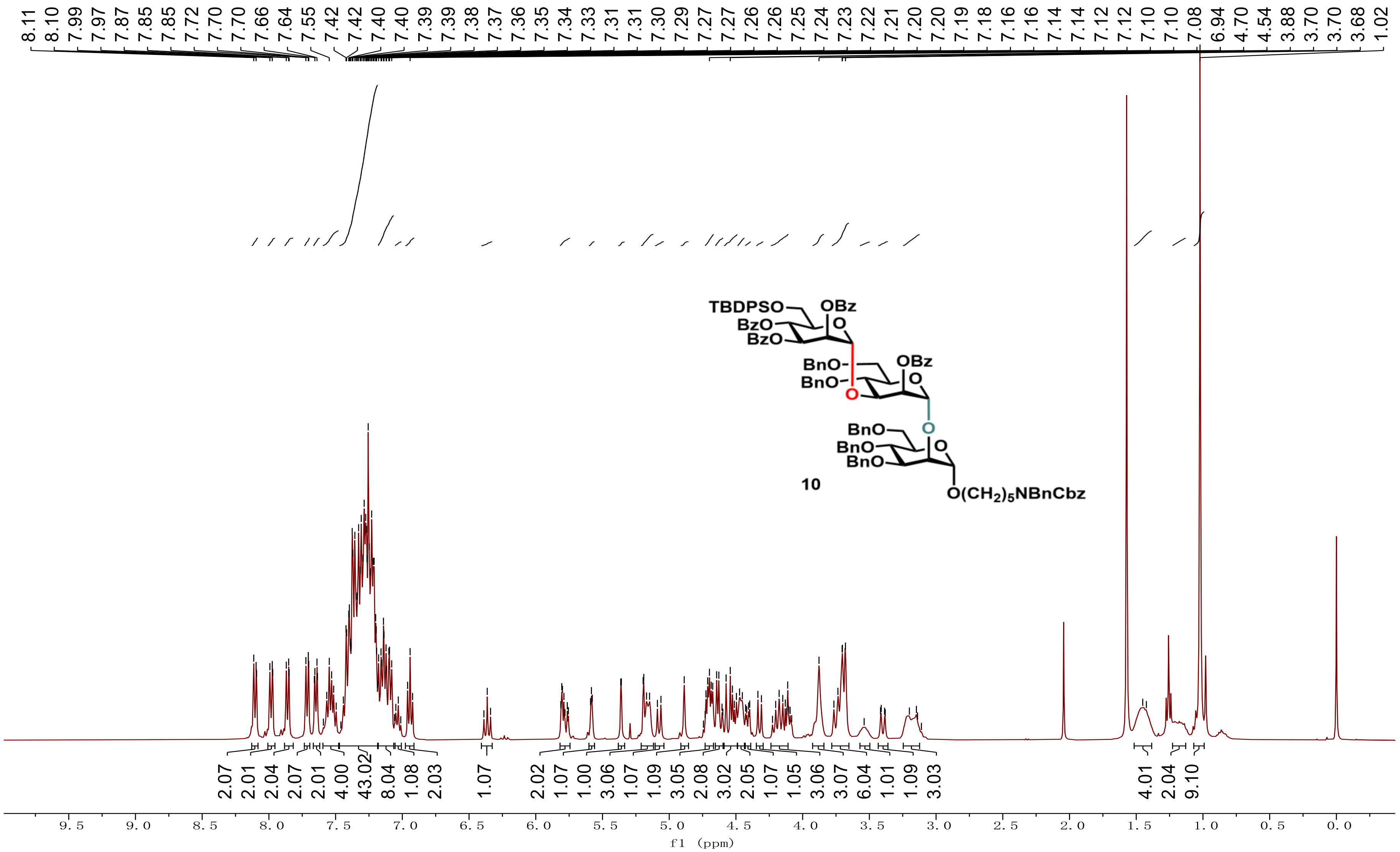
8.11 8.10 7.99 7.97 7.87 7.85 7.85 7.72 7.70 7.70 7.66 7.64 7.55 7.42 7.42 7.40 7.40 7.39 7.39 7.38 7.37 7.36 7.35 7.34 7.33 7.31 7.31 7.30 7.29 7.27 7.27 7.26 7.26 7.25 7.24 7.23 7.22 7.21 7.20 7.20 7.19 7.18 7.16 7.16 7.14 7.14 7.12 7.12 7.10 7.10 7.08 6.94 4.70 4.54 3.88 3.70 3.70 3.68 1.02
TBDPSO
OBz
BzO
BzO
BnO
BnO
OBz
BnO
BnO
BnO
10
O(CH2)5NBnCbz
2.07 2.01 2.04 2.07 2.01 4.00 43.02 8.04 1.08 2.03 1.07 2.02 1.07 1.00 3.06 1.07 1.09 3.05 2.08 3.02 2.05 1.07 1.05 3.06 3.07 6.04 1.01 1.09 3.03 4.01 2.04 9.10
9.5 9.0 8.5 8.0 7.5 7.0 6.5 6.0 5.5 5.0 4.5 4.0 3.5 3.0 2.5 2.0 1.5 1.0 0.5 0.0
f1 (ppm)

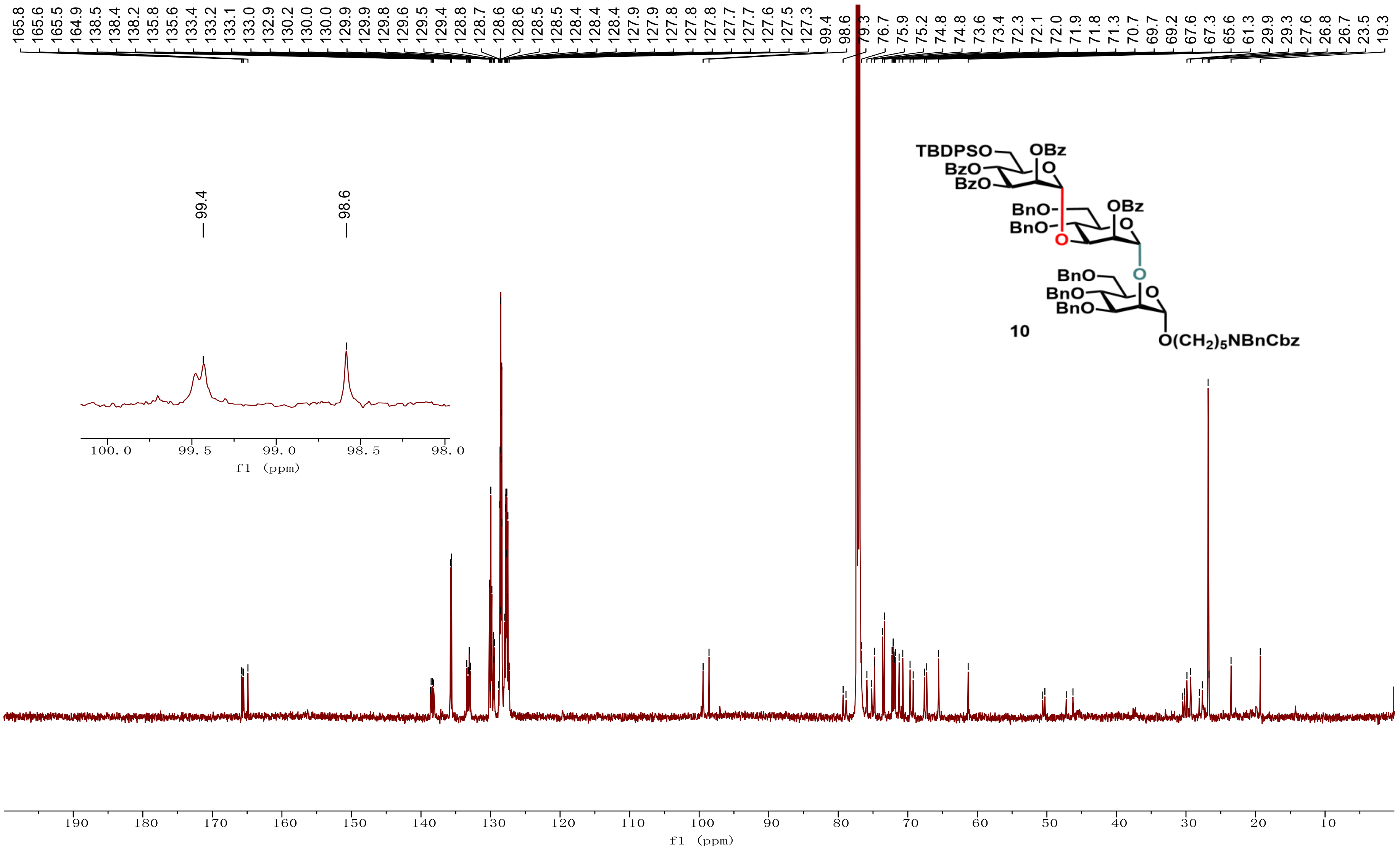

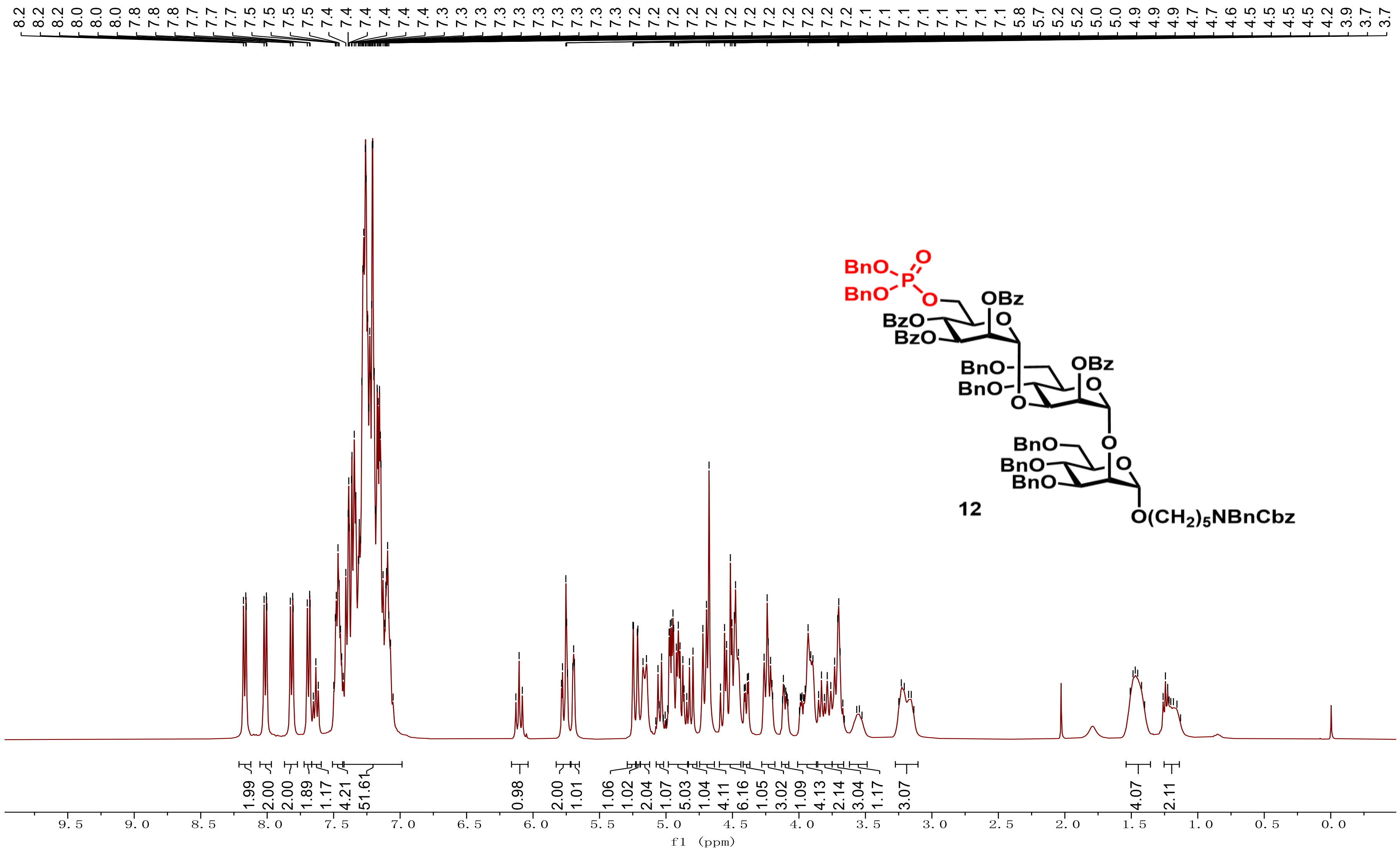

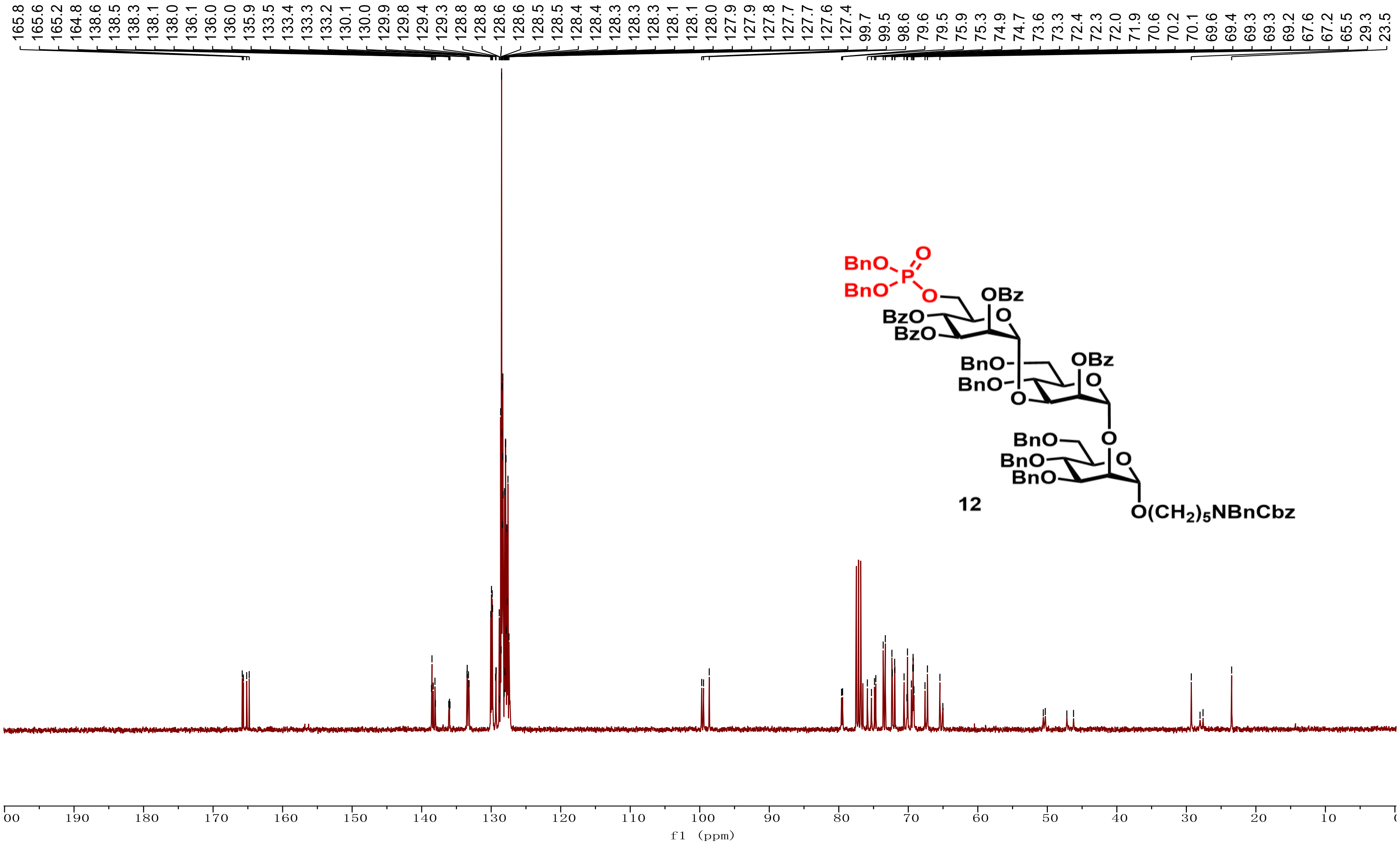
165.8
165.6
165.2
164.8
138.6
138.5
138.3
138.1
138.0
136.1
136.0
136.0
135.9
133.5
133.4
133.3
133.2
130.1
130.0
129.9
129.8
129.4
129.3
128.8
128.8
128.6
128.6
128.5
128.5
128.4
128.4
128.3
128.3
128.3
128.1
128.1
128.0
127.9
127.9
127.8
127.7
127.7
127.6
127.4
99.7
99.5
98.6
79.6
79.5
75.9
75.3
74.9
74.7
73.6
73.3
72.4
72.3
72.0
71.9
70.6
70.2
70.1
69.6
69.4
69.3
69.3
69.2
67.6
67.2
65.5
29.3
23.5
BnO
BnO
P
O
O
OBz
O
BzO
BzO
BnO
BnO
OBz
O
O
O
BnO
BnO
BnO
O
12
O(CH2)5NBnCbz
00
190
180
170
160
150
140
130
120
110
100
90
80
70
60
50
40
30
20
10
f1 (ppm)

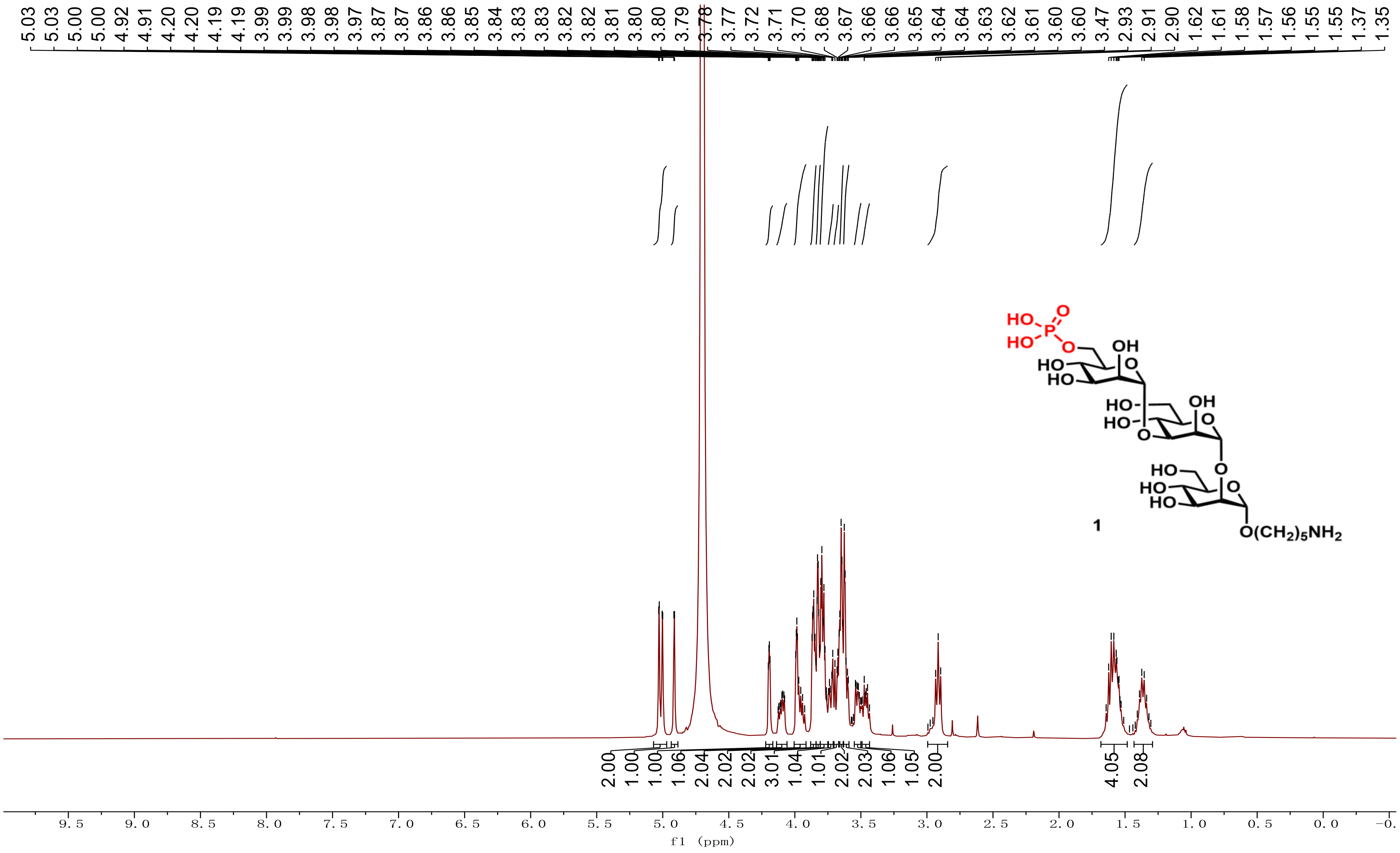
5.03 5.03 5.00 5.00 4.92 4.91 4.20 4.20 4.19 4.19 3.99 3.99 3.98 3.98 3.97 3.87 3.87 3.86 3.86 3.85 3.84 3.83 3.83 3.82 3.82 3.81 3.80 3.80 3.79 3.78 3.77 3.72 3.71 3.70 3.68 3.67 3.66 3.66 3.65 3.64 3.64 3.63 3.62 3.61 3.60 3.60 3.47 2.93 2.91 2.90 1.62 1.61 1.58 1.57 1.56 1.55 1.55 1.37 1.35
2.00 1.00 1.00 1.06 2.04 2.02 2.02 3.01 1.04 1.01 2.02 2.03 1.06 1.05 2.00 4.05 2.08
HO
HO
P
O
O
OH
HO
HO
O
HO
HO
OH
O
O
O
HO
HO
HO
O
O(CH2)5NH2
1
f1 (ppm)

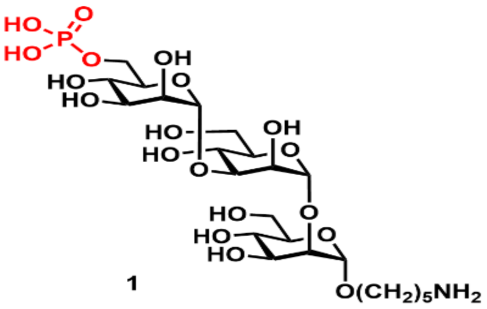
HO
HO
P
O
O
OH
HO
HO
O
HO
HO
O
OH
O
O
HO
HO
HO
O
O(CH2)5NH2

1

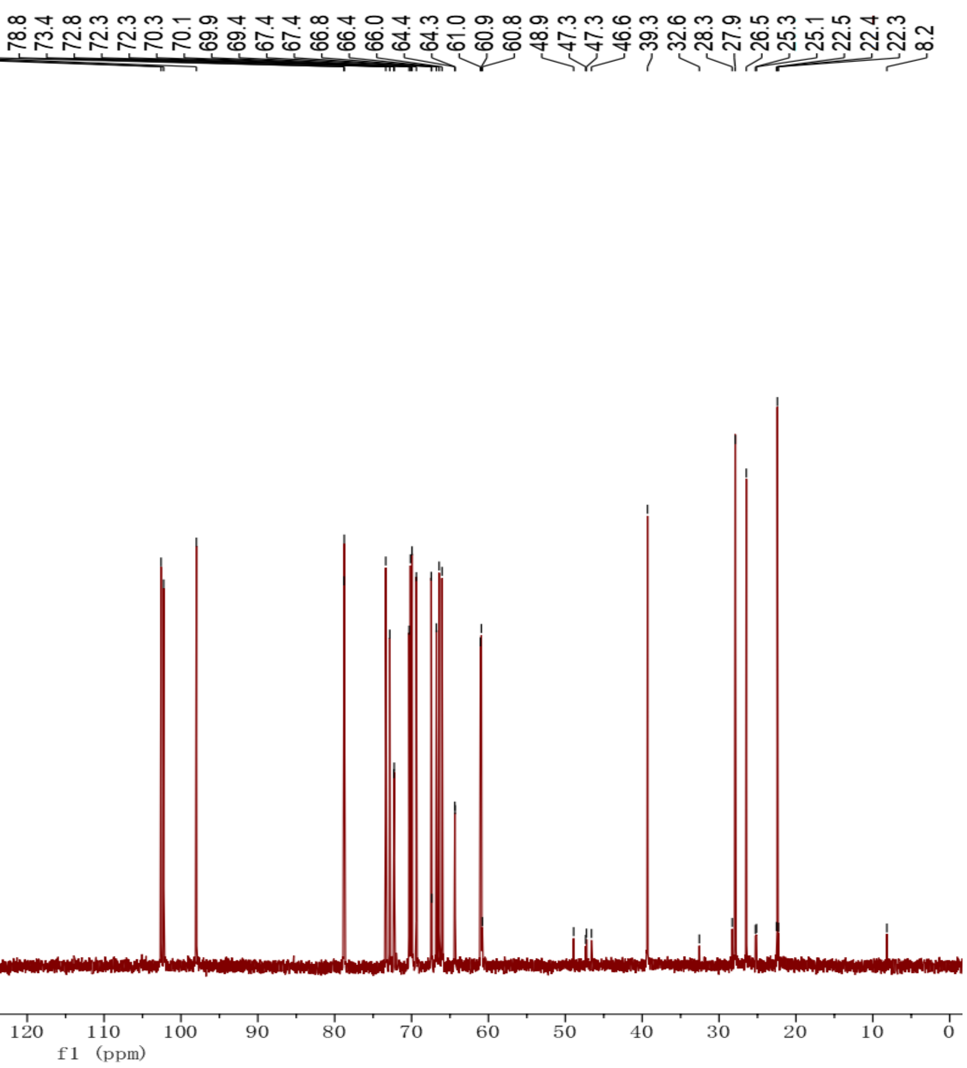
102.6
102.2
98.0
78.8
78.8
73.4
72.8
72.3
72.3
70.3
70.1
69.9
69.4
67.4
67.4
66.8
66.4
66.0
64.4
64.3
61.0
60.9
60.8
48.9
47.3
47.3
46.6
39.3
32.6
28.3
27.9
26.5
25.3
25.1
22.5
22.4
22.3
8.2
230 220 210 200 190 180 170 160 150 140 130 120 110 100 90 80 70 60 50 40 30 20 10 0
f1 (ppm)

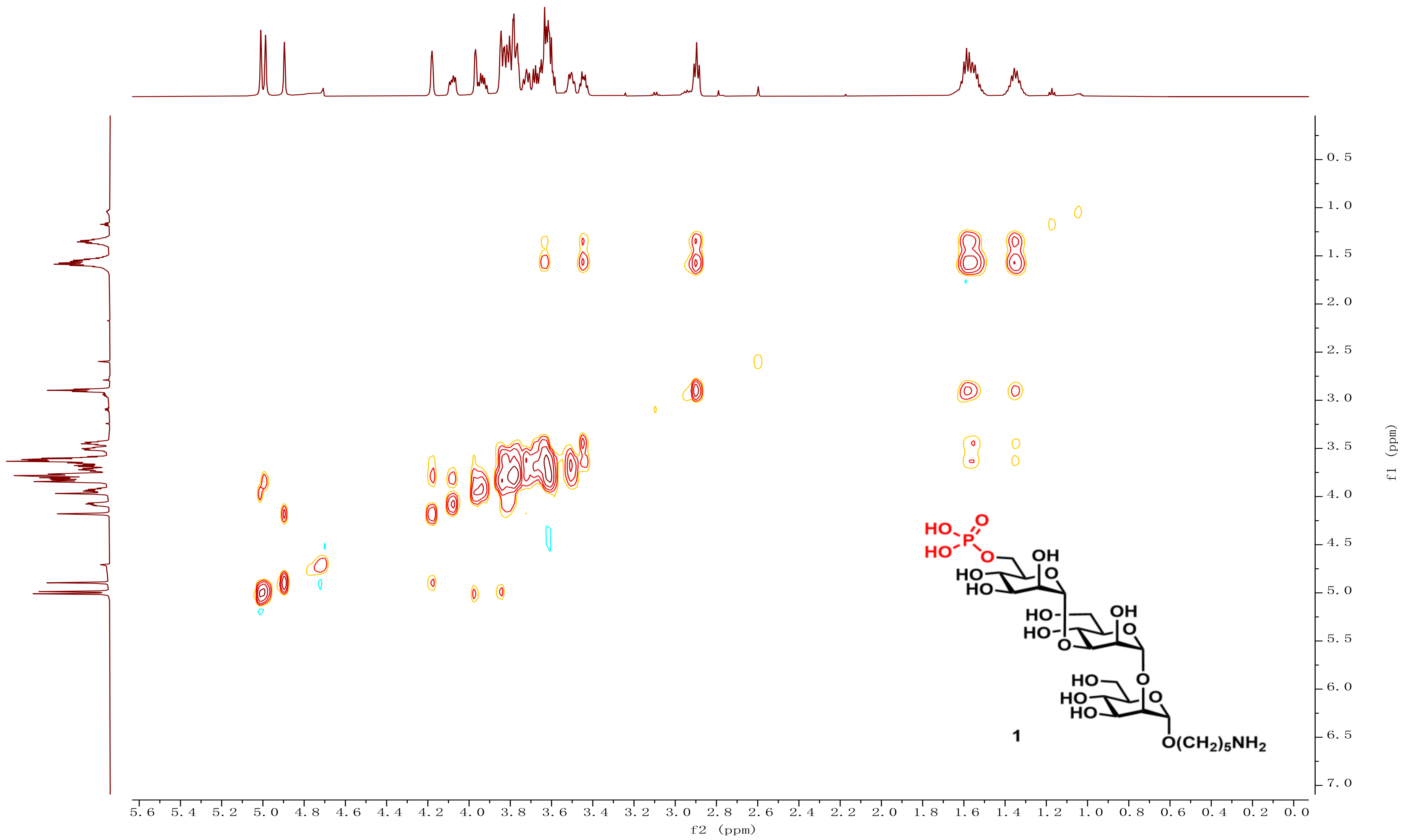
f2 (ppm)
f1 (ppm)
HO
HO
P
O
O
OH
HO
HO
O
HO
HO
OH
O
O
O
HO
HO
HO
O
O(CH2)5NH2
1

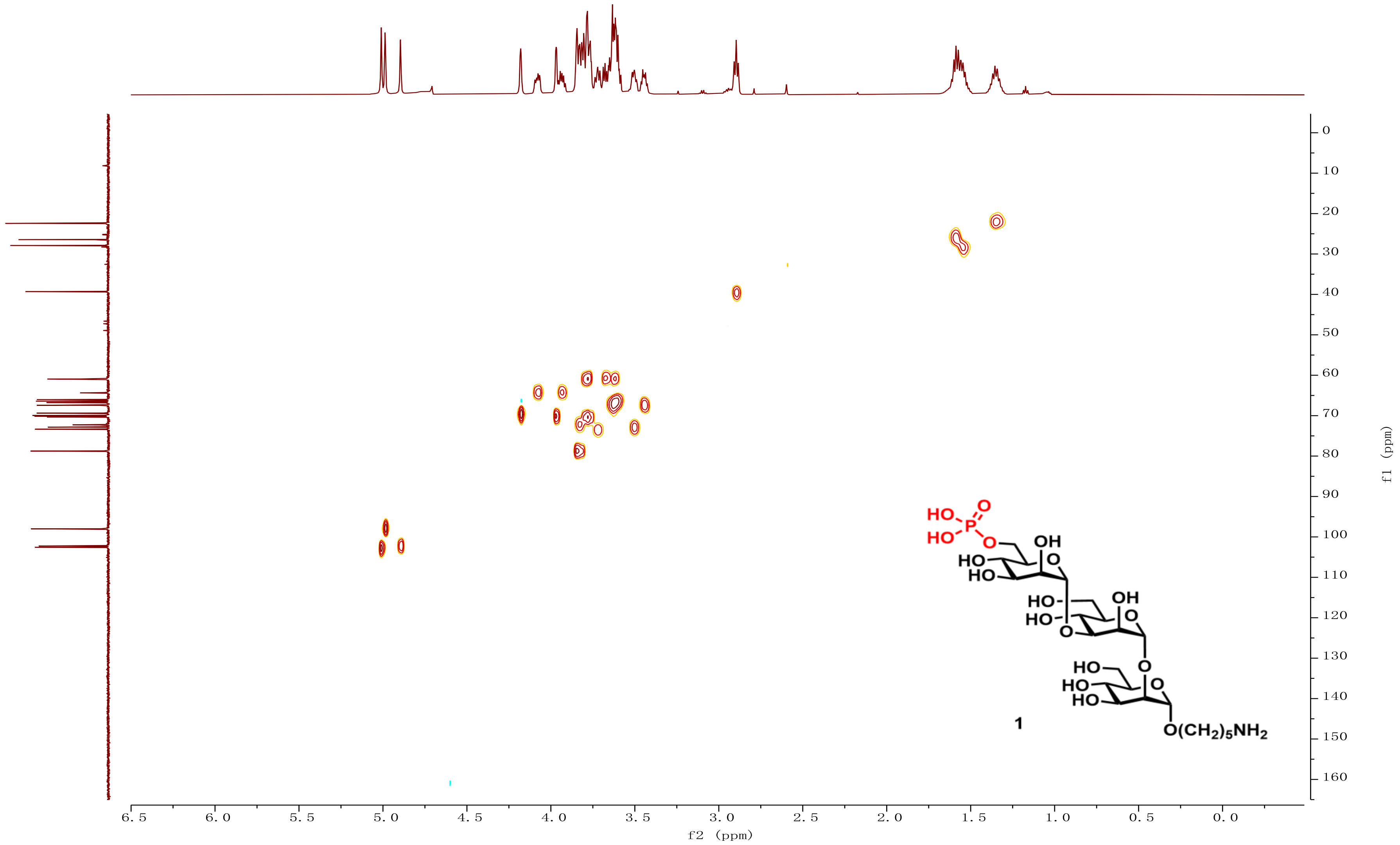

6.5 6.0 5.5 5.0 4.5 4.0 3.5 3.0 2.5 2.0 1.5 1.0 0.5 0.0
f2 (ppm)
0 10 20 30 40 50 60 70 80 90 100 110 120 130 140 150 160
f1 (ppm)
1
O(CH2)5NH2

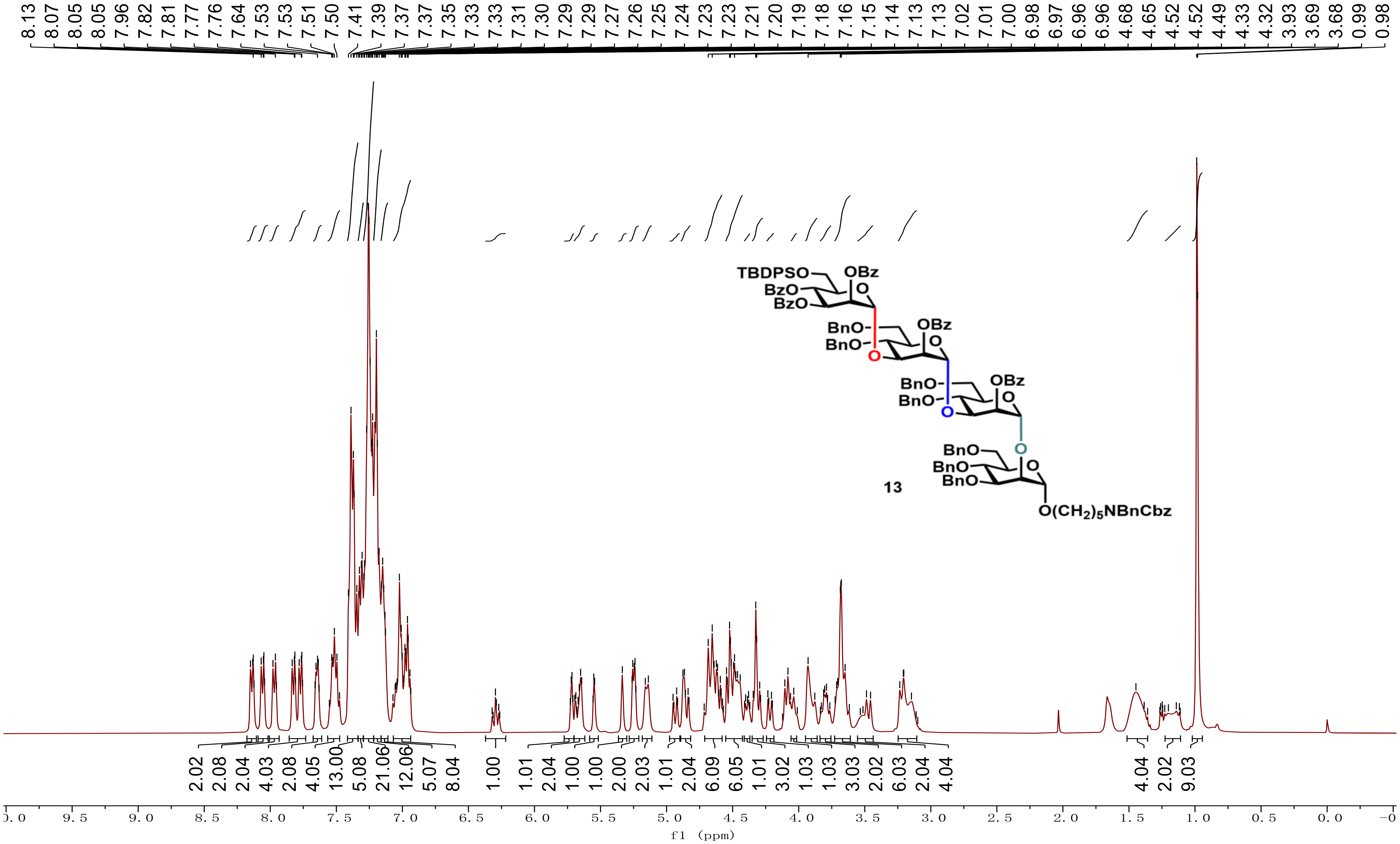
8.13 8.07 8.05 8.05 7.96 7.82 7.81 7.77 7.76 7.64 7.53 7.53 7.51 7.50 7.41 7.39 7.37 7.37 7.35 7.33 7.33 7.31 7.30 7.29 7.29 7.27 7.26 7.25 7.24 7.23 7.23 7.21 7.20 7.19 7.18 7.16 7.15 7.14 7.13 7.13 7.02 7.01 7.00 6.98 6.97 6.96 6.96 4.68 4.65 4.52 4.52 4.49 4.33 4.32 3.93 3.69 3.68 0.99 0.98
TBDPSO
OBz
BzO
BzO
BnO
BnO
OBz
BnO
BnO
OBz
BnO
BnO
BnO
13
O(CH2)5NBnCbz
2.02 2.08 2.04 4.03 2.08 4.05 13.00 5.08 21.06 12.06 5.07 8.04 1.00 1.01 2.04 1.00 1.00 2.00 2.03 1.01 2.04 6.09 6.05 1.01 3.02 1.03 1.03 3.03 2.02 6.03 2.04 4.04 4.04 2.02 9.03
10.0 9.5 9.0 8.5 8.0 7.5 7.0 6.5 6.0 5.5 5.0 4.5 4.0 3.5 3.0 2.5 2.0 1.5 1.0 0.5 0.0
f1 (ppm)

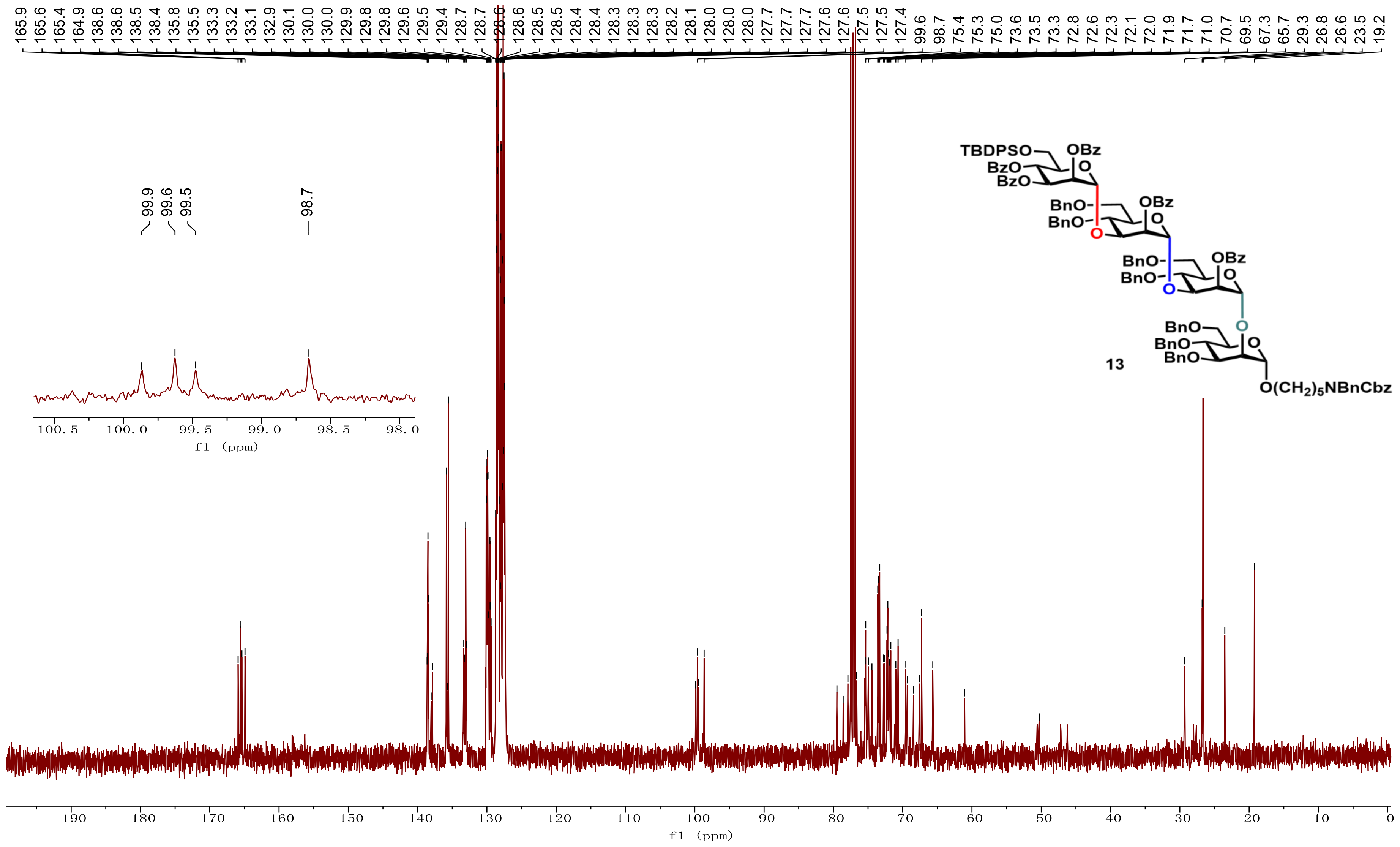
165.9 165.6 165.4 164.9 138.6 138.6 138.5 138.4 135.8 135.5 133.3 133.2 133.1 132.9 130.1 130.0 129.9 129.8 129.8 129.6 129.5 129.4 128.7 128.7 128.6 128.5 128.5 128.4 128.4 128.3 128.3 128.3 128.2 128.1 128.0 128.0 128.0 127.7 127.7 127.7 127.6 127.6 127.5 127.5 127.4 99.6 98.7 75.4 75.3 75.0 73.6 73.5 73.3 72.8 72.6 72.3 72.1 72.0 71.9 71.7 71.0 70.7 69.5 67.3 65.7 29.3 26.8 26.6 23.5 19.2
99.9 99.6 99.5 98.7
100.5 100.0 99.5 99.0 98.5 98.0
f1 (ppm)
TBDPSO
OBz
BzO
BzO
O
BnO
BnO
O
OBz
O
BnO
BnO
O
OBz
O
O
BnO
BnO
BnO
O
13
O(CH2)5NBnCbz
190 180 170 160 150 140 130 120 110 100 90 80 70 60 50 40 30 20 10 0
f1 (ppm)

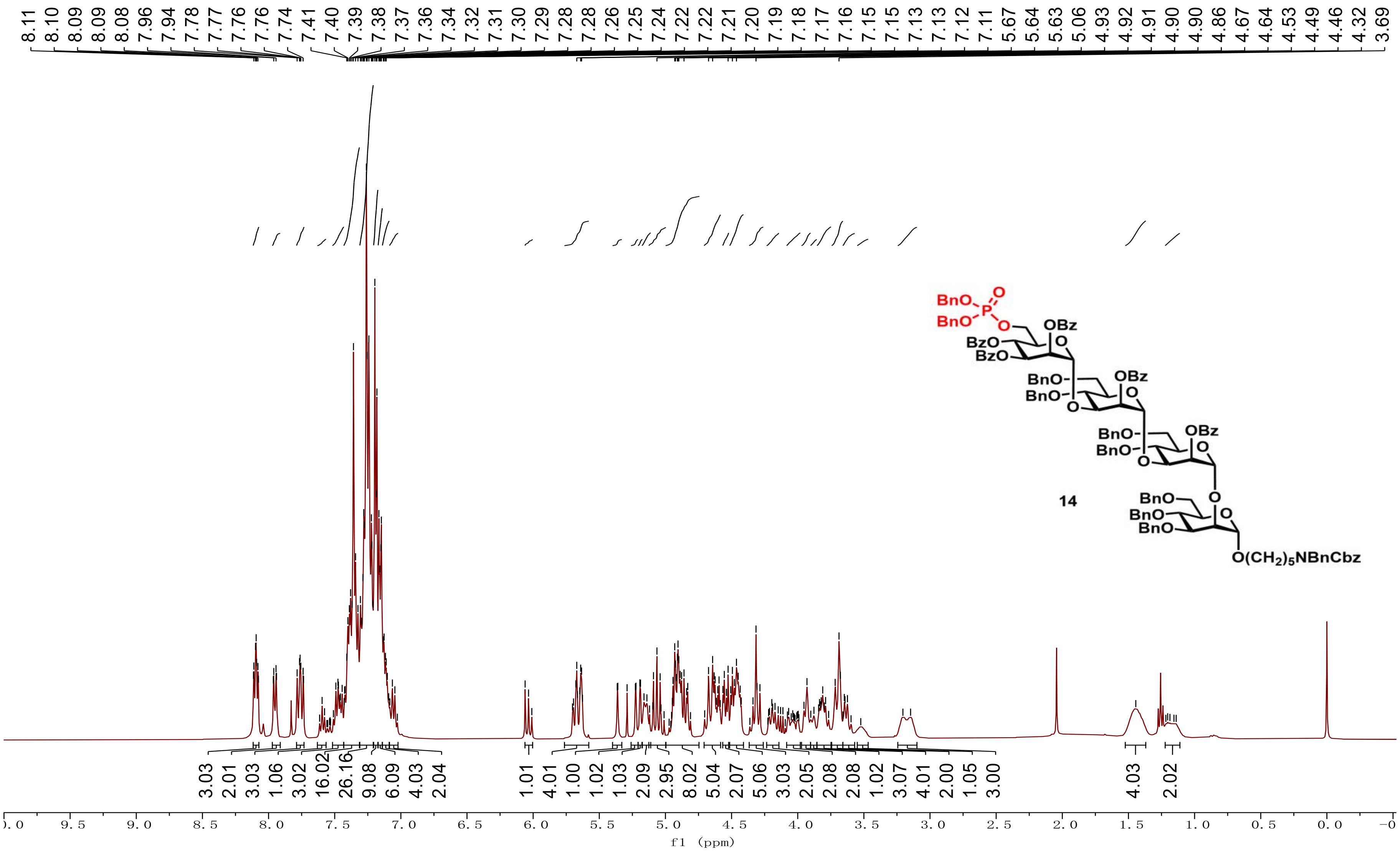

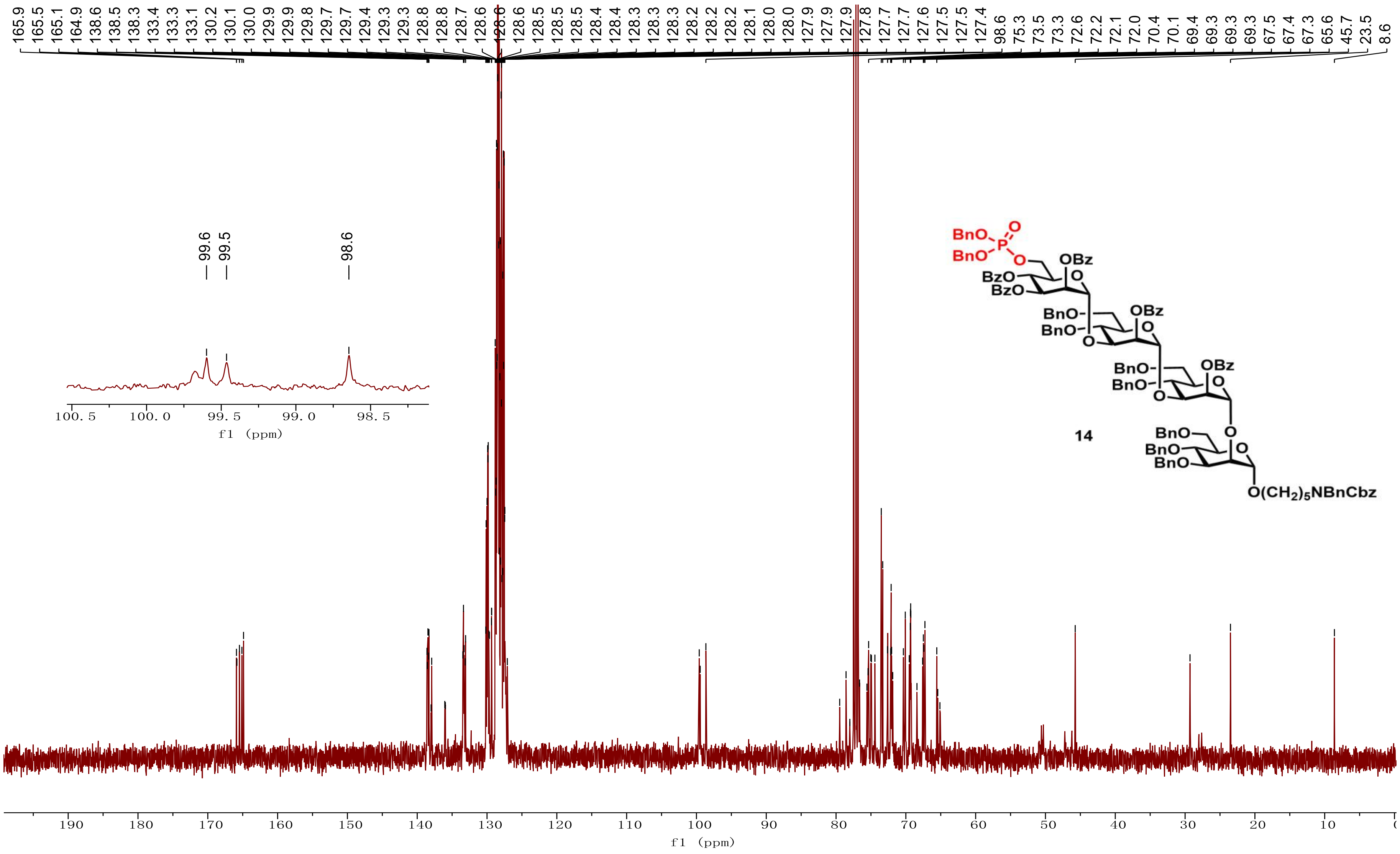

165.9 165.5 165.1 164.9 138.6 138.5 138.3 133.4 133.3 133.1 130.2 130.1 130.0 129.9 129.9 129.8 129.7 129.7 129.4 129.3 129.3 128.8 128.8 128.7 128.6 128.6 128.6 128.5 128.5 128.5 128.4 128.4 128.3 128.3 128.3 128.2 128.2 128.2 128.1 128.0 128.0 127.9 127.9 127.9 127.8 127.7 127.7 127.6 127.5 127.5 127.4 98.6 75.3 73.5 73.3 72.6 72.2 72.1 72.0 70.4 70.1 69.4 69.3 69.3 69.3 67.5 67.4 67.3 65.6 45.7 23.5 8.6
99.6
99.5
98.6
100.5 100.0 99.5 99.0 98.5
f1 (ppm)
BnO
BnO
P
O
O
OBz
BzO
BzO
O
BnO
BnO
OBz
O
O
BnO
BnO
OBz
O
O
14
BnO
BnO
BnO
O
O
O(CH2)5NBnCbz
190 180 170 160 150 140 130 120 110 100 90 80 70 60 50 40 30 20 10 0
f1 (ppm)

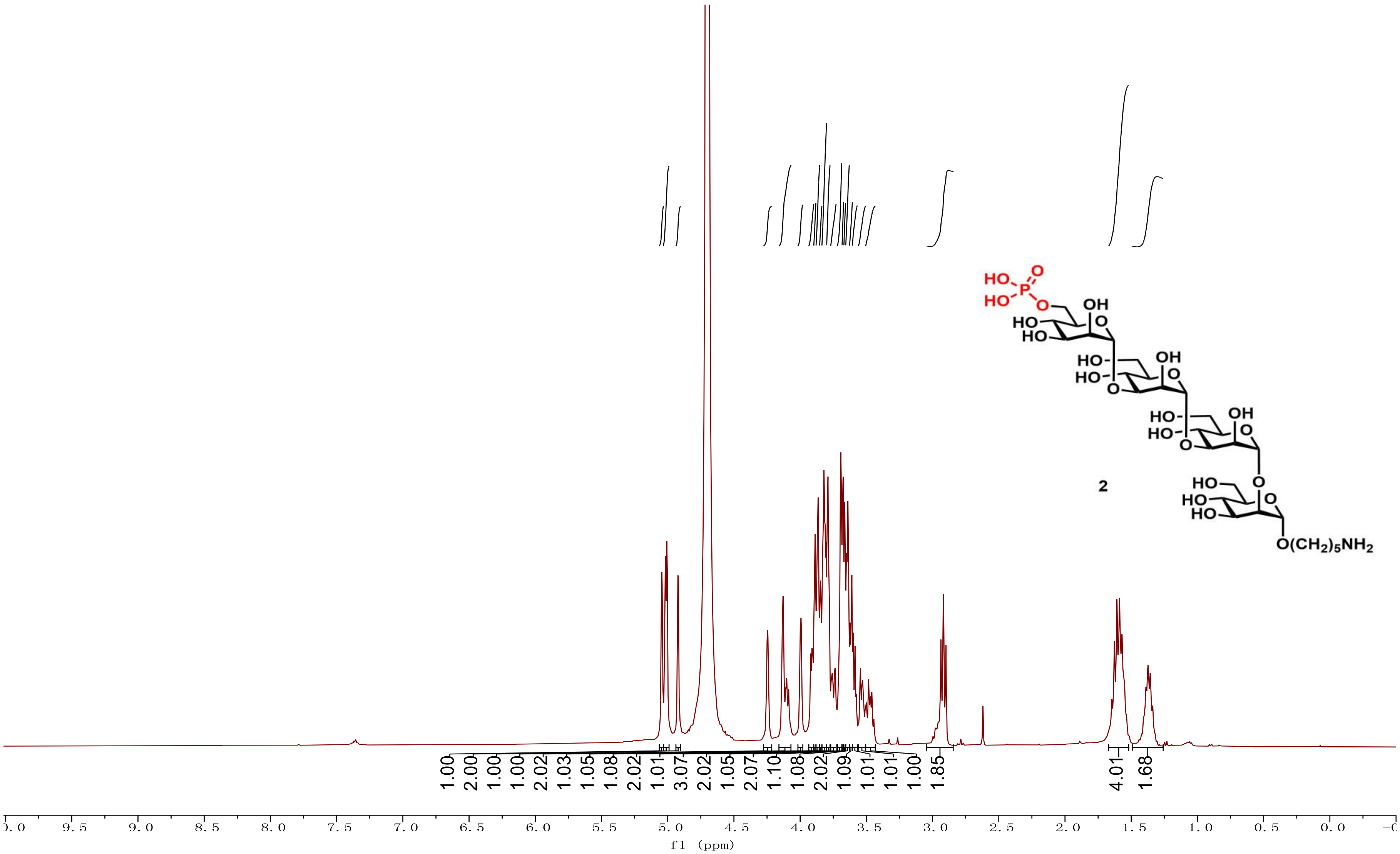
1.00
2.00
1.00
1.00
2.02
1.03
1.05
1.08
2.02
1.01
3.07
2.02
1.05
2.07
1.10
1.08
2.02
1.09
1.01
1.01
1.00
1.85
4.01
1.68
f1 (ppm)
O(CH_2)$_5$$NH_2$
2

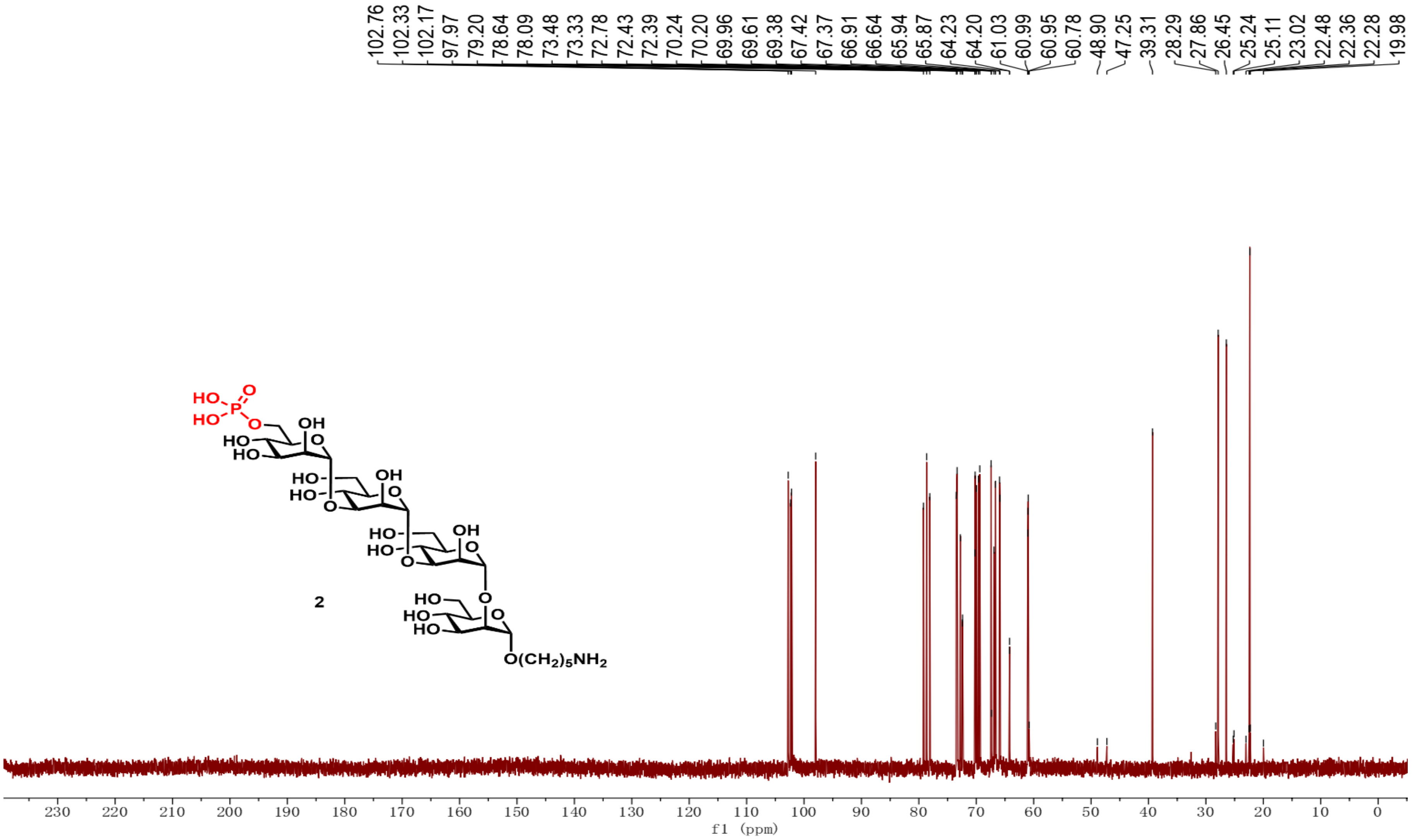
102.76
102.33
102.17
97.97
79.20
78.64
78.09
73.48
73.33
72.78
72.43
72.39
70.24
70.20
69.96
69.61
69.38
67.42
67.37
66.91
66.64
65.94
65.87
64.23
64.20
61.03
60.99
60.95
60.78
48.90
47.25
39.31
28.29
27.86
26.45
25.24
25.11
23.02
22.48
22.36
22.28
19.98
2
O(CH2)5NH2
230 220 210 200 190 180 170 160 150 140 130 120 110 100 90 80 70 60 50 40 30 20 10 0
f1 (ppm)

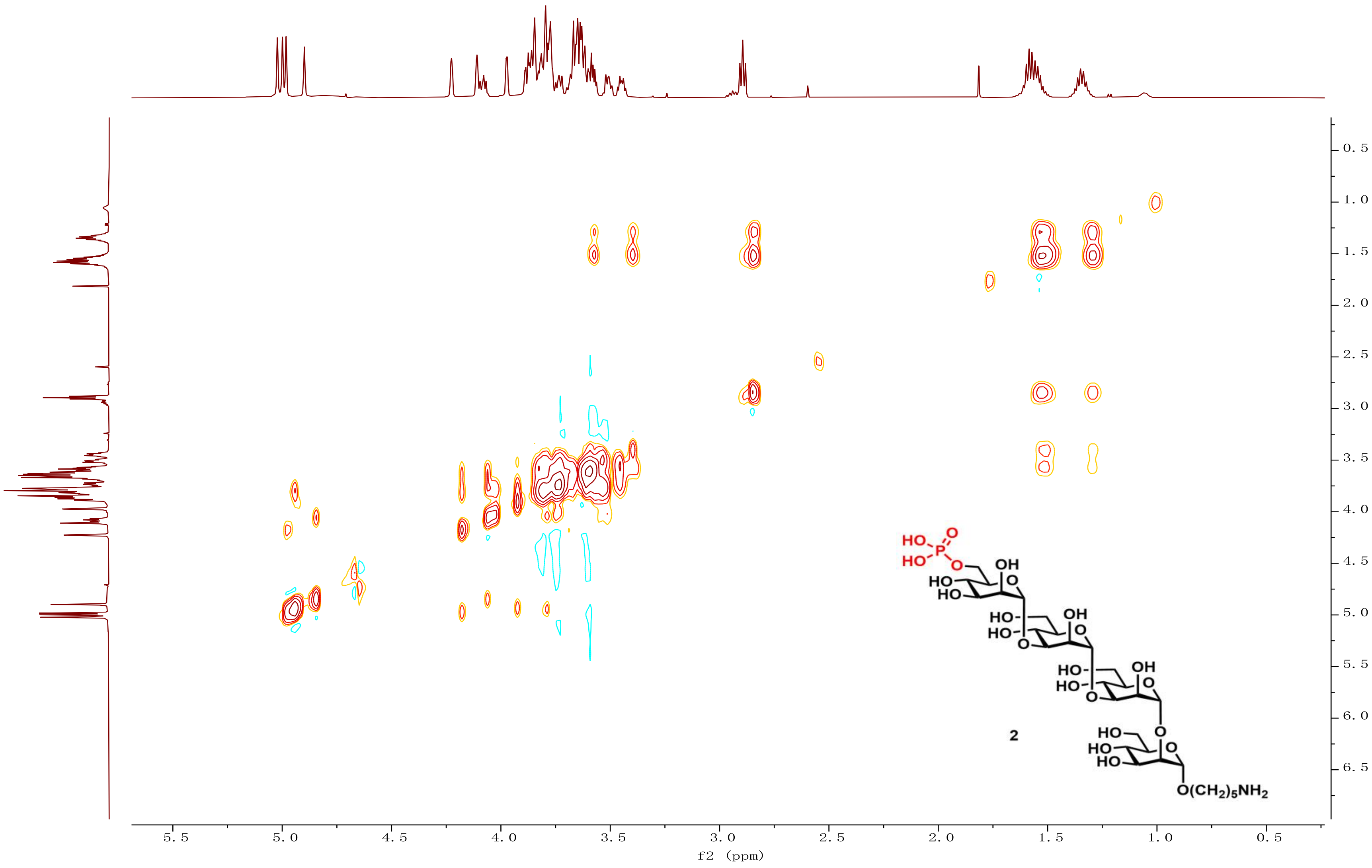

5.5
5.0
4.5
4.0
3.5
3.0
2.5
2.0
1.5
1.0
0.5
f2 (ppm)
0.5
1.0
1.5
2.0
2.5
3.0
3.5
4.0
4.5
5.0
5.5
6.0
6.5
f1 (ppm)
HO
HO
P
O
O
OH
HO
HO
O
HO
HO
OH
O
O
HO
HO
OH
O
O
O
HO
HO
HO
O
O(CH2)5NH2

2

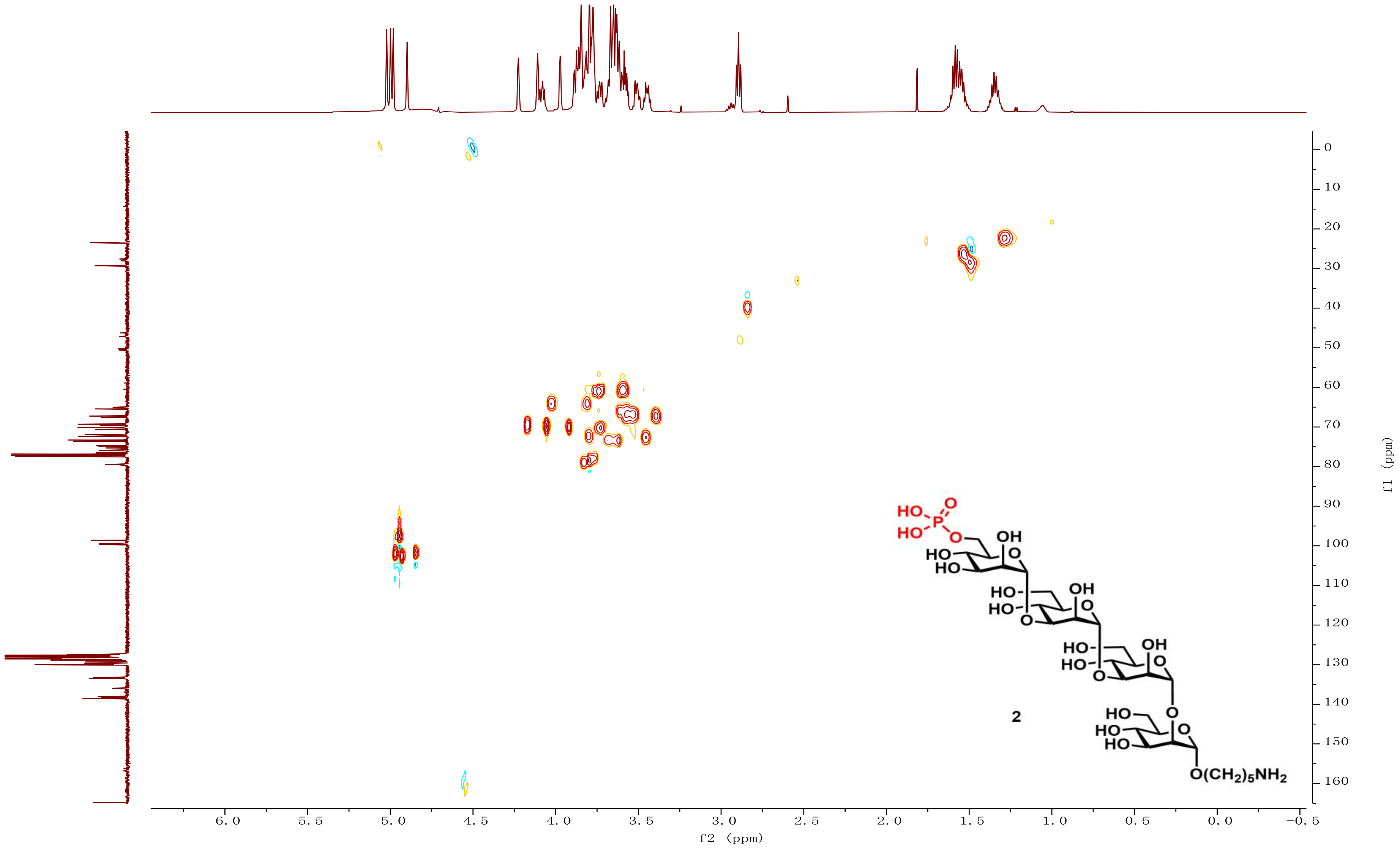
6.0
5.5
5.0
4.5
4.0
3.5
3.0
2.5
2.0
1.5
1.0
0.5
0.0
-0.5
f2 (ppm)
0
10
20
30
40
50
60
70
80
90
100
110
120
130
140
150
160
f1 (ppm)
2
O(CH2)5NH2

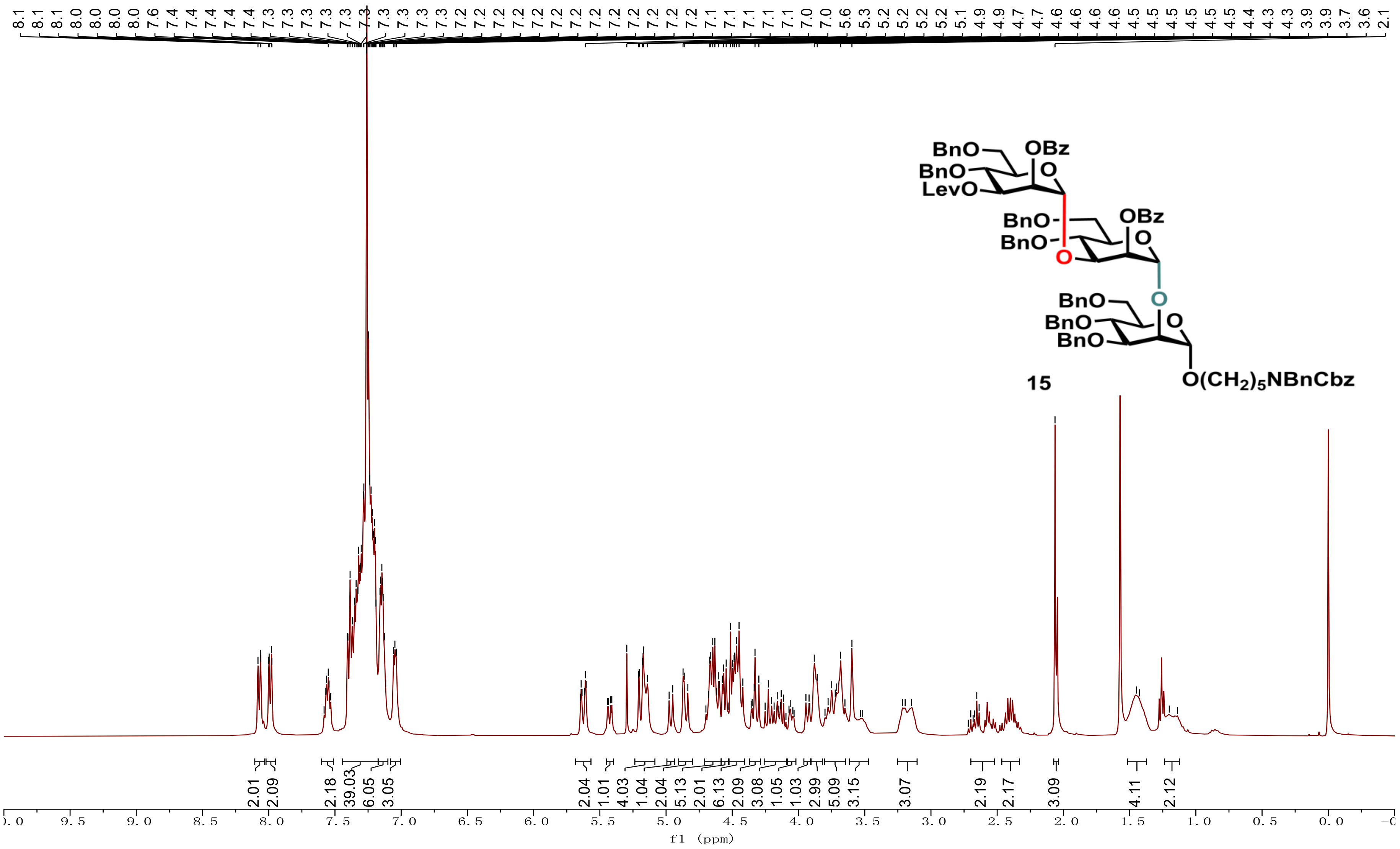
BnO
OBz
BnO
LevO
O
BnO
OBz
BnO
O
O
O
BnO
BnO
O
BnO
O(CH2)5NBnCbz
15
f1 (ppm)

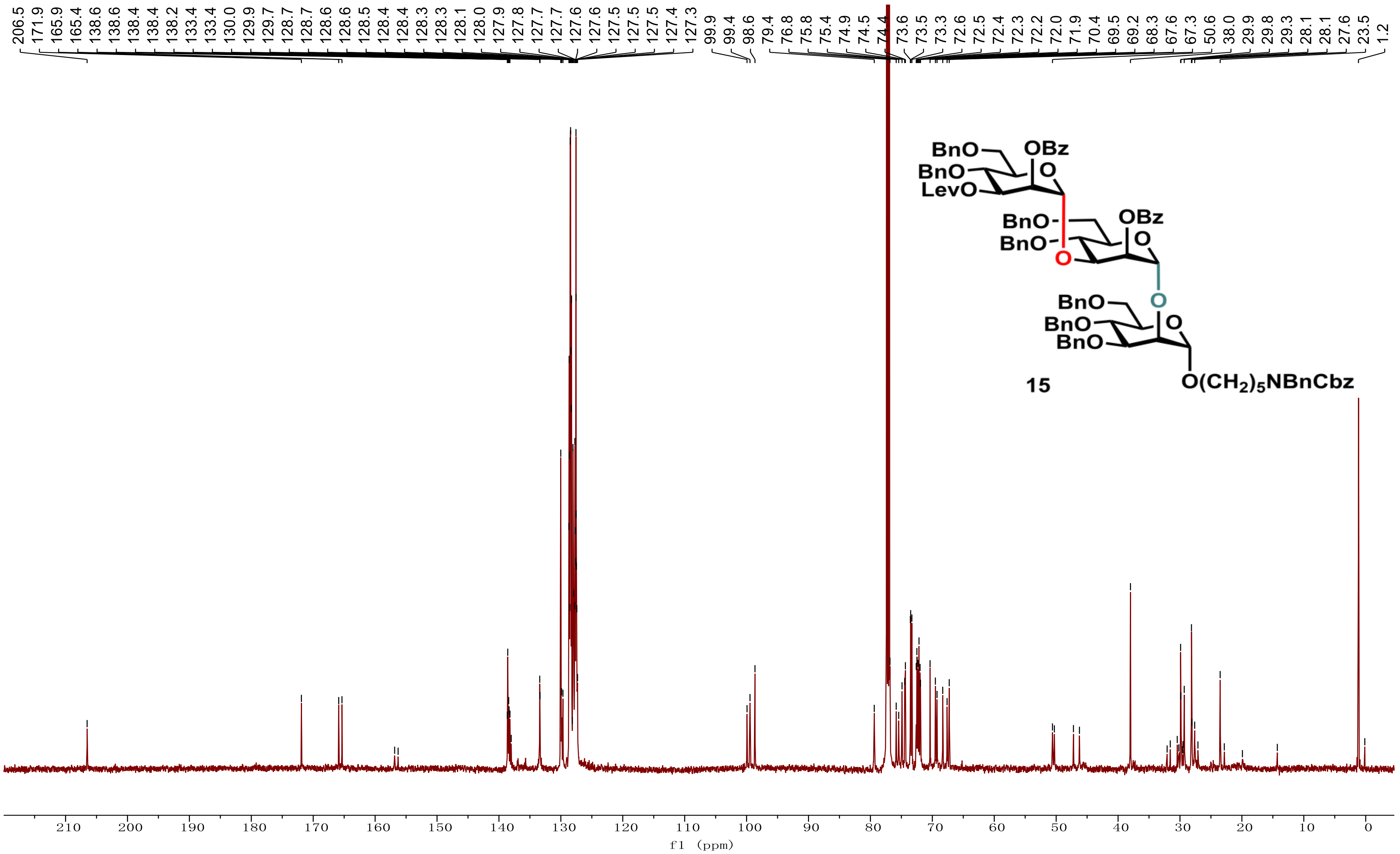
206.5
171.9
165.9
165.4
138.6
138.6
138.4
138.4
138.2
133.4
133.4
130.0
129.9
129.7
128.7
128.7
128.6
128.6
128.5
128.4
128.4
128.3
128.3
128.1
128.0
127.9
127.8
127.7
127.7
127.6
127.6
127.5
127.5
127.5
127.4
127.3
99.9
99.4
98.6
79.4
76.8
75.8
75.4
74.9
74.5
74.4
73.6
73.5
73.3
72.6
72.5
72.4
72.3
72.2
72.0
71.9
70.4
69.5
69.2
68.3
67.6
67.3
50.6
38.0
29.9
29.8
29.3
28.1
28.1
27.6
23.5
1.2
BnO
OBz
BnO
O
LevO
BnO
OBz
BnO
O
O
O
BnO
BnO
O
BnO
15
O(CH2)5NBnCbz
210 200 190 180 170 160 150 140 130 120 110 100 90 80 70 60 50 40 30 20 10 0
f1 (ppm)

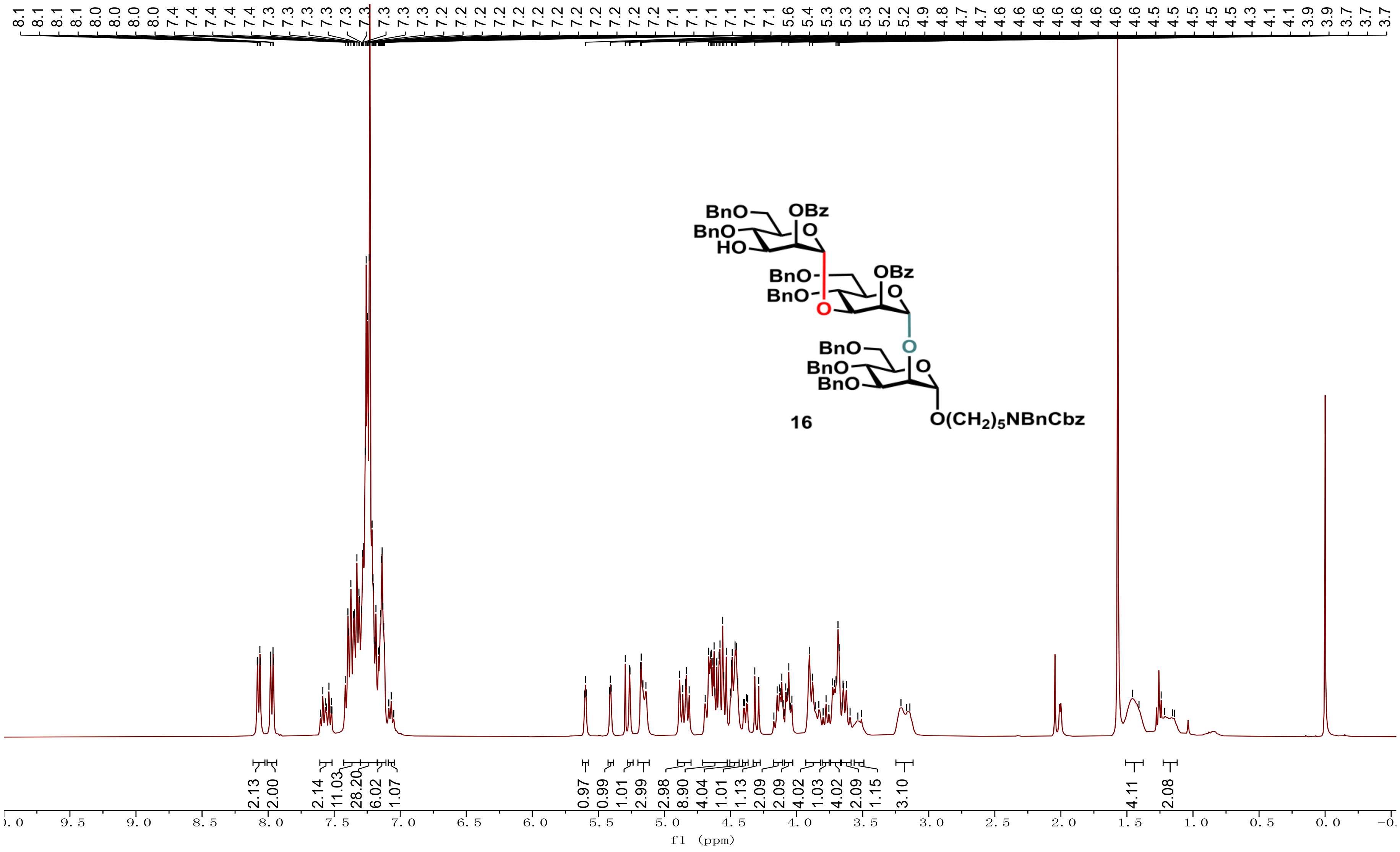
BnO
OBz
BnO
HO
BnO
OBz
BnO
BnO
BnO
BnO
O(CH2)5NBnCbz
16
f1 (ppm)

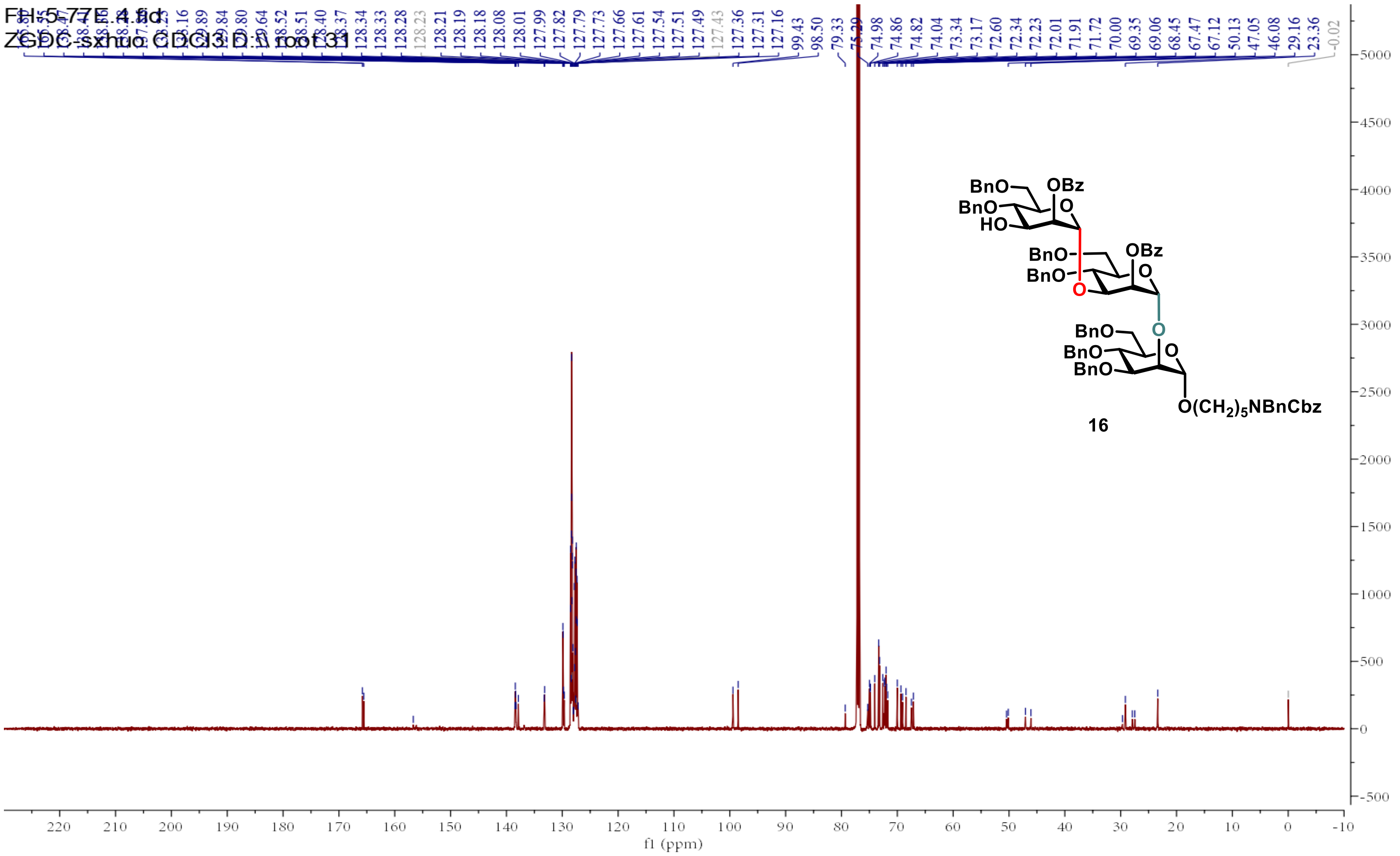
128.34
128.33
128.28
128.23
128.21
128.19
128.18
128.08
128.01
127.99
127.82
127.79
127.73
127.66
127.61
127.54
127.51
127.49
127.43
127.36
127.31
127.16
99.43
98.50
79.33
74.98
74.86
74.82
74.04
73.34
73.17
72.60
72.34
72.23
72.01
71.91
71.72
70.00
69.35
69.06
68.45
67.47
67.12
50.13
47.05
46.08
29.16
23.36
-0.02
BnO
BnO
HO
OBz
O
BnO
BnO
O
OBz
O
O
BnO
BnO
BnO
O
O(CH2)5NBnCbz
16
f1 (ppm)

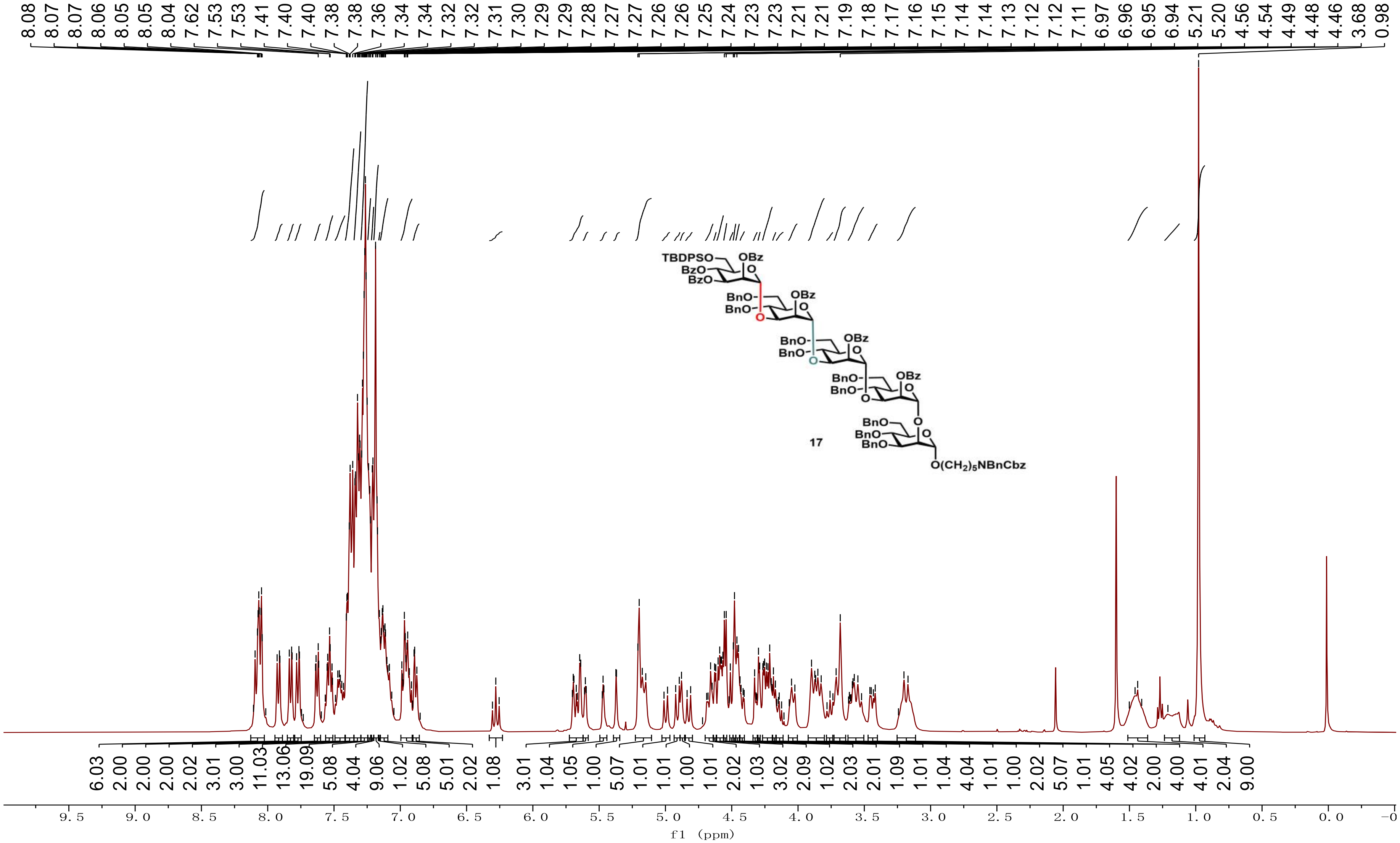
8.08 8.07 8.07 8.06 8.05 8.05 8.04 7.62 7.53 7.53 7.41 7.40 7.40 7.38 7.38 7.36 7.34 7.34 7.32 7.32 7.31 7.30 7.29 7.29 7.28 7.27 7.27 7.26 7.26 7.25 7.24 7.23 7.23 7.21 7.21 7.21 7.19 7.18 7.17 7.16 7.15 7.14 7.14 7.13 7.12 7.12 7.11 6.97 6.96 6.95 6.94 5.21 5.20 5.20 4.56 4.54 4.49 4.48 4.46 3.68 0.98
TBDPSO
OBz
BzO
BzO
BnO
BnO
OBz
BnO
BnO
OBz
BnO
BnO
OBz
BnO
BnO
BnO
O(CH2)5NBnCbz
17
6.03 2.00 2.00 2.00 2.02 3.01 3.00 11.03 13.06 19.09 5.08 4.04 9.06 1.02 5.08 5.01 2.02 1.08 3.01 1.04 1.05 1.00 5.07 1.01 1.01 1.00 1.01 2.02 1.03 3.02 2.09 1.02 2.03 2.01 1.09 1.01 1.04 4.04 1.01 1.00 2.02 5.07 1.01 4.05 4.02 2.00 4.00 4.01 2.04 9.00
9.5 9.0 8.5 8.0 7.5 7.0 6.5 6.0 5.5 5.0 4.5 4.0 3.5 3.0 2.5 2.0 1.5 1.0 0.5 0.0 -0
f1 (ppm)

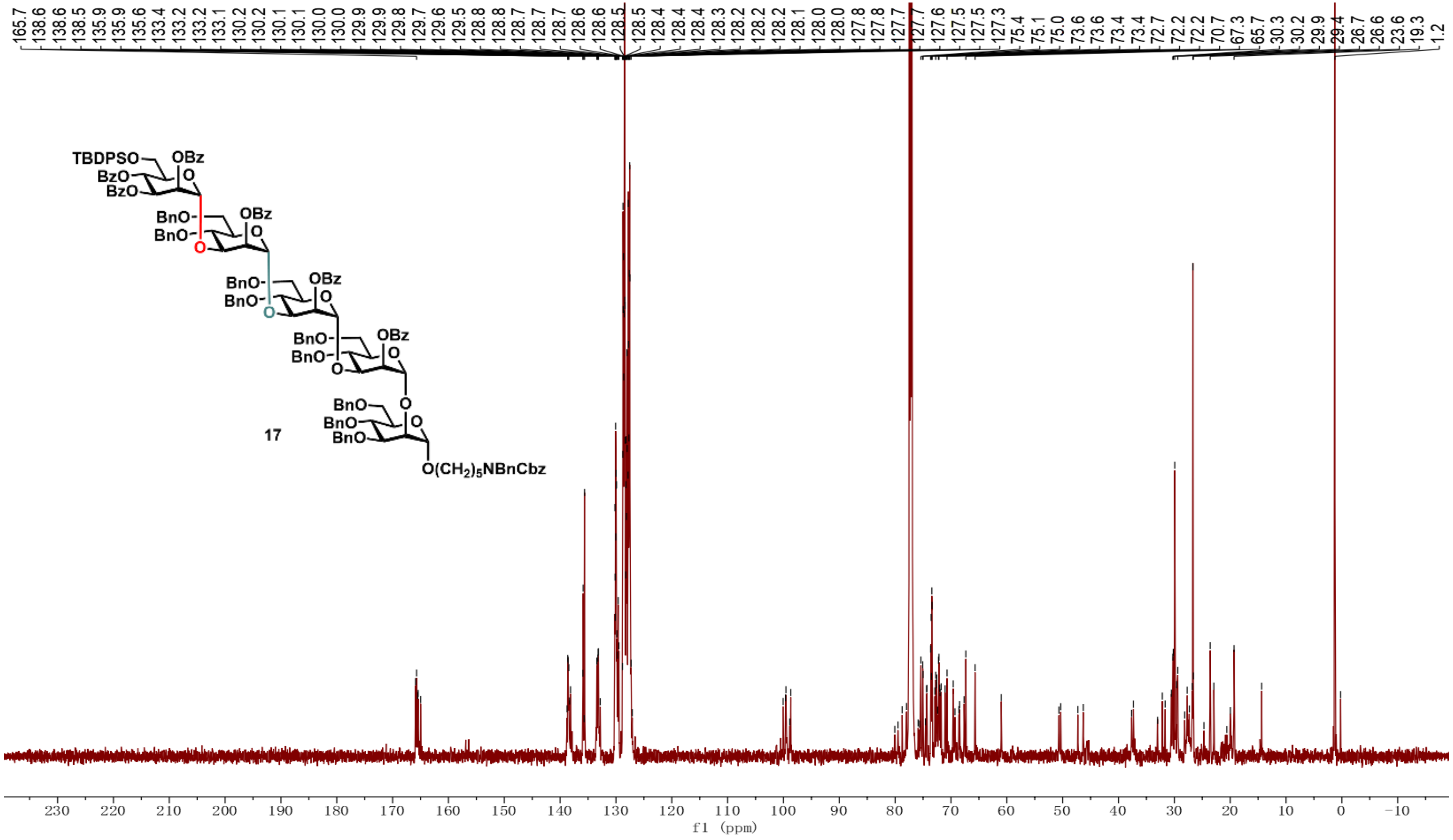
165.7
138.6
138.6
138.5
135.9
135.9
135.6
133.4
133.2
133.2
133.1
130.2
130.2
130.1
130.1
130.0
130.0
129.9
129.9
129.8
129.7
129.6
129.5
128.8
128.8
128.7
128.7
128.7
128.6
128.6
128.5
128.5
128.4
128.4
128.4
128.3
128.2
128.2
128.2
128.1
128.0
128.0
127.8
127.8
127.7
127.7
127.6
127.5
127.5
127.3
75.4
75.1
75.0
73.6
73.6
73.4
73.4
72.7
72.2
72.2
70.7
67.3
65.7
30.3
30.2
29.9
29.4
26.7
26.6
23.6
19.3
1.2
TBDPSO
OBz
BzO
BzO
BnO
BnO
BnO
BnO
BnO
BnO
BnO
BnO
BnO
17
O(CH2)5NBnCbz
230 220 210 200 190 180 170 160 150 140 130 120 110 100 90 80 70 60 50 40 30 20 10 0 −10
f1 (ppm)

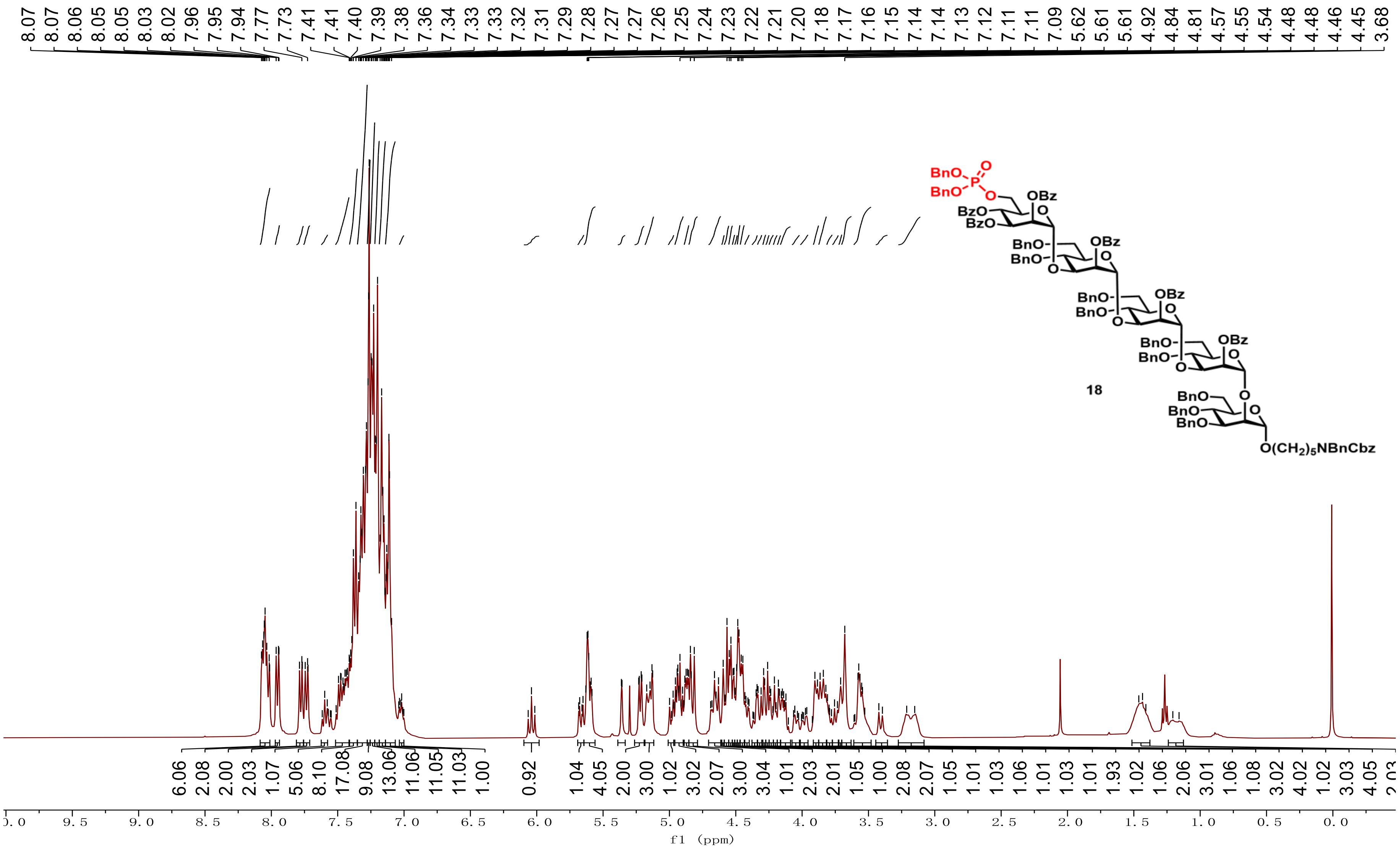
8.07 8.07 8.06 8.05 8.05 8.03 8.02 7.96 7.95 7.94 7.77 7.73 7.41 7.41 7.40 7.39 7.38 7.36 7.34 7.33 7.33 7.32 7.31 7.29 7.28 7.27 7.27 7.26 7.25 7.24 7.23 7.22 7.21 7.20 7.18 7.17 7.16 7.15 7.14 7.14 7.13 7.12 7.11 7.11 7.09 5.62 5.61 5.61 4.92 4.84 4.81 4.57 4.55 4.54 4.48 4.48 4.46 4.45 3.68
BnO
BnO
P
O
O
OBz
BzO
BzO
O
BnO
BnO
OBz
O
O
BnO
BnO
OBz
O
O
BnO
BnO
OBz
O
O
18
BnO
BnO
BnO
O
O
O(CH2)5NBnCbz
6.06 2.08 2.00 2.03 1.07 5.06 8.10 17.08 9.08 13.06 11.06 11.05 11.03 1.00 0.92 1.04 4.05 2.00 3.00 1.02 3.02 2.07 3.00 3.04 1.01 2.03 2.01 1.05 1.00 2.08 2.07 1.05 1.01 1.03 1.06 1.01 1.03 1.01 1.93 1.02 1.06 2.06 3.01 1.06 1.08 3.02 4.02 1.02 3.03 4.05
9.5 9.0 8.5 8.0 7.5 7.0 6.5 6.0 5.5 5.0 4.5 4.0 3.5 3.0 2.5 2.0 1.5 1.0 0.5 0.0
f1 (ppm)

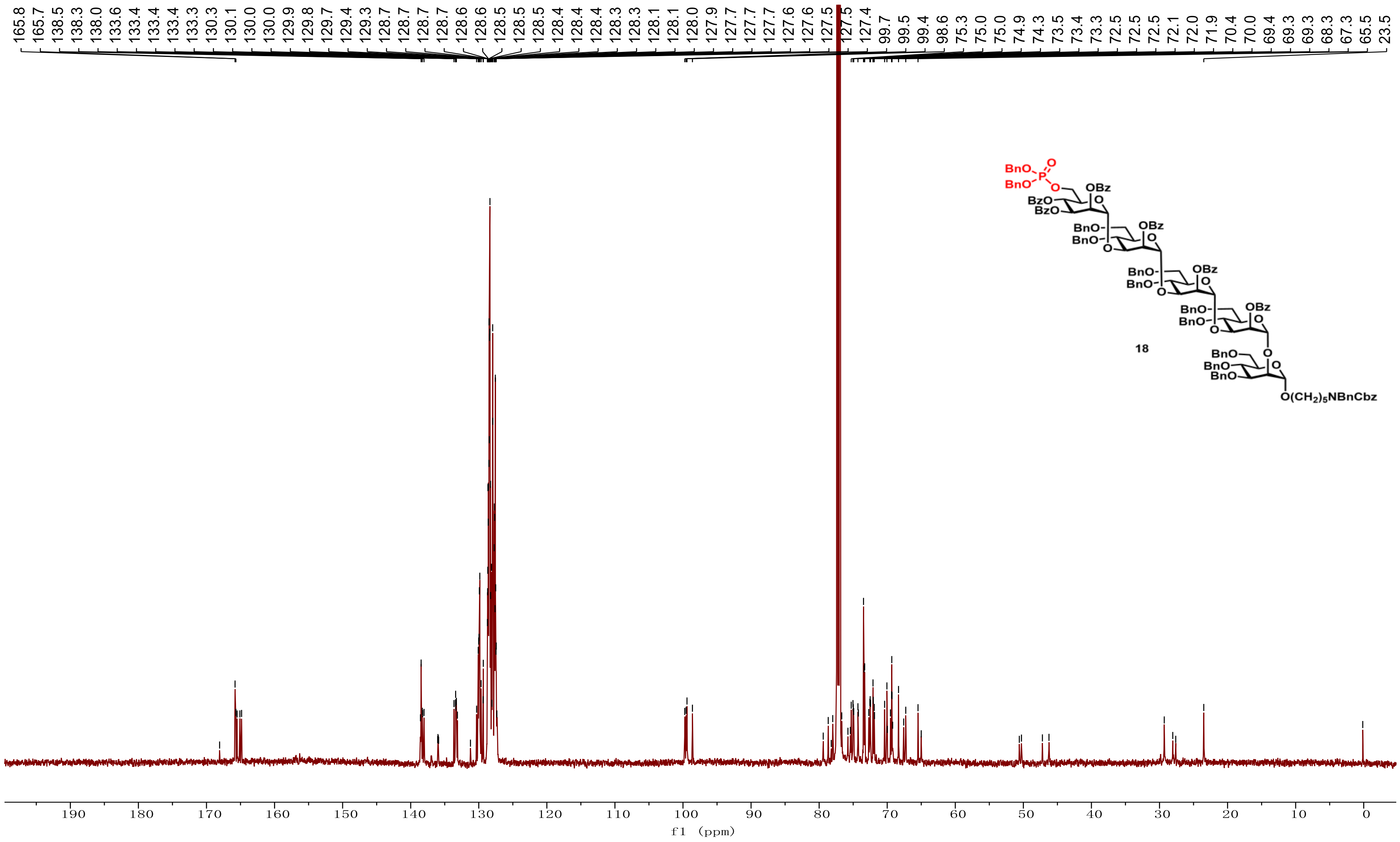
165.8
165.7
138.5
138.3
138.0
133.6
133.4
133.4
133.4
133.3
130.3
130.1
130.0
130.0
129.9
129.8
129.7
129.4
129.3
128.7
128.7
128.7
128.7
128.6
128.6
128.5
128.5
128.5
128.4
128.4
128.4
128.3
128.3
128.1
128.1
128.0
127.9
127.7
127.7
127.7
127.6
127.6
127.5
127.5
127.4
99.7
99.5
99.4
98.6
75.3
75.0
75.0
74.9
74.3
73.5
73.4
73.3
72.5
72.5
72.5
72.1
72.0
71.9
70.4
70.0
69.4
69.3
69.3
68.3
67.3
65.5
23.5
BnO
BnO
P
O
O
OBz
BzO
BzO
BnO
BnO
OBz
18
O(CH2)5NBnCbz
190 180 170 160 150 140 130 120 110 100 90 80 70 60 50 40 30 20 10 0
f1 (ppm)

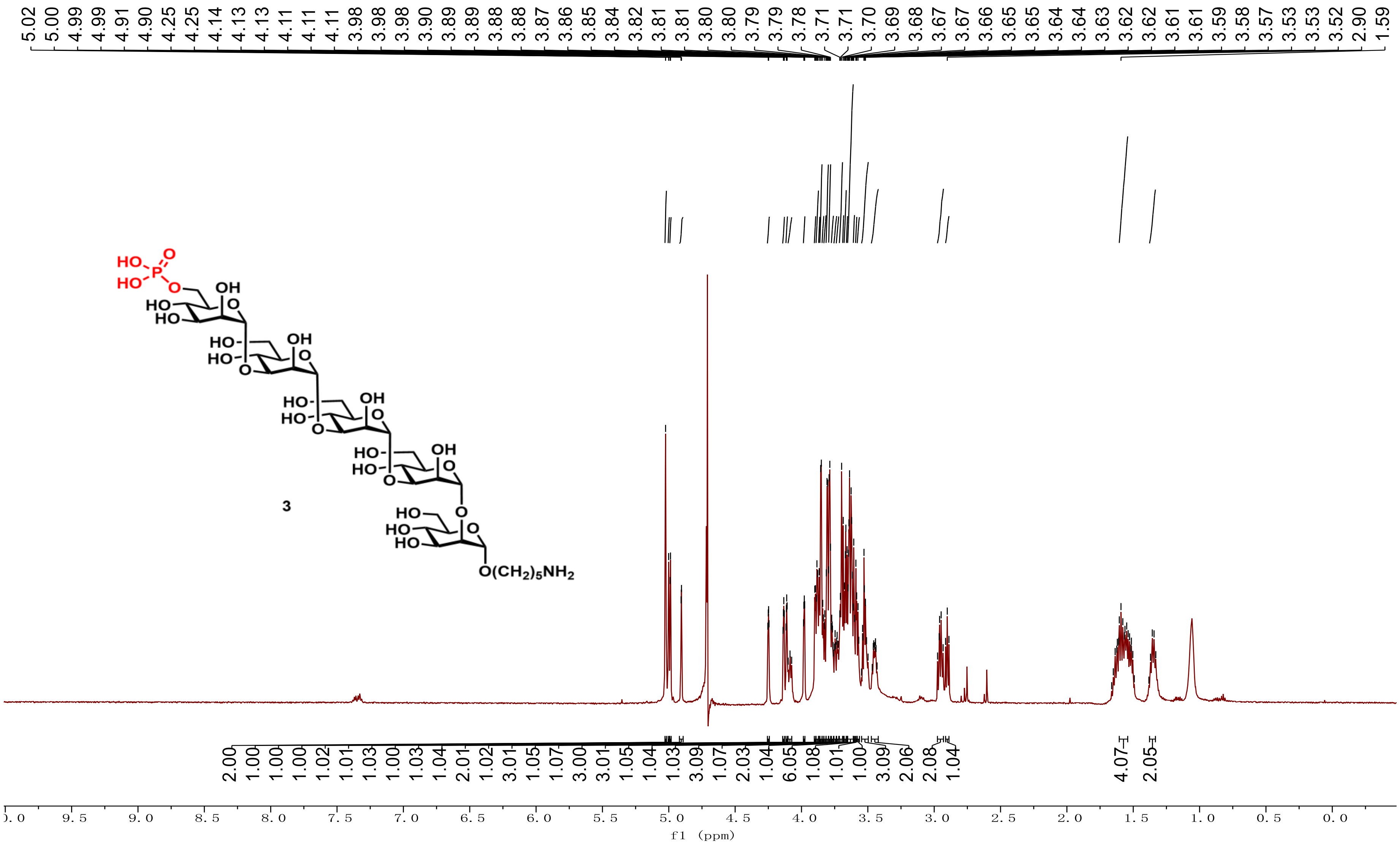

5.02 5.00 4.99 4.99 4.91 4.90 4.25 4.25 4.14 4.13 4.13 4.11 4.11 4.11 3.98 3.98 3.98 3.90 3.89 3.89 3.88 3.88 3.87 3.86 3.85 3.84 3.82 3.81 3.81 3.80 3.80 3.79 3.79 3.78 3.71 3.71 3.70 3.69 3.68 3.67 3.67 3.66 3.65 3.65 3.64 3.64 3.63 3.62 3.62 3.61 3.61 3.59 3.58 3.57 3.53 3.53 3.52 2.90 1.59
3
2.00 1.00 1.00 1.00 1.02 1.01 1.03 1.00 1.03 1.04 2.01 1.02 3.01 1.05 1.07 3.00 3.01 1.05 1.04 1.03 3.09 1.07 2.03 1.04 6.05 1.08 1.01 1.00 3.09 2.06 2.08 1.04 4.07 2.05
f1 (ppm)

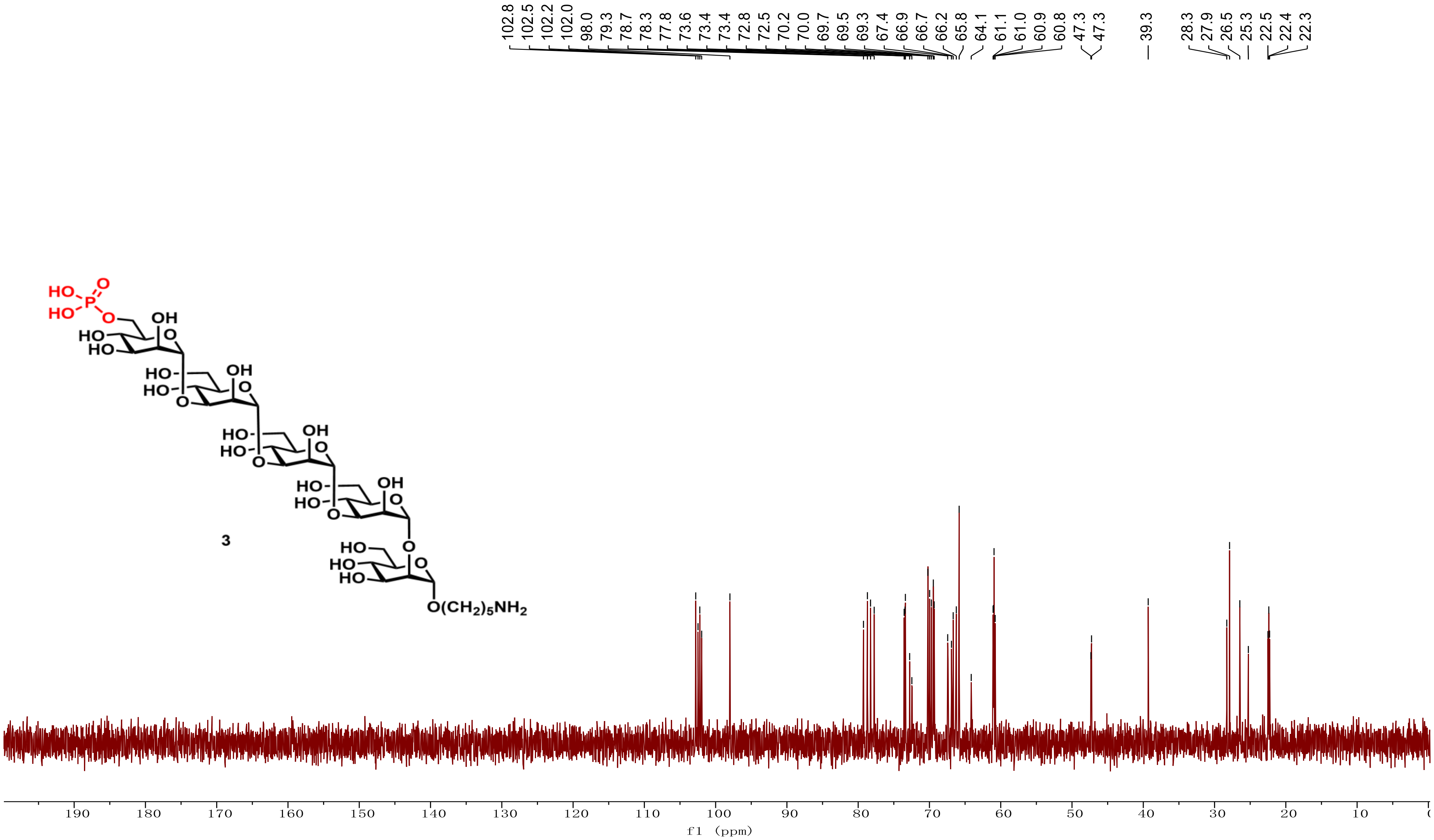
102.8
102.5
102.2
102.0
98.0
79.3
78.7
78.3
77.8
73.6
73.4
73.4
72.8
72.5
70.2
70.0
69.7
69.5
69.3
67.4
66.9
66.7
66.2
65.8
64.1
61.1
61.0
60.9
60.8
47.3
47.3
39.3
28.3
27.9
26.5
25.3
22.5
22.4
22.3
190 180 170 160 150 140 130 120 110 100 90 80 70 60 50 40 30 20 10 0
f1 (ppm)

3

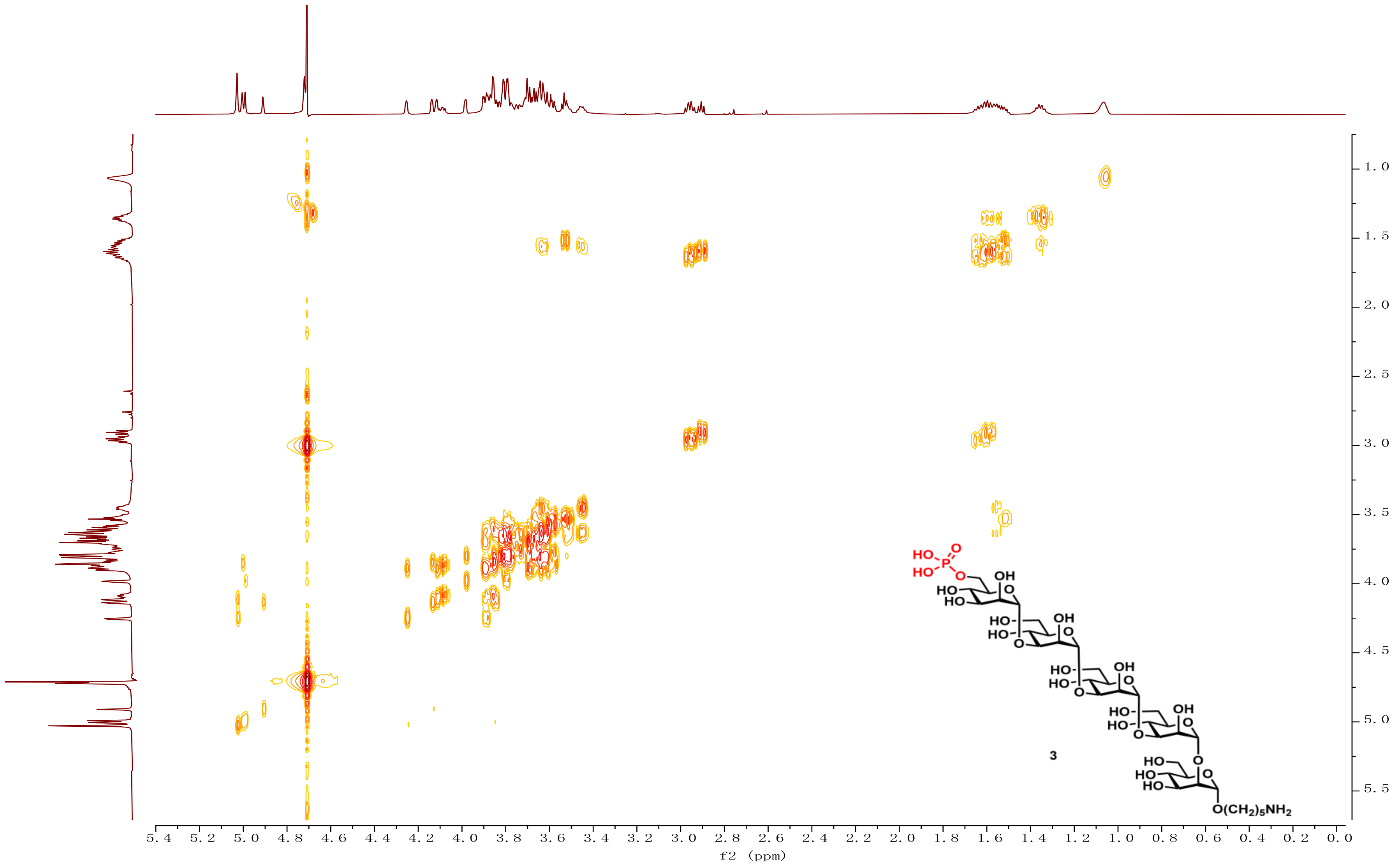
f2 (ppm)
f1 (ppm)
HO
HO
P
O
O
OH
O
3
O(CH2)5NH2

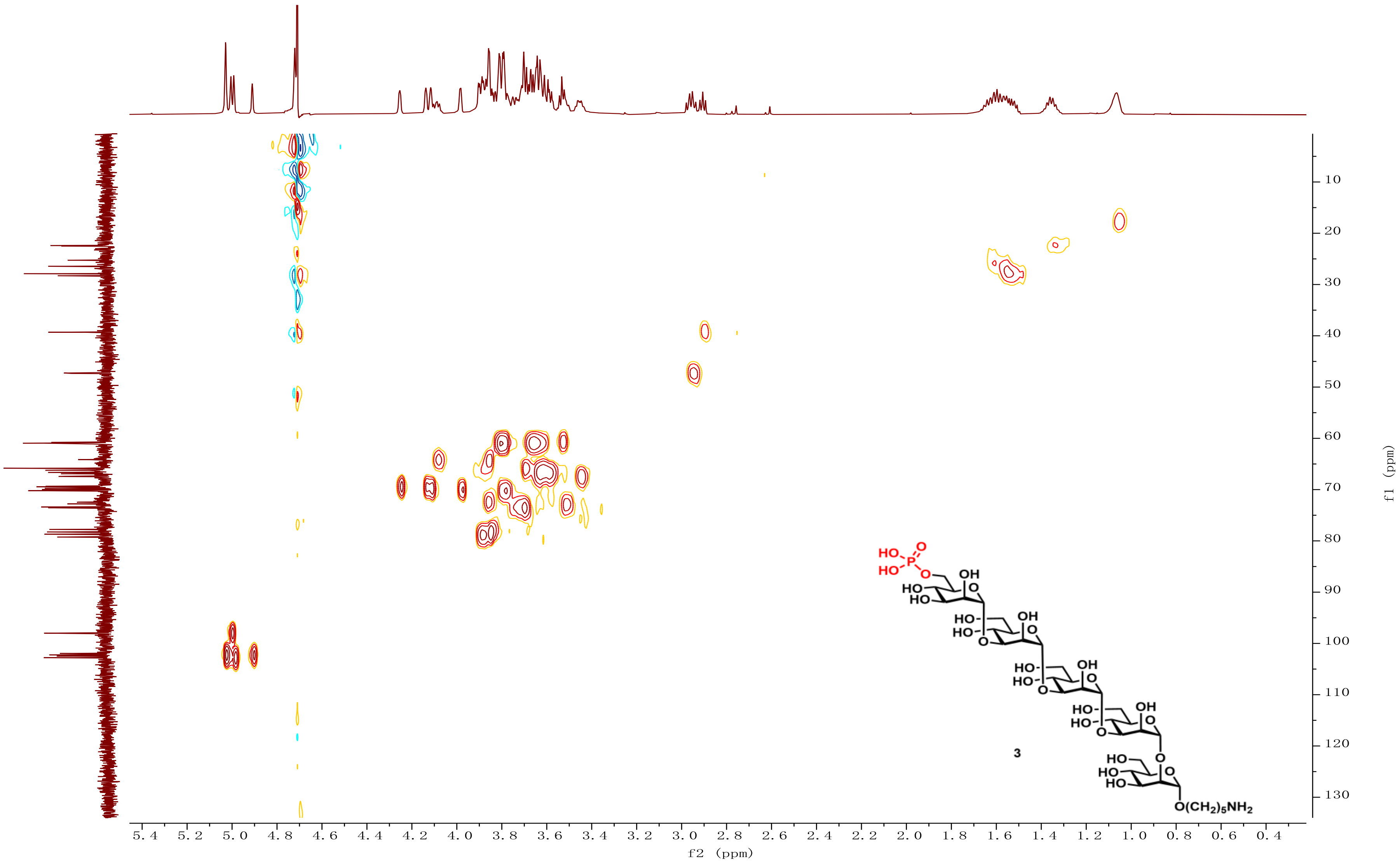
f2 (ppm)
f1 (ppm)
HO
HO
P
O
OH
3
O(CH2)5NH2

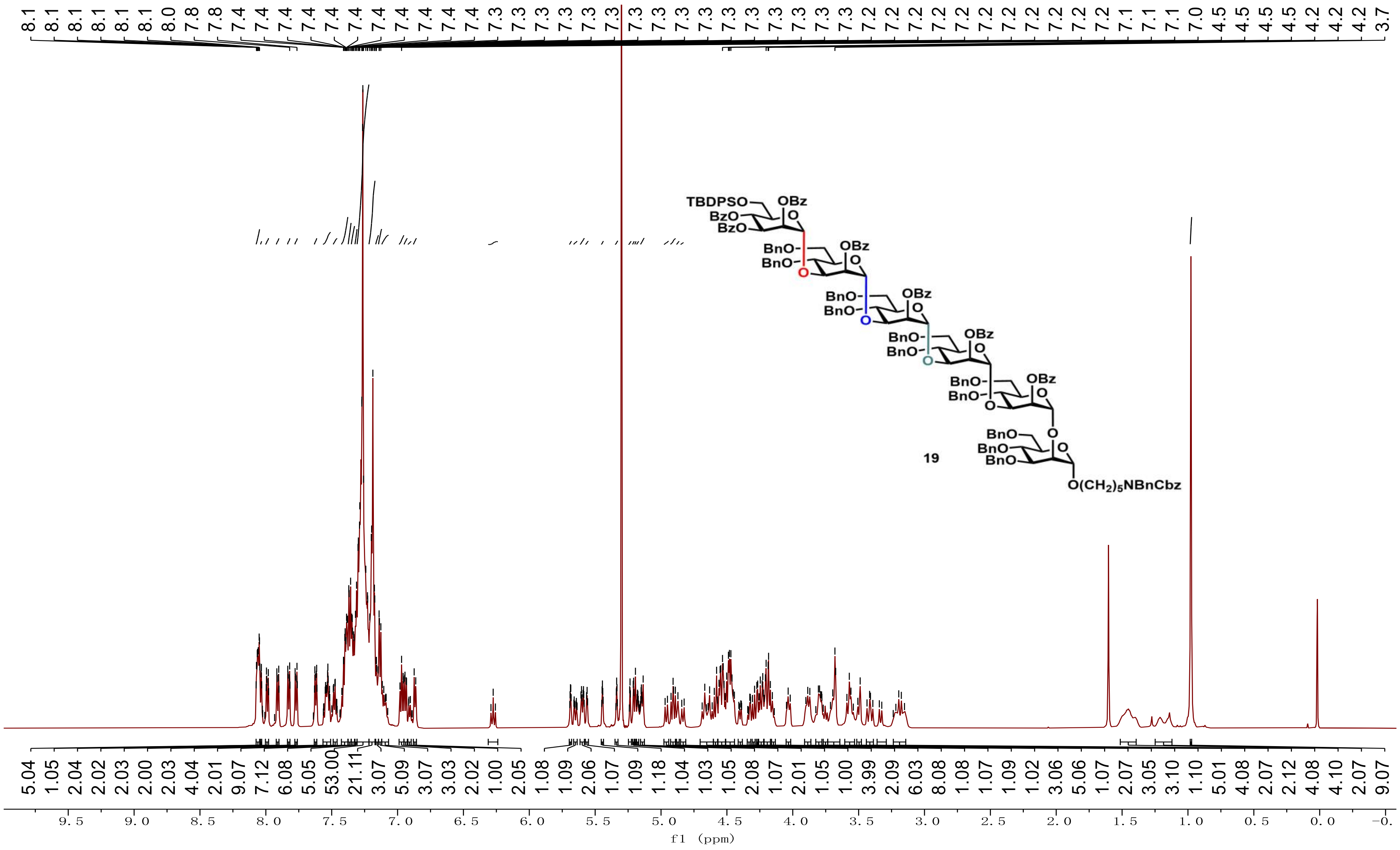
TBDPSO
OBz
BzO
BzO
BnO
BnO
OBz
BnO
BnO
OBz
BnO
BnO
OBz
BnO
BnO
OBz
BnO
BnO
BnO
O(CH2)5NBnCbz
19
f1 (ppm)

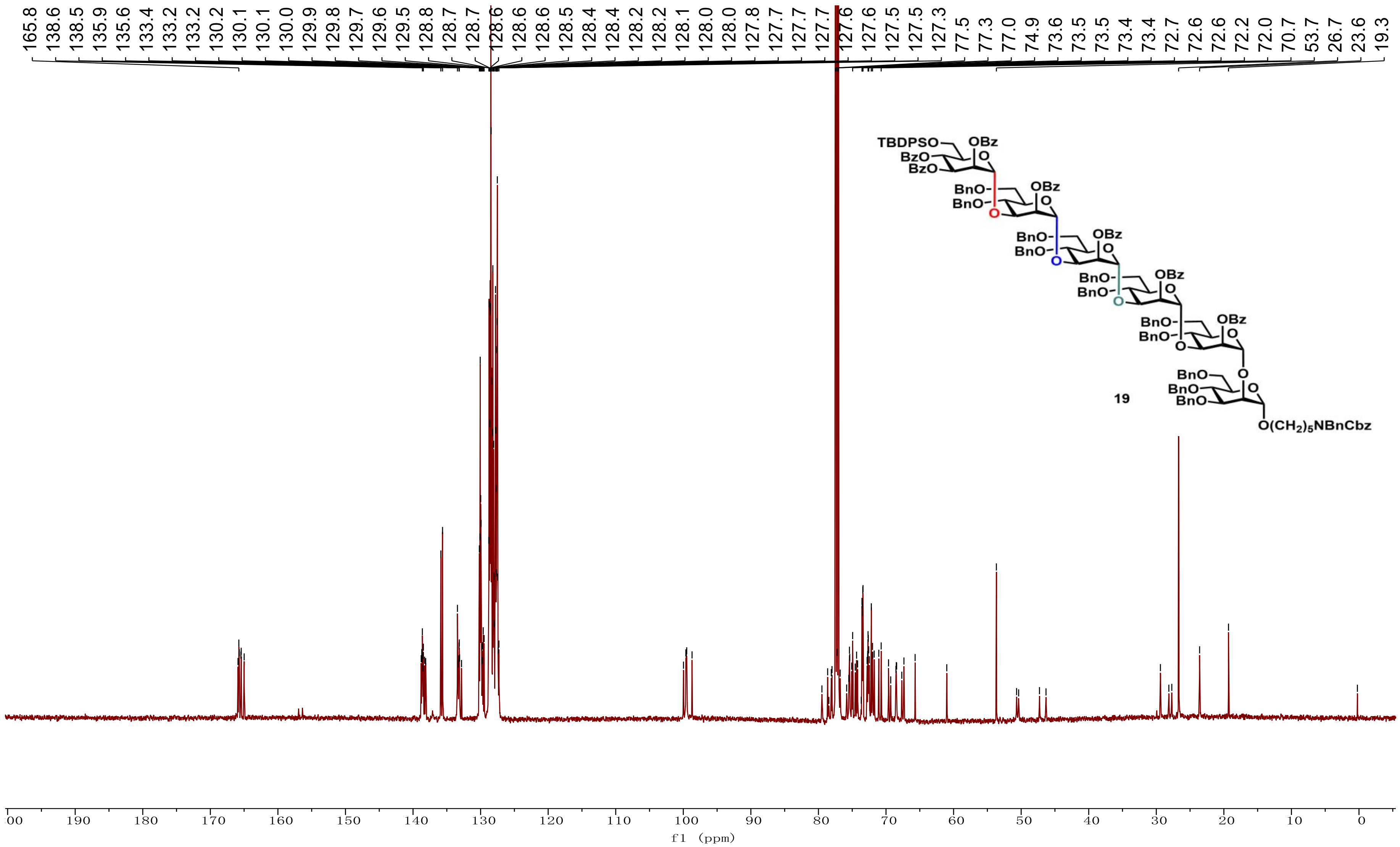
165.8 138.6 138.5 135.9 135.6 133.4 133.2 133.2 130.2 130.1 130.1 130.0 129.9 129.8 129.7 129.6 129.5 128.8 128.7 128.7 128.6 128.6 128.6 128.5 128.4 128.4 128.2 128.2 128.1 128.0 128.0 127.8 127.7 127.7 127.7 127.6 127.6 127.5 127.5 127.3 77.5 77.3 77.0 74.9 73.6 73.5 73.5 73.4 73.4 72.7 72.6 72.6 72.2 72.0 70.7 53.7 26.7 23.6 19.3
TBDPSO
OBz
BzO
BzO
BnO
OBz
BnO
19
O(CH2)5NBnCbz
200 190 180 170 160 150 140 130 120 110 100 90 80 70 60 50 40 30 20 10 0
f1 (ppm)

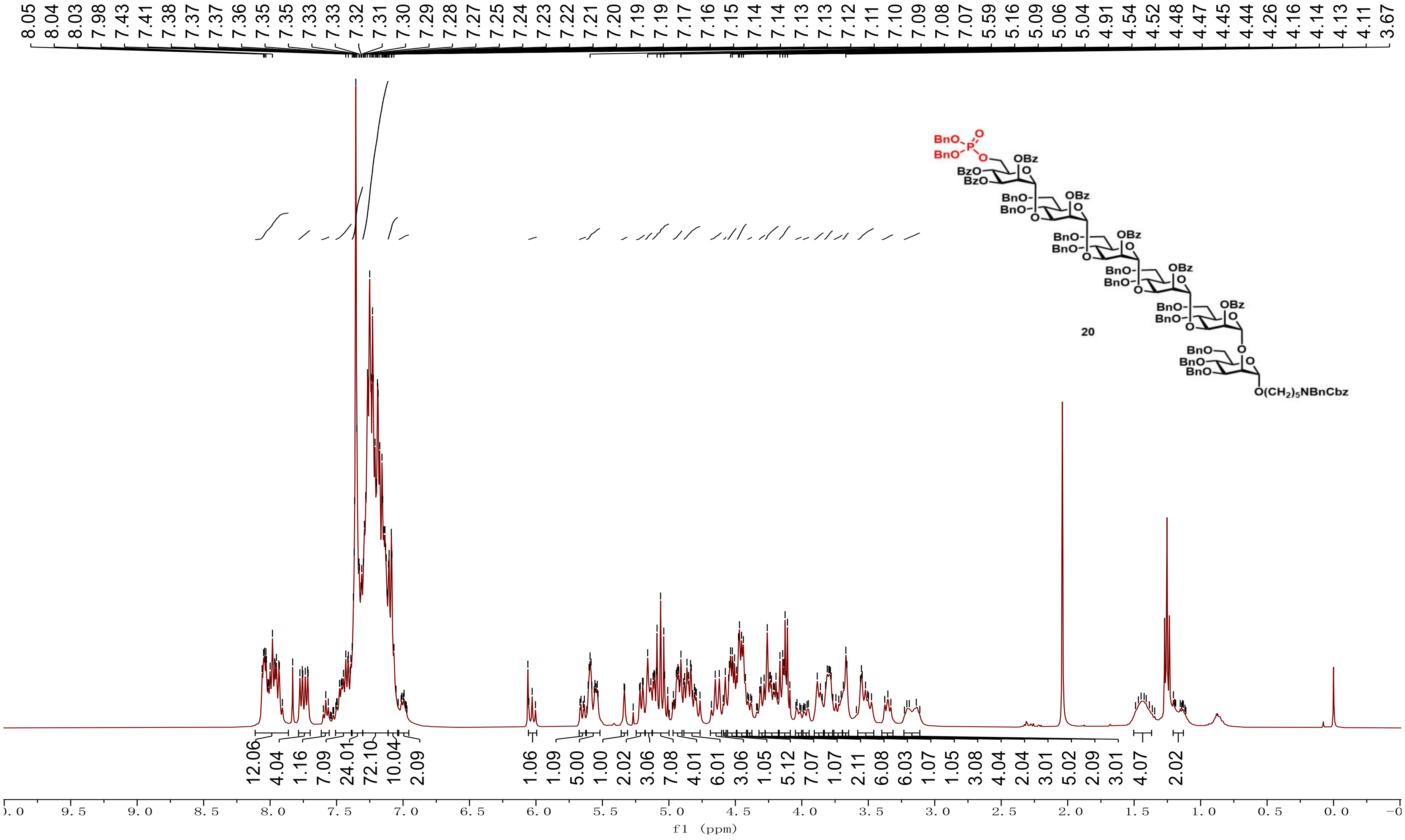
8.05 8.04 8.03 7.98 7.43 7.41 7.38 7.37 7.37 7.36 7.35 7.35 7.33 7.33 7.32 7.31 7.30 7.29 7.28 7.27 7.25 7.24 7.23 7.22 7.21 7.20 7.19 7.19 7.17 7.16 7.15 7.14 7.14 7.13 7.13 7.12 7.11 7.10 7.09 7.08 7.07 5.59 5.16 5.09 5.06 5.04 4.91 4.54 4.52 4.48 4.47 4.45 4.44 4.26 4.16 4.14 4.13 4.11 3.67
12.06 4.04 1.16 7.09 24.01 72.10 10.04 2.09 1.06 1.09 5.00 1.00 2.02 3.06 7.08 4.01 6.01 3.06 1.05 5.12 7.07 1.07 2.11 6.08 6.03 1.07 1.05 3.08 4.04 2.04 3.01 5.02 2.09 3.01 4.07 2.02
BnO
BnO
O
P
O
OBz
BzO
BzO
O
BnO
BnO
OBz
O
BnO
BnO
OBz
O
BnO
BnO
OBz
O
BnO
BnO
OBz
O
20
BnO
BnO
BnO
O
O(CH2)5NBnCbz
10.0 9.5 9.0 8.5 8.0 7.5 7.0 6.5 6.0 5.5 5.0 4.5 4.0 3.5 3.0 2.5 2.0 1.5 1.0 0.5 0.0
f1 (ppm)

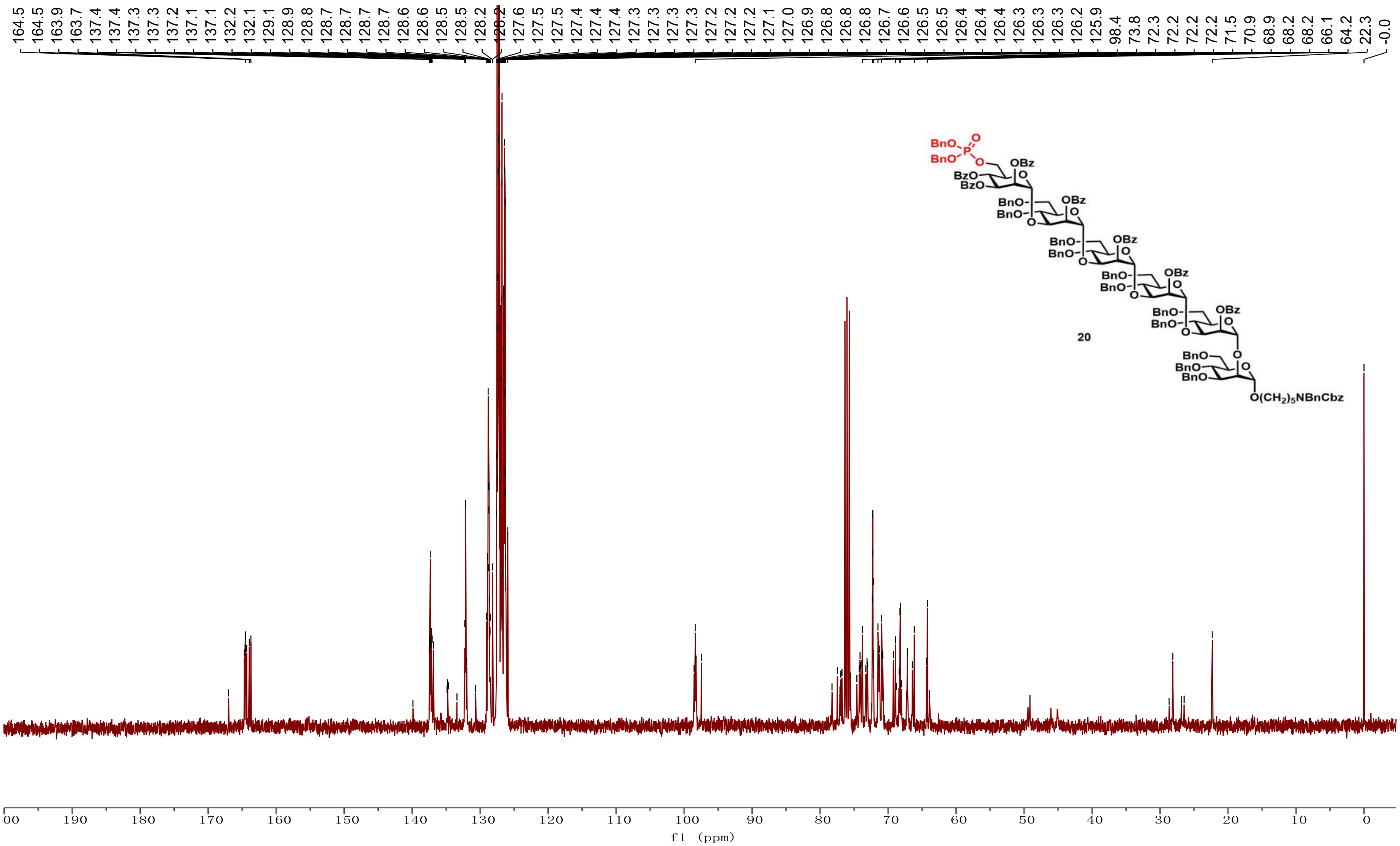

164.5 164.5 163.9 163.7 137.4 137.4 137.3 137.3 137.2 137.1 137.1 132.2 132.1 129.1 128.9 128.8 128.7 128.7 128.7 128.7 128.6 128.6 128.5 128.5 128.2 128.2 127.6 127.5 127.5 127.4 127.4 127.4 127.3 127.3 127.3 127.3 127.2 127.2 127.2 127.1 127.0 126.9 126.8 126.8 126.8 126.7 126.6 126.5 126.5 126.4 126.4 126.4 126.3 126.3 126.3 126.2 125.9 98.4 73.8 72.3 72.2 72.2 72.2 71.5 70.9 68.9 68.2 68.2 66.1 64.2 22.3 -0.0
BnO
BnO
P
O
OBz
BzO
BzO
BnO
BnO
20
O(CH2)5NBnCbz
00 190 180 170 160 150 140 130 120 110 100 90 80 70 60 50 40 30 20 10 0
f1 (ppm)

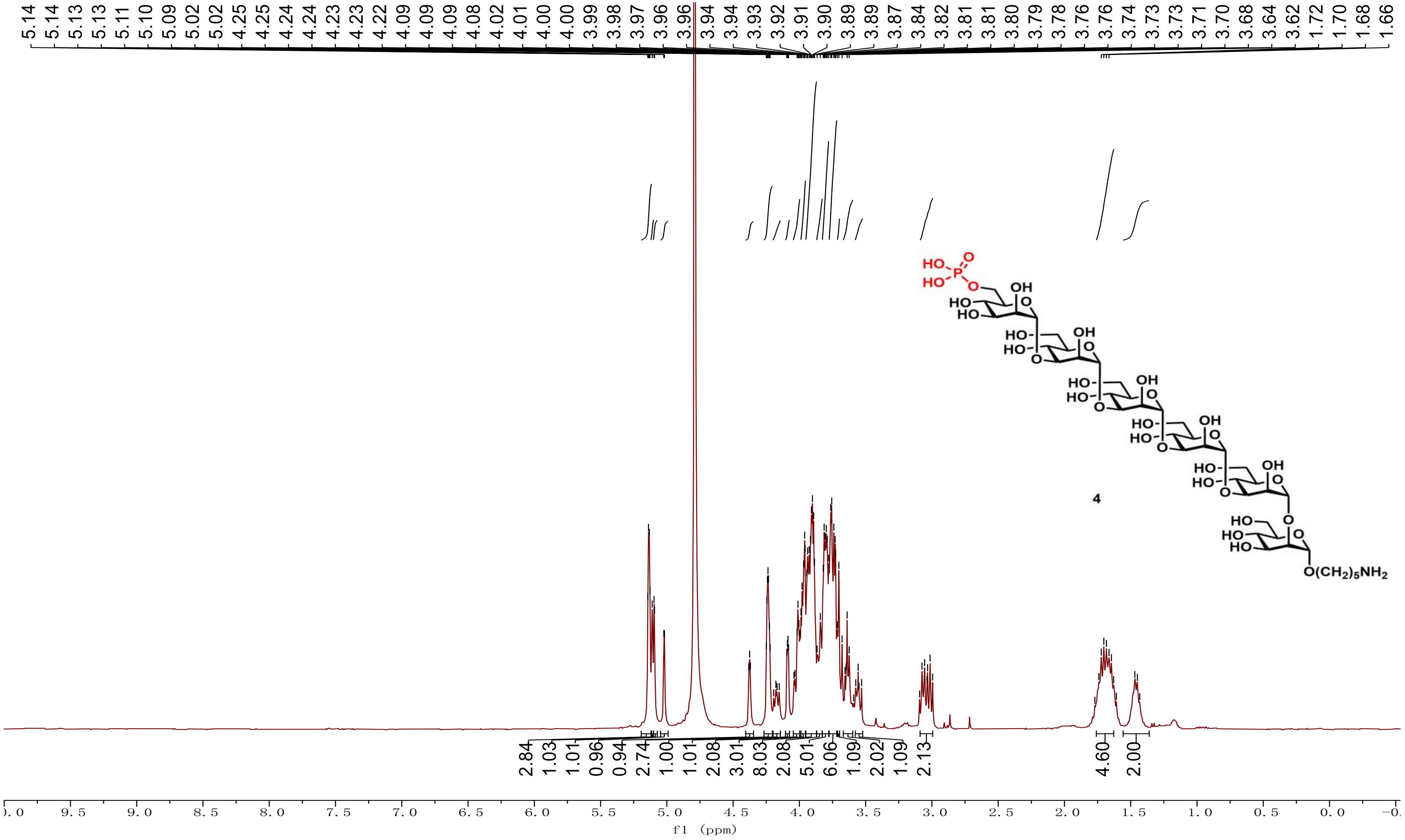
5.14 5.14 5.13 5.13 5.11 5.10 5.09 5.02 5.02 4.25 4.25 4.24 4.24 4.23 4.23 4.22 4.09 4.09 4.09 4.08 4.02 4.01 4.00 4.00 3.99 3.98 3.97 3.96 3.96 3.94 3.94 3.93 3.92 3.91 3.90 3.89 3.89 3.87 3.84 3.82 3.81 3.81 3.80 3.79 3.78 3.76 3.76 3.74 3.73 3.73 3.71 3.70 3.68 3.64 3.62 1.72 1.70 1.68 1.66
2.84 1.03 1.01 0.96 0.94 2.74 1.00 1.01 2.08 3.01 8.03 2.08 5.01 6.06 1.09 2.02 1.09 2.13 4.60 2.00
4
O(CH2)5NH2
f1 (ppm)

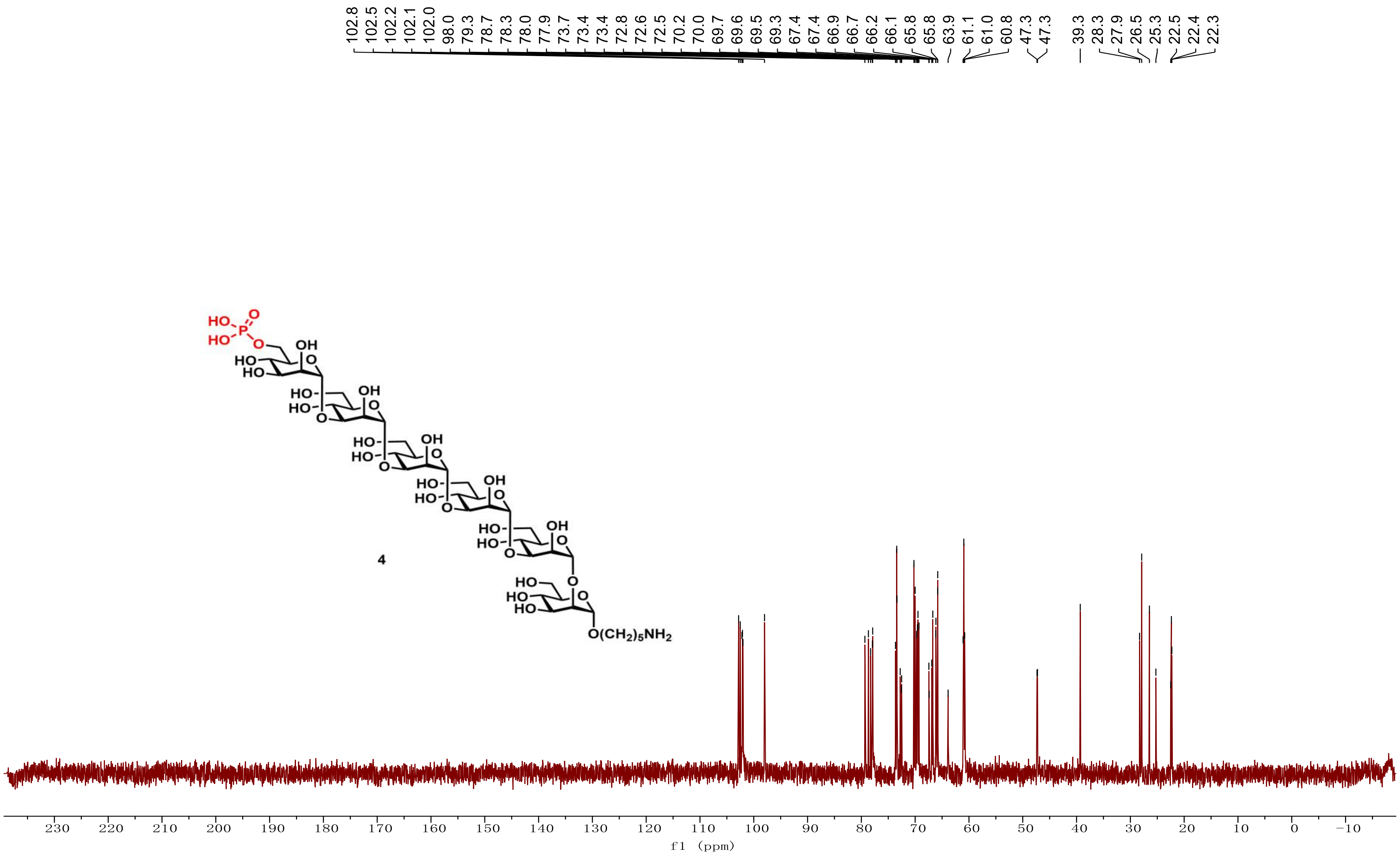
102.8
102.5
102.2
102.1
102.0
98.0
79.3
78.7
78.3
78.0
77.9
73.7
73.4
73.4
72.8
72.6
72.5
70.2
70.0
69.7
69.6
69.5
69.3
67.4
67.4
66.9
66.7
66.2
66.1
65.8
65.8
63.9
61.1
61.0
60.8
47.3
47.3
39.3
28.3
27.9
26.5
25.3
22.5
22.4
22.3
4
O(CH2)5NH2
230 220 210 200 190 180 170 160 150 140 130 120 110 100 90 80 70 60 50 40 30 20 10 0 −10
f1 (ppm)

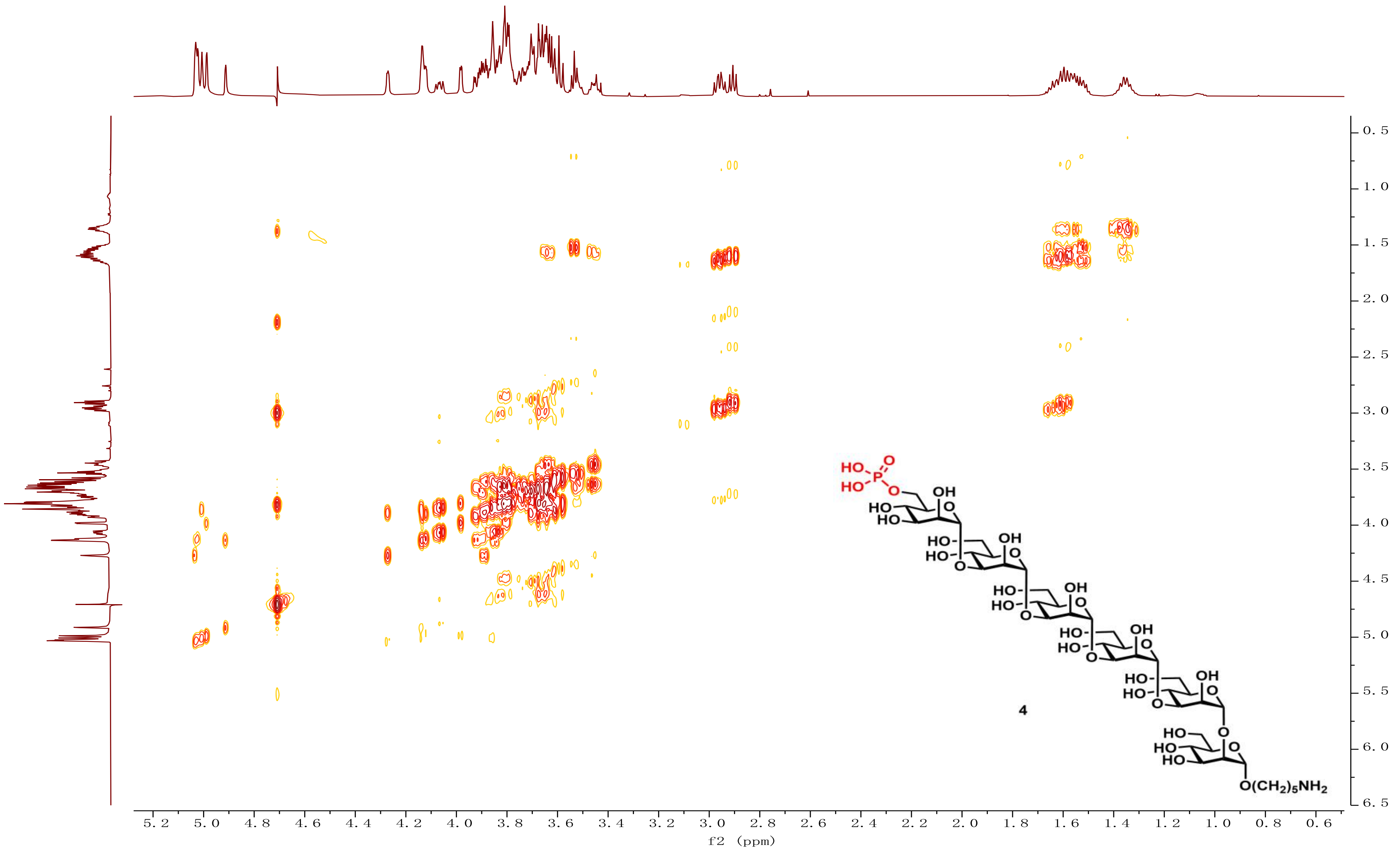
f2 (ppm)
f1 (ppm)
5.2
5.0
4.8
4.6
4.4
4.2
4.0
3.8
3.6
3.4
3.2
3.0
2.8
2.6
2.4
2.2
2.0
1.8
1.6
1.4
1.2
1.0
0.8
0.6
0.5
1.0
1.5
2.0
2.5
3.0
3.5
4.0
4.5
5.0
5.5
6.0
6.5
4
O(CH2)5NH2

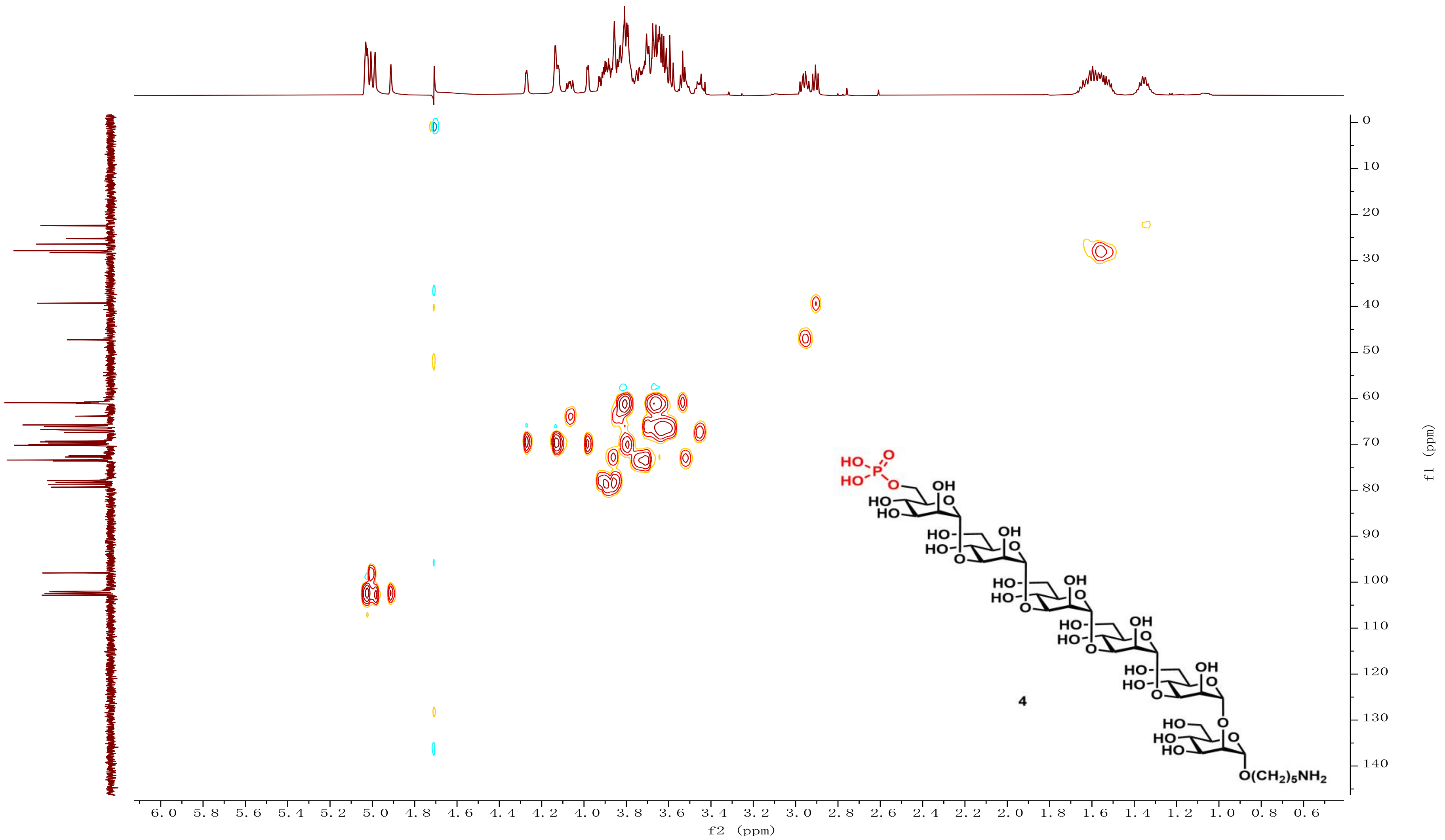
f2 (ppm)
f1 (ppm)
HO
HO
P
O
OH
4
O(CH2)5NH2
